# Photoresponsive *Metallo*‐Supramolecular Systems Constructed From a Bidentate Ligand

**DOI:** 10.1002/marc.70267

**Published:** 2026-03-22

**Authors:** Luca Bertossi, Carina Lin, Davide M. De Luca, Marta Oggioni, Georges J. M. Formon, Christoph Weder

**Affiliations:** ^1^ Adolphe Merkle Institute Polymer Chemistry and Materials University of Fribourg Fribourg Switzerland

**Keywords:** metallosupramolecular, photo‐responsive, stimuli‐responsive

## Abstract

Metallosupramolecular polymers (MSPs) are stimuli‐responsive materials whose properties can be tailored through the choice of ligand and metal salt. Their dynamic behavior is governed by the reversibility of the metal‐ligand (ML) complexes and the ability to shift the equilibrium between assembled and disassembled states, enabling functions such as recyclability, healability, and adaptability. While heat is a simple and efficient stimulus, it makes temporal and spatial control difficult to achieve. To address this, we developed MSP systems composed of copolymers bearing 6‐(1′‐methylbenzimidazolyl)pyridine (MBP) ligands in varying concentrations, metal salts, and the photoacid generator (PAG) 2‐(4‐methoxystyryl)‐4,6‐bis(trichloromethyl)‐1,3,5‐triazine (MBTT). The MBP groups coordinate Fe(OTf)_2_ or Fe(OTf)_3_ to form dynamic ML cross‐links. The resulting networks produce robust organogels when imbibed with chlorobenzene. MBTT renders the systems optically responsive, as UV light triggers the release of acid, which protonates and dissociates the ML cross‐links, thereby inducing network disassembly. We characterize the MSP organogels using rheology and monitor acid‐induced responses via UV–vis spectroscopy on model complexes. Opto‐rheological experiments reveal that softening is possible with as little as 0.25 equivalents of MBTT per ML complex and that excellent temporal control is possible.

## Graphical abstract

Metallosupramolecular polymer organogels based on dynamic metal–ligand (ML) cross‐links are rendered optically responsive by incorporating a photoacid generator. Exposing these systems to UV light releases an acid that protonates the ligands, causing network disassembly and thereby softening. This strategy enables efficient, temporally precise light‐controlled modulation of mechanical properties at low photoacid loadings.

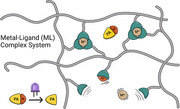

## 1 Introduction

**FIGURE 1:**
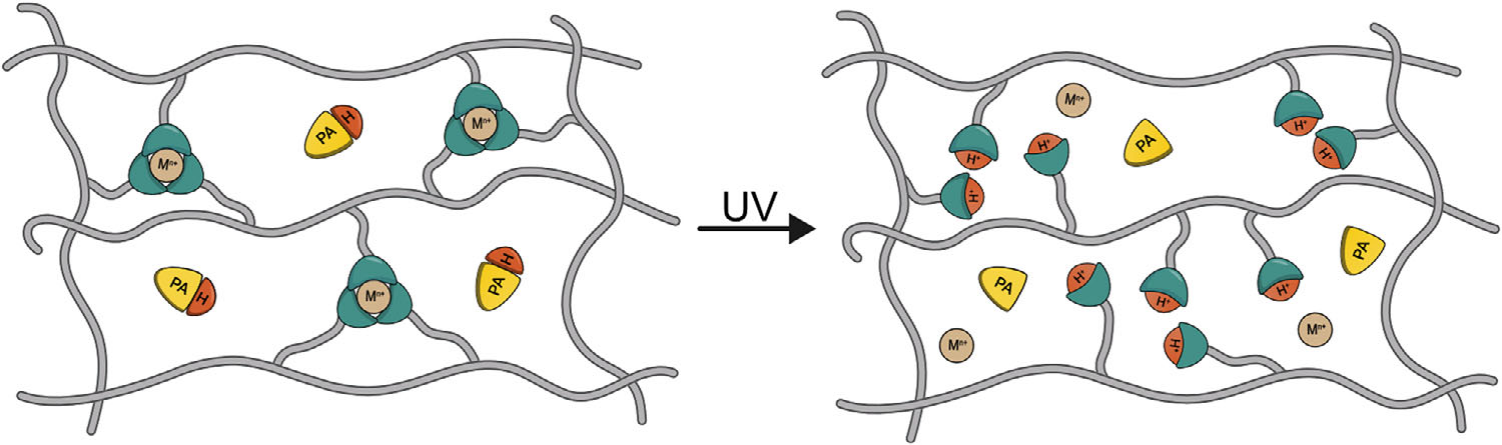
Schematic representation of the metallosupramolecular systems investigated here. Densely cross‐linked MSP networks formed via metal‐ligand (ML) complexes with a coordination number of three are combined with a photoacid generator (PAG). Upon UV irradiation, the PAG releases a strong acid, which protonates the ligand and dissociates the ML complexes.

Polymers capable of transitioning from a defined initial state to a desired functional state in response to external triggers are referred to as stimuli‐responsive. These materials have shown great promise in various applications, including drug delivery, soft robotics, sensors, and smart coatings [1, 2, 3, 4, 5, 6, 7, 8]. Numerous approaches have been used to endow polymers with stimuli‐responsive behavior [9]. Among these, the use of supramolecular chemistry has emerged as a particularly promising strategy to create dynamic and adaptive materials [8, 10, 11, 12, 13, 14, 15, 16, 17]. The dynamic equilibrium between the bound and unbound states of supramolecular linkers imparts polymers with the capacity for chain extension or crosslinking under ambient conditions. To achieve this, various reversible binding motifs, including hydrogen bonding [18, 19, 20, 21], metal‐ligand coordination [22, 23, 24], and host‐guest interactions [25, 26, 27] have been strategically incorporated into polymer architectures: upon exposure to external stimuli, these interactions can temporarily dissociate, leading to changes in molecular weight and topology. Such changes, in turn, modulate the material's macroscopic properties in response to environmental cues. Stimuli that have been used to elicit responses in such materials include heat [23, 28, 29], light [8, 22, 30, 31], chemicals [32, 33], electric currents [34, 35, 36], magnetic fields [37], or mechanical forces [38, 39]. Functions such as stimuli‐induced healing [22, 40, 41], actuation [42, 43, 44], recycling [45], and sensing [32, 46] have been achieved in these materials. Heat is arguably the most commonly used trigger, however, the level of spatiotemporal control, especially, in comparison to light, is poor [8, 47, 48]. To address this limitation, we recently reported light‐responsive supramolecular systems, comprising a supramolecular polymer and an auxiliary molecular trigger, whose function is based on optochemical transduction mechanisms [49, 50]. More specifically, we combined supramolecular networks in which reversible cross‐links are formed by either dimerized, hydrogen‐bonded ureidopyrimidinone (UPy) groups or zinc complexes of the 2,6‐bis(1′‐methyl‐benzimidazolyl)‐pyridine (Mebip) ligand, with a photoacid generator (PAG) that serves as an optochemical transducer [49, 50]. The PAG is activated by UV light, releasing hydrochloric acid (HCl), which protonates the UPy groups or the Mebip ligand, thereby breaking the cross‐links and altering the mechanical properties of the systems. Optorheological experiments reveal that the rheological properties of organogels based on metallosupramolecular (MSP) networks and the PAG 2‐(4‐methoxystyryl)‐4,6‐bis(trichloromethyl)‐1,3,5‐triazine (MBTT) can be changed on demand by exposure to UV, facilitating optically controlled gel‐sol transitions [49, 50]. While our first‐generation MSP systems were based on poly(acrylates) that comprise the Mebip ligand, which was selected because it displays high binding constants with transition metals and affords a robust “on” state, we speculated that the lower binding constant of the bidentate analogue, 6‐(1'‐methyl‐benzimidazolyl)‐pyridine (MBP) could render such systems more responsive, as they are easier to dissociate [51]. We also surmised that this process would be further facilitated by the fact that the MBP motif can, as previous titration data show, only be protonated once, while the Mebip ligand can bind two protons. Finally, we considered cross‐links based on metal‐ligand complexes with different coordination numbers and binding strengths to control the topology and dynamics of MSPs that serve as the basis for such systems [52, 53]. To answer whether MBP can be used to create robust yet easily switchable MSP systems, we investigated how different metal salts affect the association and coordination number of MBP‐based ML complexes through model studies using UV–vis spectroscopy. We utilized this knowledge to develop a series of MSPs and investigated their properties through shear rheology experiments. We explored the acid sensitivity of the ML complexes of interest, created MSP systems by incorporating the PAG MBTT into such gels (Figure 1), and investigated their light‐induced disassembly.

## 2 Results and Discussion

### 2.1 Binding Studies of an MBP Model Compound and Different Metal Salts

**FIGURE 2:**
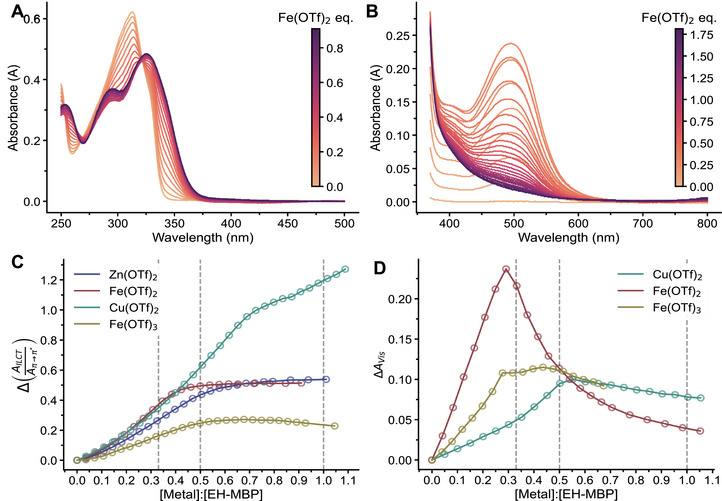
Titrations of MeCN solutions of **EH‐MBP** with aliquots of different metal salts monitored by UV–vis absorption spectroscopy. (A,B) Absorption spectra recorded upon addition of (A) Fe(OTf)_2_ (c = 0.61 mm) to **EH‐MBP** (c = 24 µm) and (B) Fe(OTf)_2_ (c = 6.6 mm) to **EH‐MBP** (c = 240 µm). Note the difference in concentration. (C) Line graph showing the change of the ratio of the absorbances at 350 nm (Shifted ILCT band of **Zn(EH‐MBP)_3_**, **Fe(II)(EH‐MBP)_3,_** and **Fe(III)(EH‐MBP)_3_**) or 345 nm (**Cu(II)(EH‐MBP)_3_**) and 313 nm (π → π* band of **EH‐MBP**) versus the [metal]:[**EH‐MBP**] ratio for [metal] = 0.61 mm and [**EH‐MBP**] = 24 µm. (D) Line graph showing the change of absorbance at 495 nm (MLCT band of **Fe(II)(EH‐MBP)_3_**), 450 nm (LMCT/d‐d transition band of **Fe(III)(EH‐MBP)_3_**), and 725 nm (d‐d transition band of **Cu(II)(EH‐MBP)_3_**) versus the [metal]:[**EH‐MBP**] ratio for [metal] = 6.6 mm and [**EH‐MBP**] = 240 µm.

The association constant and topology of the ML complexes significantly influence the properties and dynamic behavior of MSP networks. To explore these effects, we first investigated the coordination of MBP with various metal ions using the soluble, ethylhexyl functionalized 6‐(1′‐methyl‐benzimidazolyl)‐pyridine‐3‐ol (**EH‐MBP,** Figure S1) as a model compound for MBP. Complex formation was monitored via UV–vis absorption spectroscopy, which revealed changes in the intra‐ligand charge transfer (ILCT) band of **EH‐MBP** from primarily a π → π* transition to perturbations caused by metal coordination [54]. This results in a reduction of the original π → π* maximum and a concomitant increase in bands at slightly lower energies (+ ca. 40 nm) whose position varies slightly depending on the metal ion introduced (Figure 2). To distinguish the influence of the metal ion from effects that the anionic counterion may induce, all experiments were conducted using triflate (OTf^−^) salts, as the triflate is a weakly coordinating counterion. Based on previous findings that transition metals yield mechanically robust MSPs, we selected Zn(OTf)_2_, Fe(OTf)_2_, Fe(OTf)_3_, and Cu(OTf)_2_ for evaluation [51]. Zn(OTf)_2_ was included due to its previously demonstrated ability, in combination with Mebip, to balance mechanical robustness with dynamic responsiveness [22]. The cation Fe^2^^+^ was chosen for its stronger binding affinity to MBP, while Fe^3^^+^ was examined to assess the influence of increased acidity and metal “hardness” on MBP association [55]. Cu^2^^+^ was investigated for its adaptable coordination behavior previously observed in Mebip‐based complexes [56, 57]. Each salt was titrated into a 24 µm **EH‐MBP** solution in acetonitrile (MeCN), and UV–vis absorption spectra were recorded (Figure 2a,b; Figure S2). The spectra reveal a decrease of the π → π* band of **EH‐MBP** at 313 nm and a simultaneous increase of a band at 350 nm (**Zn(EH‐MBP)_3_**, **Fe(II)(EH‐MBP)_3,_** and **Fe(III)(EH‐MBP)_3_**) or 345 nm (**Cu(II)(EH‐MBP)_3_**). The spectra recorded for Zn(OTf)_2_, Fe(OTf)_2_, and Fe(OTf)_3_ show clear isosbestic points, each indicating clean interconversion between two well‐defined species, while the spectra recorded for Cu(OTf)_2_ are less straightforward, potentially due to the formation of multiple species or more complex equilibria. The change of the ratio of the absorbances at the maxima of the respective lower‐energy band and the ligand's π→ π* band was plotted versus the [metal]:[**EH‐MBP**] ratio (Figure 2c). The resulting traces all show an initial inflection near a [metal]:[**EH‐MBP**] ratio of 0.33, and the ones of Fe(OTf)_2_ and Fe(OTf)_3_ reach distinct endpoints at a ratio of 0.5. In contrast, the titrations of Zn(OTf)_2_ and Cu(OTf)_2_ show less pronounced endpoints, suggesting weaker, more dynamic binding, especially in the case of Cu(OTf)_2_, where even at a [metal]:[**EH‐MBP**] ratio of 1.1, no endpoint is reached.

As the analysis of the data in the UV regime is complicated by the overlap of ILCT bands splitting and shifting, we also considered the MLCT/LMCT/d‐d transition bands in the visible region, which are observable for Fe(OTf)_2_, Fe(OTf)_3_, and Cu(OTf)_2_ but not Zn(OTf)_2_ [58, 59, 60, 61]. As their absorbance is too low to be reliably monitored at the original **EH‐MBP** concentration (c = 24 µm), we repeated the titrations at higher concentrations (Figure 2b,d; Figure S3, c = 240 µm), which also drive the equilibrium toward complex formation. In the case of Fe(OTf)_2_, a plot of the change in MLCT absorbance against the [metal]:[**EH‐MBP**] ratio reaches a maximum at [metal]:[**EH‐MBP**] = ca. 0.33, indicating initial complexation with a coordination number of 3 (ML_3_). In the case of Fe(OTf)_3_ and Cu(OTf)_2_, inflection points at [metal]:[**EH‐MBP**] of ca. 0.33 and 0.5 are seen, suggesting the formation of both ML_3_ and ML_2_ complexes. In all cases, absorbance changes are observed beyond the last inflection point, indicating dynamic exchange equilibria that extend beyond initial binding. To further elucidate the binding stoichiometry and coordination geometry, ^1^H‐NMR and mass spectrometry (ESI‐MS) experiments were conducted.

Analogous to the UV–vis studies, titrations were monitored with ^1^H‐NMR spectroscopy upon the addition of Zn(OTf)_2_ and Fe(OTf)_2_ to a 44 mm solution of **EH‐MBP** in CD_3_CN:CDCl_3_ (8:2) (Figures S4 and S5). Due to the paramagnetic nature of Cu^2+^ and Fe^3+^, such ^1^H‐NMR experiments were not possible for these complexes. During ^1^H‐NMR‐monitored titrations with Zn^2+^ and Fe^2+^, the resonances corresponding to the **EH‐MBP** ligand exhibited significant broadening and progressive chemical shifts upon successive metal additions, signaling the onset of complexation. For both salts, peaks pertaining to the free ligand disappear at a [metal]:[**EH‐MBP**] ratio of ca. 0.33, indicating the quantitative formation of ML_3_ complexes. In the case of Zn(OTf)_2_, the ^1^H‐NMR spectra continue to change, and the complex peaks continue to shift as the [metal]:[**EH‐MBP**] is further increased to ca. 0.5, suggesting the transition toward a lower‐order ML_2_ complex (Figure S4). This observation highlights the dynamic nature of the ML bond, corroborated by UV–vis data. Conversely, in the case of Fe(OTf)_2_, increasing the [metal]:[**EH‐MBP**] ratio beyond 0.33 equivalents resulted in severe shimming instability, likely due to the presence of magnetically susceptible ions or clusters, which prevented further NMR acquisition (Figure S5).

Mass spectrometry (ESI‐MS) was employed to further validate these findings using 3 mm solutions of **EH‐MBP** in MeCN:CHCl_3_ (9:1) to which 0.33 and 0.5 equivalents of the respective metal salts were added (Figures S6–S9). At a [metal]:[**EH‐MBP**] ratio of 0.33, ML_3_ complexes were predominantly observed for Cu^2+^, Zn^2+^, and Fe^2+^. In contrast, the Fe^3+^ sample exhibited only the free ligand, suggesting that the complex is prone to fragmentation under ionization conditions (Figure S8). For solutions containing a [metal]:[**EH‐MBP**] ratio of 0.5, mixtures of ML_2_ and ML_3_ species were identified for Cu^2+^, Zn^2+^, and Fe^2+^, confirming the ML bond dynamicity and the underlying preference for ML_3_ coordination geometries, thus corroborating the UV–vis and ^1^H‐NMR investigations. Building on these binding studies, we sought to determine how the binding behavior of MBP‐based ML complexes translates to the shear mechanical properties of organogels constructed from MSP networks cross‐linked by these complexes (vide infra).

### 2.2 Preparation and Characterization of MSP Organogels

**SCHEME 1:**
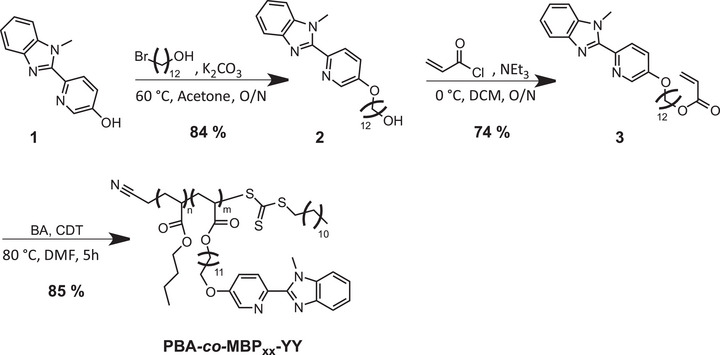
Synthesis of the MBP‐acrylate (**3**) and the **PBA**‐*co*‐**MBP_xx_‐YY** copolymers. The subscript XX indicates the mol fraction of **MBP** and YY the *M*_n_ (in kg/mol) of the copolymer.

MBP‐acrylate (**3**) was synthesized from 6‐(1’‐methyl‐benzimidazolyl)‐pyridin‐3‐ol (**1**) by adapting the route utilized in our previous study [51], i.e., alkylation of (**1**) with 12‐bromo‐1‐dodecanol and esterification of the resulting (**2**) with acryloyl chloride (Scheme 1). The resulting monomer (**3**) was obtained with a higher overall average yield (74%) than the corresponding Mebip monomer and exhibits better solubility, which simplifies the purification process from dialysis to a series of precipitations [49]. Reversible addition‐fragmentation chain transfer (RAFT) polymerizations were carried out to prepare statistical copolymers of *n*‐butyl acrylate (BA) and MBP acrylate (**3**), i.e., poly(*n*‐butyl acrylate‐*co*‐MBP‐acrylate) (Scheme 1). A trithiocarbonate‐based RAFT agent, 2‐cyano‐2‐propyl dodecyl trithiocarbonate (CDT), was used in conjunction with AIBN as a thermal initiator in DMF at 80°C [49, 62, 63, 64]. A series of these copolymers was prepared by systematically altering the number‐average molecular weight (*M*_n_) between ca. 16 000‐56 000 g/mol and the MBP‐acrylate fraction from 5 to 10 mol%. The resulting copolymers are referred to as **PBA**‐*co*‐**MBP_xx_‐YY**, where the subscript XX indicates the mol fraction of **MBP** and YY the *M*_n_ (in kg/mol) of the copolymer. The copolymers were characterized using size‐exclusion chromatography (SEC, Figures S10 and S11), ^1^H‐NMR spectroscopy, and diffusion‐ordered NMR spectroscopy (DOSY, Figures S22–S25). The analysis of the data shows that the *M*_n_ can readily be varied as targeted, that the mol fraction of MBP‐acrylate incorporated in the copolymer corresponds well to that of the feed (Table 1), and that the incorporation of **MBP** into the polymers is statistical. Except for **PBA**‐*co*‐**MBP_10_‐56**, the copolymer with the highest *M*_n_ and **MBP** fraction, the dispersity (*Đ*) is low (≤ 1.3), suggesting that these polymerization reactions are controlled. Given that *Đ* of **PBA**‐*co*‐**MBP_5_‐46** is low, and that previous studies with the corresponding Mebip comonomer also show an increase of *Đ* when the ligand concentration reaches 10 mol% [49, 62], we speculate that the loss of control is imparted by the ligand.

**TABLE 1:** Molecular characterization of the poly(*n*‐butyl acrylate‐*co*‐MBP acrylate)s prepared.

Polymer	*M*_n_ (g/mol)^a^	[MBP] in polymer (mol%)^b^	[MBP] in feed (mol%)^c^	*Đ* ^a^	MBP/Chain
**PBA** *‐co‐* **MBP_5_‐16**	15’800	4.7	5.0	1.2	5
**PBA** *‐co‐* **MBP_5_‐28**	28’200	4.9	5.0	1.3	10
**PBA** *‐co‐* **MBP_5_‐46**	46’200	4.6	5.0	1.3	15
**PBA** *‐co‐* **MBP_10_‐56**	55’900	10.5	10.0	2.3	36

^a^ Measured by size exclusion chromatography (SEC).

^b^ Measured by ^1^H‐NMR spectroscopy.

^c^ Calculated from the feed composition.

The MSP networks were prepared in glass vials by adding freshly prepared solutions of the metal salts (*c*≈0.057 m) in MeCN to dilute solutions (*c* = 0.03 g/mL) of **PBA**‐*co*‐**MBP_xx_‐YY** in CHCl_3_. The solvent was then allowed to evaporate slowly overnight, and a well‐defined amount of chlorobenzene was introduced to swell the MSPs and produce organogels. This solvent was selected because it has a high boiling point and readily swells PBA networks [49]. The amount of chlorobenzene was varied to produce gels in which the mass of dry MSP corresponds to between 15 and 25 wt% of the solvent mass.

**FIGURE 3:**
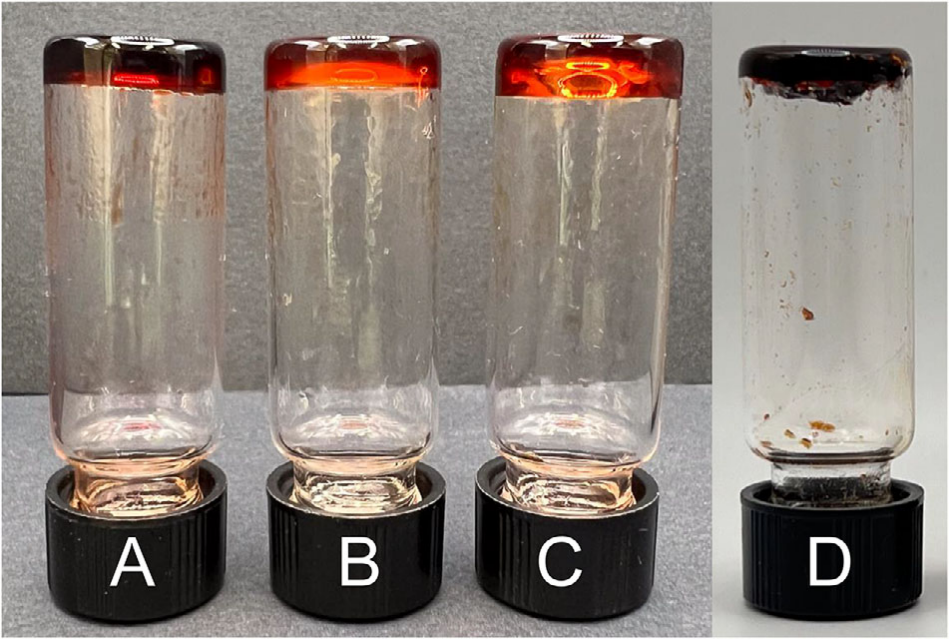
Pictures of MSP gels. The samples contain 25 wt.% of the respective MSP relative to the mass of chlorobenzene. (A) **Fe(II)(PBA**‐*co*‐**MBP_5_‐46)‐1:3**, (B) **Fe(II)(PBA**‐*co*‐**MBP_5_‐46)‐1:2**, (C) **Fe(III)(PBA**‐*co*‐**MBP_5_‐46)‐1:3**, and (D) **Fe(III)(PBA**‐*co*‐**MBP_10_‐56**)‐**1:3**.

Initially, four different triflate salts were used to form MSPs, including Fe(OTf)_2_, Fe(OTf)_3_, Zn(OTf)_2_, and Cu(OTf)_2_ (Table 2 and Figure 3). All of the **PBA**‐*co*‐**MBP_xx_‐YY** copolymers were combined with these salts to screen how the *M*_n_ and the ligand fraction of the copolymer and the ligand‐to‐metal ratio affect the formation of MSP gels. Attempts to produce gels with **PBA**‐*co*‐**MBP_5_‐16** and **PBA**‐*co*‐**MBP_5_‐28** were unsuccessful for all compositions, producing more or less viscous liquids instead (confirmed via the inverted vial test), reflecting that the ML complexes are highly dynamic and that copolymers with a higher *M_n_* (and therewith a higher number of MBP residues per macromolecule, cf. Table 1) or a higher ligand fraction are needed to form gels. When MSPs based on **PBA**‐*co*‐**MBP_5_‐46** or **PBA**‐*co*‐**MBP_10_‐56** and Zn^2+^or Cu^2+^ (combined in a 1:3 M:L ratio) were swol with chlorobenzene (25 wt%), highly viscous liquids were obtained, likely on account of the low binding constants and high dynamicity of the resulting complexes; this is corroborated by titrations discussed above (Figure 2). By contrast, four of the iron‐based MSP networks—**Fe(II)(PBA**‐*co*‐**MBP_5_‐46)‐1:3**, **Fe(II)(PBA**‐*co*‐**MBP_5_‐46)‐1:2**, **Fe(III)(PBA**‐*co*‐**MBP_5_‐46)‐1:3**, and **Fe(III)(PBA**‐*co*‐**MBP_10_‐56)‐1:3** afford robust gels upon swelling with chlorobenzene. In this notation, the prefix to the copolymer used refers to the metal cation in the MSP, and the suffix (‐M:L) indicates the ratio between the metal cation and ligand in the material. **Fe(II)(PBA**‐*co*‐**MBP_10_‐56)‐1:3** forms a dense gel that exhibits limited swelling, while **Fe(II)(PBA**‐*co*‐**MBP_5_‐46)‐1:3** also yields gels when the solid content is reduced to 20 wt.%. These results reflect more tightly cross‐linked network structures, consistent with the higher binding constants afforded by iron salts.

**TABLE 2:** Inverted vial tests of MSPs swelled with chlorobenzene.^a^

Metal^b^	PBA‐*co*‐MBP_5_‐46	PBA‐*co*‐MBP_10_‐56	Metal:Ligand ratio (mol/mol)
Zn^2+^	Liquid	Viscous liquid	1:3
Cu^2+^	Viscous liquid	Viscous liquid	1:3
Fe^2+^	Gel	Dense solid/poor swelling	1:3
Fe^2+^	Gel	—^c^	1:2
Fe^3+^	Gel	Gel	1:3

^a^ All attempts to produce gels were made with 25 wt.% MSP relative to the solvent.

^b^ All metal salts introduced were triflate salts.

^c^ Not determined.

### 2.3 Rheological Properties of MSP Organogels

**FIGURE 4:**
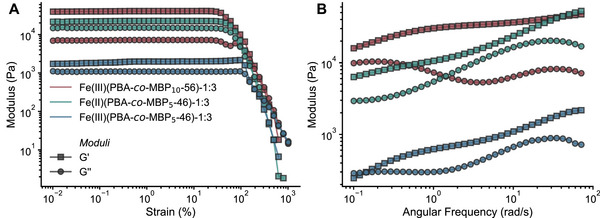
Small amplitude oscillatory shear (SAOS) rheology data of MSP gels **Fe(II)(PBA**‐*co*‐**MBP_5_‐46)‐1:3**, **Fe(III)(PBA**‐*co*‐**MBP_5_‐46)‐1:3**, and **Fe(III)(PBA**‐*co*‐**MBP_10_‐56**)‐**1:3**. (A) Amplitude sweeps conducted at an angular frequency *ω* = 10 rad/s. (B) Frequency sweeps conducted at a strain *γ* = 1%. All samples contain 25 wt% of the respective MSP in chlorobenzene.

Small amplitude oscillatory shear (SAOS) rheology was employed to characterize the viscoelastic properties of the three most robust Fe‐based gels according to the preliminary inversion vial test (Figure 3 and Table 2), which all feature a 1:3 metal‐to‐ligand ratio. Both amplitude (Figure 4a) and frequency sweep (Figure 4b) experiments were conducted.

The amplitude sweeps of the three gels show a broad viscoelastic region (LVE) and an onset of the non‐linear regime at critical strains of 40% or more, indicating that a fixed strain of *γ* = 1% and an angular frequency *ω* = 10 rad/s is well within the LVE. The storage, *G*′, and loss, *G*″, modulus values (measured at a strain of 1%) decrease in the order **Fe(III)(PBA**‐*co*‐**MBP_10_‐56**)‐**1:3** (*G*′ = 40.7 kPa, *G*″ = 7.2 kPa) > **Fe(II)(PBA**‐*co*‐**MBP_5_‐46)‐1:3** (*G*″ = 22.6 kPa, *G*″ = 15.0 kPa) > **Fe(III)(PBA**‐*co*‐**MBP_5_‐46)‐1:3** (*G*′ = 2.0 kPa, *G*″ = 1.1 kPa). This order is consistent with the relative binding strengths of the metal–ligand complexes, where Fe(II) exhibits stronger coordination than Fe(III) with MBP [65], but shows that the network cross‐linking density, which is governed by the mol fraction of MBP incorporated into the polymer, is a crucial parameter.

The frequency sweeps of the three gels, recorded at a fixed strain *γ* = 1%, confirm this relationship, revealing a higher storage modulus for **Fe(III)(PBA**‐*co*‐**MBP_10_‐56**)‐**1:3** (15.9 kPa < *G*′ < 47.3 kPa) compared with **Fe(II)(PBA**‐*co*‐**MBP_5_‐46)‐1:3** (6.3 kPa < *G*′ < 52.5 kPa) across the angular frequency range measured (0.1< *ω* < 100 rad/s). Crossover points (where *G*′ = *G*″), which mark the transition from elastic to viscous behavior, are observed at *ω*_cross_ = 0.14 rad/s for only **Fe(III)(PBA**‐*co*‐**MBP_10_‐46**)‐**1:3**, whereas **Fe(II)(PBA**‐*co*‐**MBP_5_‐46)‐1:3** and **Fe(III)(PBA**‐*co*‐**MBP_10_‐56**)‐**1:3** do not undergo this transition within the angular frequency range measured. For **Fe(II)(PBA**‐*co*‐**MBP_5_‐46)‐1:3** we also observe that *G*′and *G*″ approach each other ca. *ω* = 10 rad/s and then diverge at lower angular frequencies, but never cross. This suggests that there is an interplay between the binding strength of the ML complex and the dynamics or kinetics of exchange between ML cross‐links at larger timescales. Thus, the three Fe‐based MSP gels investigated are robust, exhibiting good resistance to shear deformation across a wide range of amplitudes and frequencies. We also carried out a frequency sweep with the 1:2 coordination gel **Fe(II)(PBA**‐*co*‐**MBP_5_‐46)‐1:2**, which exhibits a storage modulus in the range 0.044 kPa < *G*′ < 9.55 kPa and *ω*_cross_ = 25 rad/s, clearly indicating that this gel is much less robust than its 1:3 counterpart (Figure S12).

### 2.4 Dissociation of ML Complexes Upon Exposure to Acid

**FIGURE 5:**
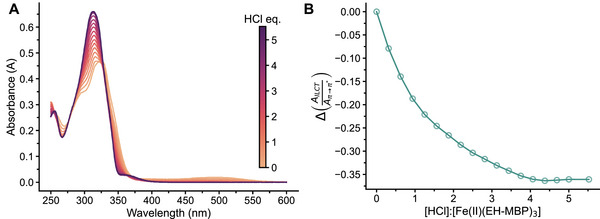
Titration of **Fe(II)(EH‐MBP)_3_** (c = 7.81 µm) with aliquots of HCl (c = 1.65 mm) in MeCN. (A) UV–vis absorption spectra recorded upon addition of HCl aliquots. (B) Line graph showing the change of the ratio of the absorbances at 350 nm (ILCT band of **Fe(II)(EH‐MBP)_3_**) and ca. 314 nm (π → π* band of the protonated ligand, the absorbance was recorded at the peak maximum) versus the [HCl]:[**Fe(II)(EH‐MBP)_3_**] ratio.

To verify the ability of acids to dissociate the metal–ligand complexes, titrations were conducted using **Fe(II)(EH‐MBP)_3_**, as this complex forms the cross‐links in the MSP gel **Fe(II)(PBA**‐*co*‐**MBP_5_‐46)‐1:3**, which was used to construct light‐switchable systems (vide infra), and HCl, which was selected as it proved most effective in dissociating complexes in prior studies [49]. The experiments were carried out with dilute (c = 7.81 µm, Figure 5) and concentrated (c = 78.1 µm, Figure S13) solutions of **Fe(II)(EH‐MBP)_3_** in MeCN, and the decomplexation caused by the addition of aliquots of HCl was monitored by UV–vis absorption spectroscopy. The spectra recorded for dilute solutions reveal the growth of an absorption band at ca. 314 nm, corresponding to the π→π* transition of the protonated free ligand, and the concomitant reduction in the ILCT band associated with **Fe(II)(EH‐MBP)_3_** at 350 nm (Figure 5a). The spectra recorded at higher concentration clearly show the decay of the MLCT band at ca. 495 nm (Figure S13a). Plots of the optical changes against the data show that under both conditions, ca. 4 equivalents of HCl are required to fully dissociate the **Fe(II)(EH‐MBP)_3_** complex (Figure 5b; Figure S13b). However, the optical signals, which were determined in analogy to the protocols applied to generate Figure 2 and Figure S2, decrease exponentially as a function of [HCl]:[**Fe(II)(EH‐MBP)_3_**], so that upon addition of 1 eq. of HCl, ca. 53% (low concentration, Figure 5b) or 57% (high concentration, Figure S13b) of the complexes have dissociated. These numbers exceed the fraction of comparable tridentate Mebip complexes that can be dissociated under similar conditions by a factor of two [49], confirming that MBP‐based systems indeed demonstrate greater responsiveness than the Mebip‐based analogues.

### 2.5 UV‐Actuated Softening of MBTT‐Loaded MSP Organogel Systems

**FIGURE 6:**
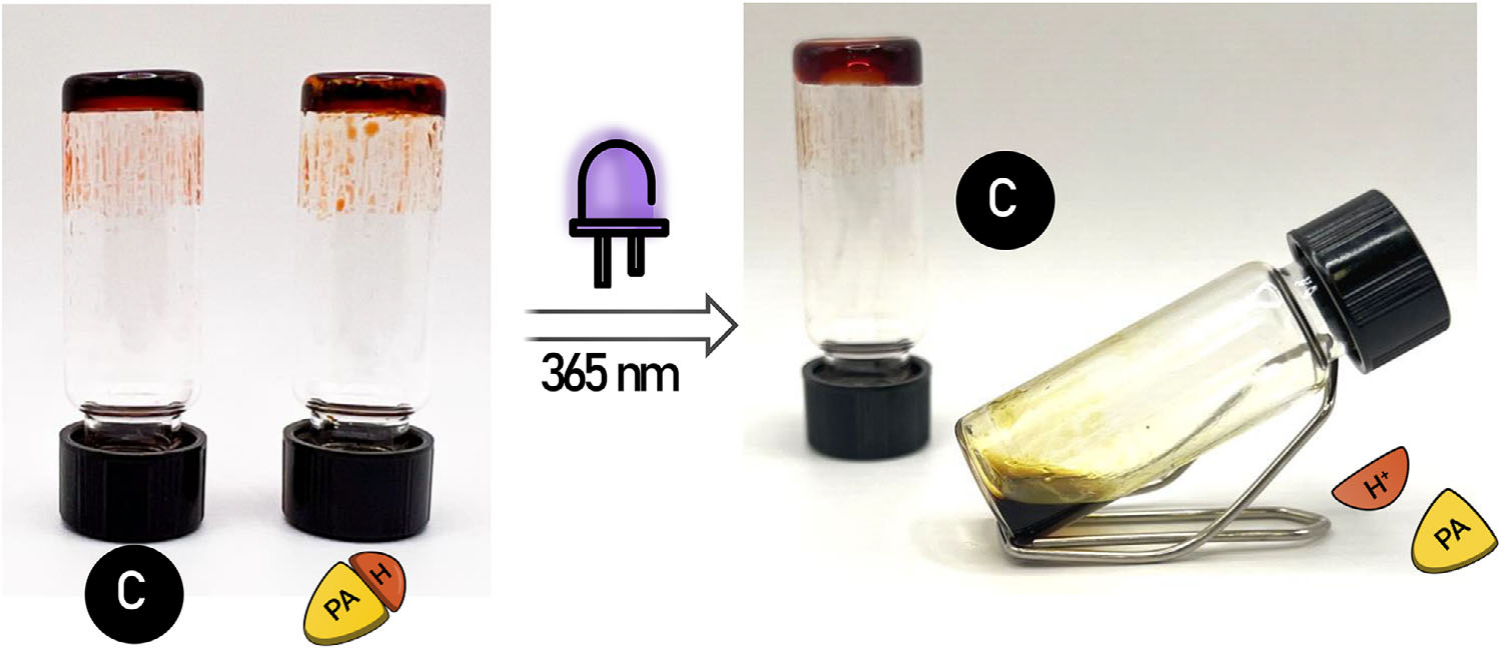
Pictures demonstrating how UV exposure affects **Fe(II)(PBA**‐*co*‐**MBP_5_‐46)‐1:3** (control, C) and **Fe(II)(PBA**‐*co*‐**MBP_5_‐46)‐1:3/MBTT_4_** (PAH) gels in chlorobenzene. Pictures were taken before (left) and after (right) irradiation with UV light (365 nm, 90 mW/cm^2^) for 10 min. The MSP content in the gels was 25 wt.% relative to the mass of chlorobenzene and **Fe(II)(PBA**‐*co*‐**MBP_5_‐46)‐1:3/MBTT_4_** contains 4 eq. of MBTT relative to the ML complexes.

With the HCl‐induced decomplexation confirmed in model studies, we proceeded to MSP organogel systems, which we accessed by adding the PAG MBTT to the copolymer/metal salt mixture used to produce the MSP, which was then swollen with chlorobenzene to ca. 26 mM of MSP complexes at 25 wt% relative to the mass of chlorobenzene, as reported above for the neat MSP organogels. While **Fe(III)(PBA**‐*co*‐**MBP_10_‐56**)‐**1:3** proved to afford the stiffest MSP gels, these materials were somewhat heterogeneous (Figure 4). Furthermore, the high ligand concentration in this material requires a high PAG concentration to dissociate the cross‐links. With the goal of maximizing the mechanical “contrast”, we therefore selected **Fe(II)(PBA**‐*co*‐**MBP_5_‐46)‐1:3** as the basis for these systems. To investigate the range of acid concentrations identified as pertinent in the model studies, gels were prepared with 0.25, 1, and 4 equivalents of MBTT relative to the ML cross‐links (Figure 6; Figure S14). Prior studies indicated that one molecule of MBTT can release up to two protons when irradiated for extended periods with high‐intensity UV irradiation [49]. Their composition is denoted as **Fe(II)(PBA**‐*co*‐**MBP_5_‐46)‐1:3/MBTT_xx_**, where ***xx*** indicates the molar ratio of MBTT relative to ML cross‐links. We conducted oscillatory shear rheology experiments on all MBTT‐loaded organogels and compared their viscoelastic properties to those of the MBTT‐free **Fe(II)(PBA**‐*co*‐**MBP_5_‐46)‐1:3** (Figure S15). All gels exhibit the same linear viscoelastic range, with no observable differences in response to variations in shear amplitude (constant *ω* = 10 rad/s) (Figure S15a). While **Fe(II)(PBA**‐*co*‐**MBP_5_‐46)‐1:3/MBTT_1_** and **Fe(II)(PBA**‐*co*‐**MBP_5_‐46)‐1:3/MBTT_0.25_** also show a frequency‐dependent behavior that closely overlaps with that of the MBTT‐free gels (constant *γ* = 1%), **Fe(II)(PBA**‐*co*‐**MBP_5_‐46)‐1:3/MBTT_4_** displays an increased stiffness at lower deformation frequencies (Figure S15b), indicating that at this high concentration MBTT is not fully dissolved and effectively reinforces the gel.

We first employed a gel inversion test to assess the ability of MBTT to induce dissociation and liquefaction upon UV activation, using **Fe(II)(PBA**‐*co*‐**MBP_5_‐46)‐1:3/MBTT_4_**, the stiffest gel of the series across the entire frequency range. Gratifyingly, a complete gel‐to‐sol transition can be observed when the gel is irradiated with low‐intensity UV light (365 nm, 90 mW cm^−^^2^) for 10 min, whereas the MBTT‐free control gel remains intact under identical conditions (Figure 6). Liquefaction was not observed for **Fe(II)(PBA**‐*co*‐**MBP_5_‐46)‐1:3/MBTT_0.25_**, the gel with the lowest MBTT content, although some softening was noted (Figure S16).

**FIGURE 7:**
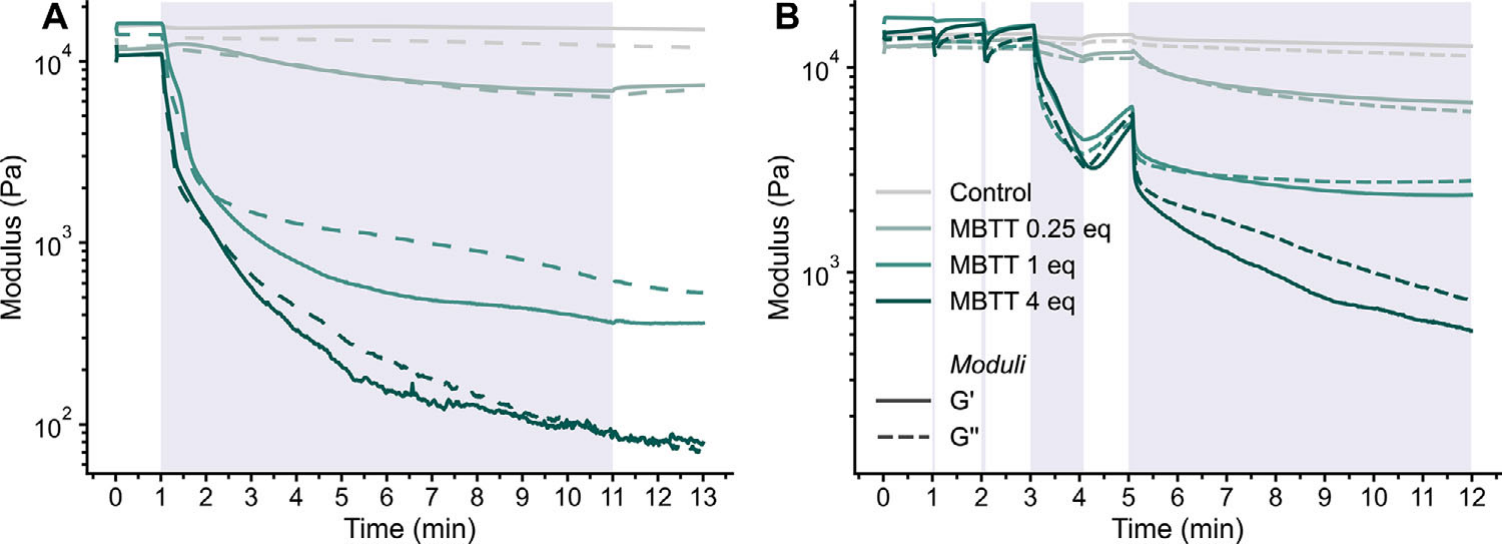
Optorheological experiments conducted on **Fe(II)(PBA‐MBP_5_‐46)‐3:1/MBTT_xx_** MSP gels containing xx equivalents of MBTT relative to the ML complexes in chlorobenzene (25 wt% polymer mass/chlorobenzene mass). (A) After an idle period of 1 min, the UV light was activated (purple shade). (B) After an idle period of 1 min, the UV light was activated for periods of 1 s, 1 s, 1 min, and 7 min (purple shades). The experiments were conducted under nominally isothermal conditions at 25°C with γ = 1%, ω = 10 rad/s, λ = 385 nm, and a power density on the samples of ca. 146 mW/cm^2^.

We then investigated the switching behavior of the entire series of gel systems, i.e., **Fe(II)(PBA**‐*co*‐**MBP_5_‐46)‐1:3/MBTT_xx_** with xx = 0, 0.25, 1, 4, in a quantitative fashion using in situ UV SAOS rheology experiments. Thus, time‐dependent measurements were carried out in which the MSP gels were deformed constantly at *γ* = 1% and *ω* = 10 rad/s and exposed to UV light (385 nm, incident power = 146 mW/cm^2^) while measuring *G*′ and *G*″ (Figure 7a). The gels containing MBTT *all* exhibit a sudden drop in moduli upon UV exposure, with both *G*′ and *G*″ traces displaying rapid decreases. The extent and rate of these changes scale with the MBTT content, with **Fe(II)(PBA**‐*co*‐**MBP_5_‐46)‐1:3/MBTT_4_** experiencing the fastest and the largest softening, followed by **Fe(II)(PBA**‐*co*‐**MBP_5_‐46)‐1:3/MBTT_1_**, and **Fe(II)(PBA**‐*co*‐**MBP_5_‐46)‐1:3/MBTT_0.25_**. In addition to the drop in moduli, the traces all show a crossover of *G*′ and *G*″ traces, which is indicative of a gel‐to‐sol transition. The times to reach these crossover points, 202 s for **Fe(II)(PBA**‐*co*‐**MBP_5_‐46)‐1:3/MBTT_0.25_**, 128 s for **Fe(II)(PBA**‐*co*‐**MBP_5_‐46)‐1:3/MBTT_1_**, and 126 s for **Fe(II)(PBA**‐*co*‐**MBP_5_‐46)‐1:3/MBTT_4_**, are inversely correlated with the MBTT content. The **Fe(II)(PBA**‐*co*‐**MBP_5_‐46)‐1:3** control gel without MBTT exhibits no significant change in moduli, reflecting that the rheological changes observed for the systems are indeed related to the acid‐induced decomplexation of ML cross‐links. Importantly, the experiments demonstrate that the PAG induces rapid gel‐sol transitions in the organogels where it interfaces with light, as observed at the bottom plate rheometer, even with as little as 0.25 equivalents of the MBTT.

Given the fast softening achieved upon continuous UV irradiation, we investigated the possibility of achieving temporal control by irradiating with UV light in shorter time intervals (Figure 7b). The data show that all MBTT‐containing gels exhibit an immediate response to UV light, even with exposure times reduced to 1‐second pulses. Also in these experiments, gels with higher MBTT content display more pronounced modulus changes. As observed in our previous studies on similar gel systems [49, 50], the moduli recover partially after the light source is switched off, but this effect reduces as the overall exposure dose is increased (Figure 7b). We attribute this effect to acid concentration—and hence mechanical—gradients in the gels, which arise from the geometry of the experiment (irradiation from the bottom plate) and the high optical density of the sample, causing an attenuation of the PAG activation along the transverse direction. Once UV irradiation is stopped, the released acid diffuses throughout the sample, the network reorganizes, and its properties become homogeneous. Longer irradiation intervals allow for more effective diffusion during the activation process, less pronounced gradients, and therefore less pronounced recovery of moduli (Figure 7b). To demonstrate the impact of optical density on signal attenuation, **Fe(II)(PBA‐co‐MBP5‐46)‐1:3/MBTT_4_** was loaded with 4 eq. of Rhodamine B (0.1 m) and the sample was irradiated at 365 nm (Figure S17). Intense fluorescence was localized near the light source at the vial base; however, strong attenuation resulted in negligible emission at the top of the sample. This observation confirms the formation of a spatial gradient dictated by the optical path length and optical density.

## 3 Conclusion

In conclusion, we demonstrate that a materials systems approach can address the challenge of balancing mechanical robustness with dynamic responsiveness by incorporating a photoacid generator (PAG) into a stiff MSP gel cross‐linked by metal–ligand (ML) complexes. We demonstrate that the bidentate MBP ligand facilitates the formation of stiff organogels by enhancing their construction through the introduction of a more strongly coordinating Fe^2+^ ion and increasing their overall concentration, while maintaining high sensitivity to acidic changes in the surrounding environment.

## Author Contributions

Christoph Weder, Luca Bertossi, Carina Lin, Marta Oggioni, Davide M. De Luca, and Georges J. M. Formon contributed to the conceptualization of the present study and designed the experiments. Luca Bertossi and Carina Lin carried out the experiments. Luca Bertossi and Carina Lin processed the data under the supervision of Christoph Weder and Georges J. M. Formon. Luca Bertossi and Christoph Weder wrote, and Carina Lin, Davide M. De Luca, Marta Oggioni, and Georges J. M. Formon edited the manuscript. Christoph Weder supervised the project and acquired the funding.

## Funding

The authors gratefully acknowledge financial support from the Swiss National Science Foundation (SNSF) through a grant to C.W. (200020_207796) and the National Center of Competence in Research (NCCR) Bio‐Inspired Materials, a research instrument of the SNSF, under grant number 205603.

## Conflicts of Interest

The authors declare no conflicts of interest.

## Supporting information




**Supporting File**: marc70267‐sup‐0001‐SuppMat.docx.

## Data Availability

The data that support the findings of this study are openly available in Zenodo at https://zenodo.org/10.5281/zenodo.18672028.

## References

[1] D. Zhou , M. Gao , F. Chen , et al., “Stimuli‐Responsive Materials With Programmable Shape: Programming Strategies and Applications,” European Polymer Journal 236 (2025): 114101.

[2] M. Wei , Y. Gao , X. Li , and M. J. Serpe , “Stimuli‐Responsive Polymers and Their Applications,” Polymer Chemistry 8, no. 1 (2017): 127–143.

[3] M. A. C. Stuart , W. T. S. Huck , J. Genzer , et al., “Emerging Applications of Stimuli‐Responsive Polymer Materials,” Nature Materials 9, no. 2 (2010): 101–113. 20094081 10.1038/nmat2614

[4] L. Zhai , “Stimuli‐Responsive Polymer Films,” Chemical Society Reviews 42, no. 17 (2013): 7148–7160. 23749141 10.1039/c3cs60023h

[5] S. Mura , J. Nicolas , and P. Couvreur , “Stimuli‐Responsive Nanocarriers for Drug Delivery,” Nature Materials 12, no. 11 (2013): 991–1003. 24150417 10.1038/nmat3776

[6] K. M. Herbert , S. Schrettl , S. J. Rowan , and C. Weder , “50th Anniversary Perspective: Solid‐State Multistimuli, Multiresponsive Polymeric Materials,” Macromolecules 50, no. 22 (2017): 8845–8870.

[7] C. J. Kloxin and C. N. Bowman , “Covalent Adaptable Networks: Smart, Reconfigurable and Responsive Network Systems,” Chemical Society Reviews 42, no. 17 (2013): 7161–7173. 23579959 10.1039/c3cs60046g

[8] F. Xu and B. L. Feringa , “Photoresponsive Supramolecular Polymers: From Light‐Controlled Small Molecules to Smart Materials,” Advanced Materials 35, no. 10 (2023): 2204413. 10.1002/adma.202204413 36239270

[9] D. Kuckling and M. W. Urban , “Synthetic and Physicochemical Aspects of Advanced Stimuli‐Responsive Polymers,” Handbook of Stimuli‐Responsive Materials (Wiley, 2011): 1–26.

[10] B. L. Feringa , “The Art of Building Small: From Molecular Switches to Molecular Motors,” The Journal of Organic Chemistry 72, no. 18 (2007): 6635–6652. 17629332 10.1021/jo070394d

[11] T. F. A. de Greef and E. W. Meijer , “Supramolecular Polymers,” Nature 453, no. 7192 (2008): 171–173. 18464733 10.1038/453171a

[12] A. J. Wilson , “Non‐Covalent Polymer Assembly Using Arrays of Hydrogen‐Bonds,” Soft Matter 3, no. 4 (2007): 409–425. 32900059 10.1039/b612566b

[13] A. J. McConnell , C. S. Wood , P. P. Neelakandan , and J. R. Nitschke , “Stimuli‐Responsive Metal–Ligand Assemblies,” Chemical Reviews 115, no. 15 (2015): 7729–7793. 25880789 10.1021/cr500632f

[14] O. J. G. M. Goor , S. I. S. Hendrikse , P. Y. W. Dankers , and E. W. Meijer , “From Supramolecular Polymers to Multi‐Component Biomaterials,” Chemical Society Reviews 46, no. 21 (2017): 6621–6637. 28991958 10.1039/c7cs00564d

[15] T. D. Clemons and S. I. Stupp , “Design of Materials With Supramolecular Polymers,” Progress in Polymer Science 111 (2020): 101310. 33082608 10.1016/j.progpolymsci.2020.101310 PMC7560124

[16] C. Heinzmann , C. Weder , and L. M. de Espinosa , “Supramolecular Polymer Adhesives: Advanced Materials Inspired by Nature,” Chemical Society Reviews 45, no. 2 (2016): 342–358. 26203784 10.1039/c5cs00477b

[17] L. Yang , X. Tan , Z. Wang , and X. Zhang , “Supramolecular Polymers: Historical Development, Preparation, Characterization, and Functions,” Chemical Reviews 115, no. 15 (2015): 7196–7239. 25768045 10.1021/cr500633b

[18] Y. Liu , L. Wang , L. Zhao , Y. Zhang , Z.‐T. Li , and F. Huang , “Multiple Hydrogen Bonding Driven Supramolecular Architectures and Their Biomedical Applications,” Chemical Society Reviews 53, no. 3 (2024): 1592–1623. 38167687 10.1039/d3cs00705g

[19] M. Anthamatten , “Hydrogen Bonding in Supramolecular Polymer Networks: Glasses, Melts, and Elastomers,” in Supramolecular Polymer Networks and Gels, ed. S. Seiffert , (Springer International Publishing: Cham, 2015): 47–99.

[20] Y. Qian , F. Dong , S. Wang , Y. Jiang , X. Xu , and H. Liu , “Ultrarobust, Stretchable, and Highly Elastic Supramolecular Elastomer With Hydrogen‐Bond Interactions via sp^2^ Hybridized Boron‐Urethane Bonds,” Angewandte Chemie International Edition 64, no. 21 (2025): 202421099. 10.1002/anie.202421099 40063009

[21] L. Brunsveld , B. J. B. Folmer , E. W. Meijer , and R. P. Sijbesma , “Supramolecular Polymers,” Chemical Reviews 101, no. 12 (2001): 4071–4098. 11740927 10.1021/cr990125q

[22] M. Burnworth , L. Tang , J. R. Kumpfer , et al., “Optically Healable Supramolecular Polymers,” Nature 472, no. 7343 (2011): 334–337. 21512571 10.1038/nature09963

[23] S. J. Rowan and J. B. Beck , “Metal–Ligand Induced Supramolecular Polymerization: A Route to Responsive Materials,” Faraday Discuss 128, no. 0 (2005): 43–53. 15658766 10.1039/b403135k

[24] A. Winter and U. S. Schubert , “Synthesis and Characterization of Metallo‐Supramolecular Polymers,” Chemical Society Reviews 45, no. 19 (2016): 5311–5357. 27218823 10.1039/c6cs00182c

[25] L. Casimiro , F. Volatron , G. Boivin , et al., “Multifunctional Supramolecular Gels With Strong Mechanical Properties Formed by Self‐Assembly of Polyoxometalate‐Based Coordination Polymers,” JACS Au 4, no. 12 (2024): 4948–4956. 39735907 10.1021/jacsau.4c00981 PMC11672139

[26] L. Chen , X. Sheng , G. Li , and F. Huang , “Mechanically Interlocked Polymers Based on Rotaxanes,” Chemical Society Reviews 51, no. 16 (2022): 7046–7065. 35852571 10.1039/d2cs00202g

[27] K. Kato , K. Ito , and T. Hoshino , “Strain‐Induced Orientation of Host Rings That Determines the Sliding of Guest Polymers and Plasticity of Glassy Polyrotaxane,” ACS Macro Letters 13, no. 8 (2024): 1094–1098. 39121179 10.1021/acsmacrolett.4c00369 PMC11340018

[28] A. Skandalis , M. A. Ayer , and C. Weder , “Bioinspired Adhesives With Debonding‐On‐Demand Capability,” ACS Macro Letters 14, no. 4 (2025): 420–427. 40099815 10.1021/acsmacrolett.5c00035

[29] T. Aida , E. W. Meijer , and S. I. Stupp , “Functional Supramolecular Polymers,” Science 335, no. 6070 (2012): 813–817. 22344437 10.1126/science.1205962 PMC3291483

[30] Y. Gu , E. A. Alt , H. Wang , X. Li , A. P. Willard , and J. A. Johnson , “Photoswitching Topology in Polymer Networks With Metal–Organic Cages as Crosslinks,” Nature 560, no. 7716 (2018): 65–69. 30022167 10.1038/s41586-018-0339-0

[31] N. J. Oldenhuis , K. P. Qin , S. Wang , et al., “Photoswitchable Sol–Gel Transitions and Catalysis Mediated by Polymer Networks With Coumarin‐Decorated Cu_24_L_24_ Metal–Organic Cages as Junctions,” Angewandte Chemie International Edition 59, no. 7 (2020): 2784–2792. 31742840 10.1002/anie.201913297 PMC7187918

[32] L. N. Neumann , C. Calvino , Y. C. Simon , S. Schrettl , and C. Weder , “Solid‐State Sensors Based on Eu^3+^‐Containing Supramolecular Polymers With Luminescence Colour Switching Capability,” Dalton Transactions 47, no. 40 (2018): 14184–14188. 29995055 10.1039/c8dt01580e

[33] Y.‐Q. Jiang , K. Wu , Q. Zhang , et al., “A Dual‐Responsive Hyperbranched Supramolecular Polymer Constructed by Cooperative Host–Guest Recognition and Hydrogen‐Bond Interactions,” Chemical Communications 54, no. 98 (2018): 13821–13824. 30462109 10.1039/c8cc08226j

[34] G. R. Whittell , M. D. Hager , U. S. Schubert , and I. Manners , “Functional Soft Materials From Metallopolymers and Metallosupramolecular Polymers,” Nature Materials 10, no. 3 (2011): 176–188. 21336298 10.1038/nmat2966

[35] H. Chen , C. Wang , H. Wu , et al., “Host‐Guest‐Induced Electronic State Triggers Two‐Electron Oxygen Reduction Electrocatalysis,” Nature Communications 15, no. 1 (2024): 9222. 10.1038/s41467-024-53714-3 PMC11512016 39455580

[36] S. Selmani , E. Schwartz , J. T. Mulvey , et al., “Electrically Fueled Active Supramolecular Materials,” Journal of the American Chemical Society 144, no. 17 (2022): 7844–7851. 35446034 10.1021/jacs.2c01884

[37] C. W. Zhang , X. P. Hao , W. Zou , et al., “Supramolecular Hydrogel Actuators With Reprogrammable Magnetic Orientation by Locally Mediated Viscoelasticity and Pinning Force,” Science Advances 11, no. 26 (2025): adw0500. 10.1126/sciadv.adw0500 PMC12204151 40577469

[38] D. W. R. Balkenende , S. Coulibaly , S. Balog , Y. C. Simon , G. L. Fiore , and C. Weder , “Mechanochemistry With Metallosupramolecular Polymers,” Journal of the American Chemical Society 136, no. 29 (2014): 10493–10498. 24972163 10.1021/ja5051633

[39] E. M. Lloyd , J. R. Vakil , Y. Yao , N. R. Sottos , and S. L. Craig , “Covalent Mechanochemistry and Contemporary Polymer Network Chemistry: A Marriage in the Making,” Journal of the American Chemical Society 145, no. 2 (2023): 751–768. 36599076 10.1021/jacs.2c09623

[40] C. Mak‐iad , L. Bertossi , G. J. M. Formon , and C. Weder , “Healable Glassy Metallosupramolecular Polymers,” ACS Macro Letters 14 (2025): 996–1003. 40587150 10.1021/acsmacrolett.5c00317 PMC12269065

[41] S.‐M. Yang , S. Zhou , and J.‐Y. Yuan , “Self‐Healing Elastomers and Coatings via Metal Coordination Bonds,” Chemistry—A European Journal 31, no. 31 (2025): 202404038. 10.1002/chem.202404038 39757123

[42] E. Borré , J.‐F. Stumbé , S. Bellemin‐Laponnaz , and M. Mauro , “Light‐Powered Self‐Healable Metallosupramolecular Soft Actuators,” Angewandte Chemie International Edition 55, no. 4 (2016): 1313–1317. 26679405 10.1002/anie.201508241

[43] C. Li , A. Iscen , H. Sai , et al., “Supramolecular–Covalent Hybrid Polymers for Light‐Activated Mechanical Actuation,” Nature Materials 19, no. 8 (2020): 900–909. 32572204 10.1038/s41563-020-0707-7

[44] A. H. Gelebart , J. Mulder , M. Varga , et al., “Making Waves in a Photoactive Polymer Film,” Nature 546, no. 7660 (2017): 632–636. 28658225 10.1038/nature22987 PMC5495175

[45] Y. Deng , L. Liu , H.‐X. Luo , et al., “Supramolecular Chemical Recycling of Dynamic Polymers,” Nature Nanotechnology 20 (2025): 1805–1812. 10.1038/s41565-025-02041-9 41193862

[46] D. J. Kiebala , A. Dodero , C. Weder , and S. Schrettl , “Optical Monitoring of Supramolecular Interactions in Polymers,” Angewandte Chemie International Edition 63, no. 36 (2024): 202405922. 10.1002/anie.202405922 38860450

[47] P. K. Hashim , J. Bergueiro , E. W. Meijer , and T. Aida , “Supramolecular Polymerization: A Conceptual Expansion for Innovative Materials,” Progress in Polymer Science 105 (2020): 101250.

[48] B. T. Worrell , M. K. McBride , G. B. Lyon , et al., “Bistable and Photoswitchable States of Matter,” Nature Communications 9, no. 1 (2018): 2804. 10.1038/s41467-018-05300-7 PMC6052001 30022053

[49] L. Bertossi , M. Oggioni , G. J. M. Formon , and C. Weder , “Light‐Triggered Switching of Metallosupramolecular Polymer Systems,” ACS Macro Letters 14, no. 6 (2025): 765–772. 40393647 10.1021/acsmacrolett.5c00205 PMC12177937

[50] M. Oggioni , L. Bertossi , D. J. Kiebala , et al., “Photoacid‐Induced Supramolecular Network Disassembly: A Systems Approach to Stimuli‐Responsive Polymers,” Angewandte Chemie International Edition 137 (2025): 202506981. 10.1002/anie.202506981 40401486

[51] F. Marx , M. Beccard , A. Ianiro , et al., “Structure and Properties of Metallosupramolecular Polymers With a Nitrogen‐Based Bidentate Ligand,” Macromolecules 56, no. 18 (2023): 7320–7331. 37781212 10.1021/acs.macromol.3c00503 PMC10537925

[52] A. Lenoch , M. Schönhoff , and C. Cramer , “Relaxing, Fast and Slow: Linear Viscoelasticity and Dynamics in Mixed Cross‐Linker Metallosupramolecular Networks,” Macromolecules 57, no. 24 (2024): 11507–11519.

[53] Y. Zhu , W. Zheng , W. Wang , and H.‐B. Yang , “When Polymerization Meets Coordination‐Driven Self‐Assembly: Metallo‐Supramolecular Polymers Based on Supramolecular Coordination Complexes,” Chemical Society Reviews 50, no. 13 (2021): 7395–7417. 34018496 10.1039/d0cs00654h

[54] S. Xu , J. E. T. Smith , and J. M. Weber , “UV Spectra of Tris(2,2′‐bipyridine)–M(II) Complex Ions in Vacuo (M = Mn, Fe, Co, Ni, Cu, Zn),” Inorganic Chemistry 55, no. 22 (2016): 11937–11943. 27797197 10.1021/acs.inorgchem.6b02054

[55] G. L. Miessler , P. J. Fischer , and D. A. Tarr , Inorganic Chemistry 5, (2014) 201–209.

[56] A. K. Miller , Z. Li , K. A. Streletzky , A. M. Jamieson , and S. J. Rowan , “Redox‐Induced Polymerisation/Depolymerisation of Metallo‐Supramolecular Polymers,” Polymer Chemistry 3, no. 11 (2012): 3132–3138.

[57] C. Piguet , B. Bocquet , E. Müller , and A. F. Williams , “Models for Copper‐Dioxygen Complexes: The Chemistry of Copper(II) With Some Planar Tridentate Nitrogen Ligands,” Helvetica Chimica Acta 72, no. 2 (1989): 323–337.

[58] Y. Xu , J. Fei , G. Li , et al., “Enhanced Photophosphorylation of a Chloroplast‐Entrapping Long‐Lived Photoacid,” Angewandte Chemie International Edition 56, no. 42 (2017): 12903–12907. 28834071 10.1002/anie.201706368

[59] A. M. May and J. L. Dempsey , “A New Era of LMCT: Leveraging Ligand‐To‐Metal Charge Transfer Excited States for Photochemical Reactions,” Chemical Science 15, no. 18 (2024): 6661–6678. 38725519 10.1039/d3sc05268k PMC11079626

[60] R. A. Palmer , “2,2'‐Bipyridine Complexes. I. Polarized Crystal Spectra of Tris (2,2'‐bipyridine)Copper(II), ‐nickel(II), ‐cobalt(II), ‐iron(II), and ‐ruthenium(II),” Inorganic Chemistry 5, no. 5 (1966): 864–878.

[61] E. Garribba , G. Micera , D. Sanna , and L. Strinna‐Erre , “The Cu(II)‐2,2′‐bipyridine System Revisited,” Inorganica Chimica Acta 299, no. 2 (2000): 253–261.

[62] Z. Wang , W. Fan , R. Tong , X. Lu , and H. Xia , “Thermal‐Healable and Shape Memory Metallosupramolecular Poly(n‐butyl acrylate‐co‐methyl methacrylate) Materials,” RSC Advances 4, no. 49 (2014): 25486–25493.

[63] A. C. Jackson , F. L. Beyer , S. C. Price , B. C. Rinderspacher , and R. H. Lambeth , “Role of Metal–Ligand Bond Strength and Phase Separation on the Mechanical Properties of Metallopolymer Films,” Macromolecules 46, no. 14 (2013): 5416–5422.

[64] L. N. Neumann , D. A. Urban , P. Lemal , et al., “Preparation of Metallosupramolecular Single‐Chain Polymeric Nanoparticles and Their Characterization by Taylor Dispersion,” Polymer Chemistry 11, no. 2 (2020): 586–592.

[65] S. Laquerbe , J. Es Sayed , C. Lorthioir , et al., “Supramolecular Crosslinked Hydrogels: Similarities and Differences With Chemically Crosslinked Hydrogels,” Macromolecules 56, no. 18 (2023): 7406–7418.

