# Supplementary material for: Photoresponsive Metallo‐Supramolecular Systems Constructed From a Bidentate Ligand
Source: Macromol Rapid Commun. 2026 Mar 22;47(11):e70267. doi: 10.1002/marc.70267 (PMC13238319; doi:10.1002/marc.70267)
Supplement: Supplementary file 1 — Supporting File: marc70267‐sup‐0001‐SuppMat.docx. [file MARC-47-e70267-s001.docx]

**Photoresponsive *metallo*-supramolecular systems constructed from a bidentate ligand**

**Electronic Supplementary Information**

Luca Bertossi, Carina Lin, Davide M. De Luca, Marta Oggioni, Georges J.M. Formon*, Christoph Weder^*^

Adolphe Merkle Institute, Polymer Chemistry and Materials, University of Fribourg, Chemin des Verdiers 4,1700 Fribourg, Switzerland

Table of Contents

[**Supplementary Figures S1-S17 and Table S1** 1](#_Toc222418941)

[**Materials and Instrumentation** 10](#_Toc222418942)

[**Synthetic Methods and Characterization** 11](#_Toc222418943)

[**NMR Characterization** 15](#_Toc222418944)

[**References** 22](#_Toc222418945)

**Supplementary Figures S1-S17 and Table S1**


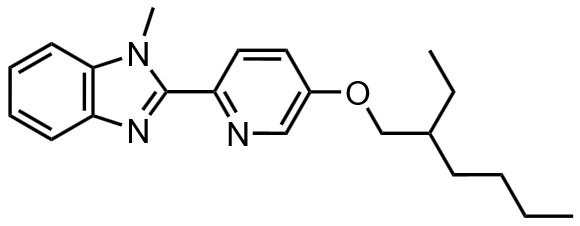


**Figure S1**. Chemical structure of **EH-MBP** used in model studies.


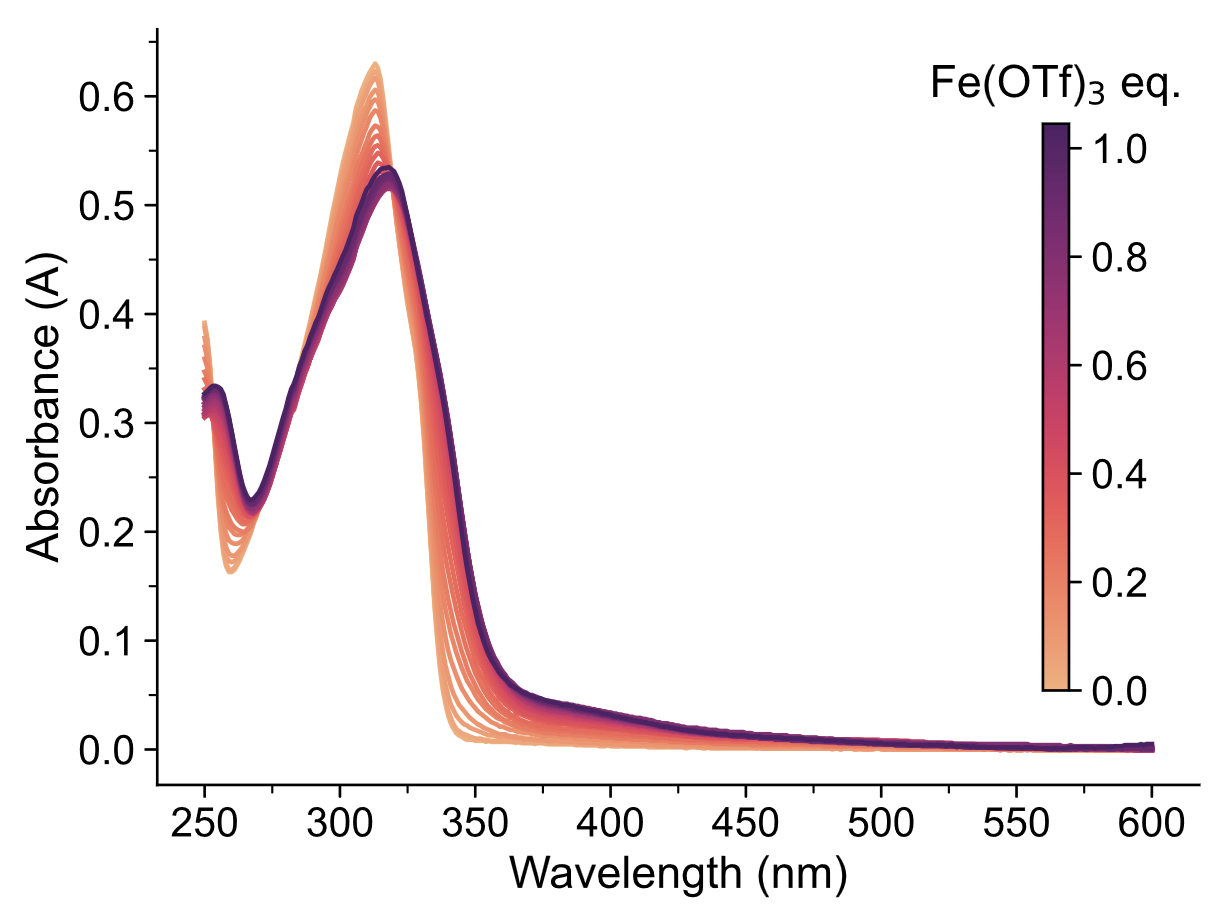

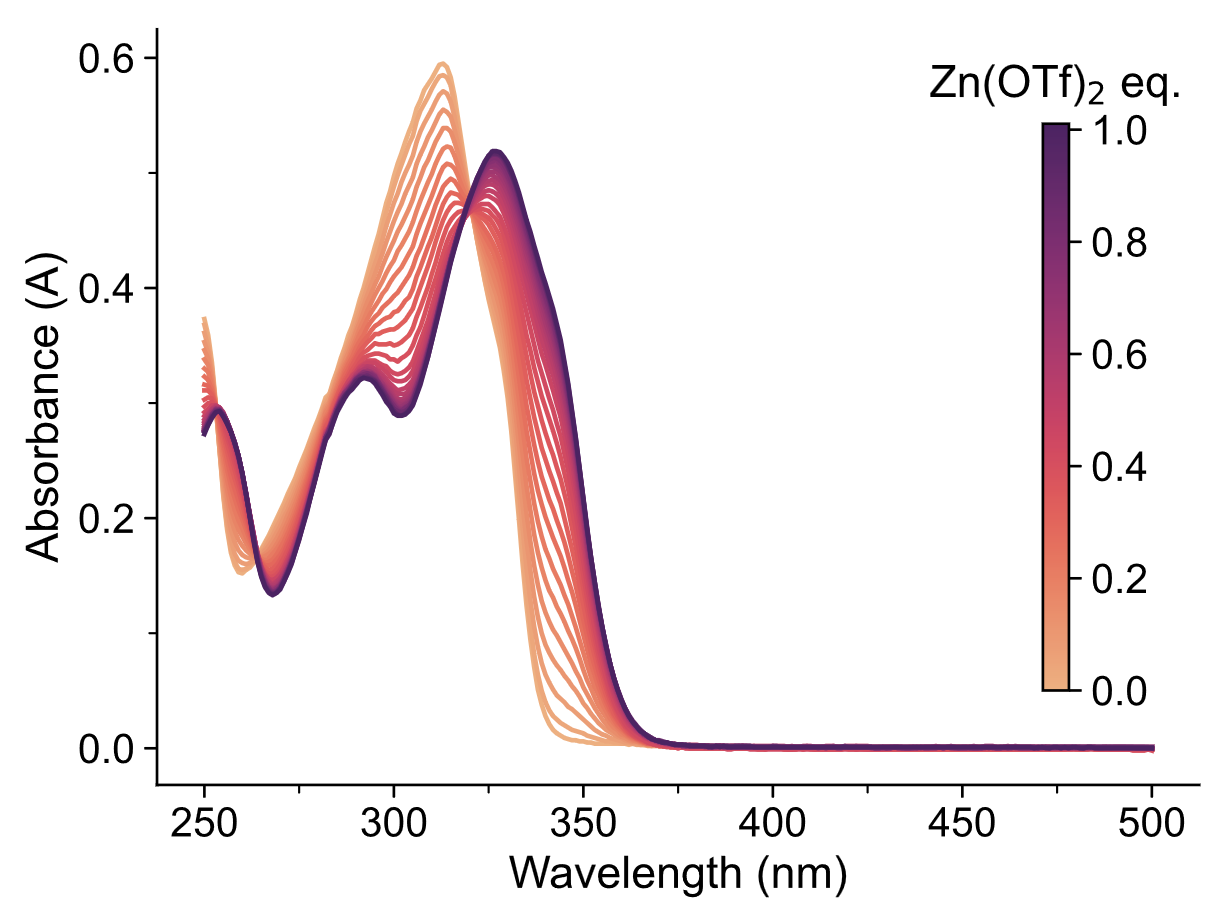

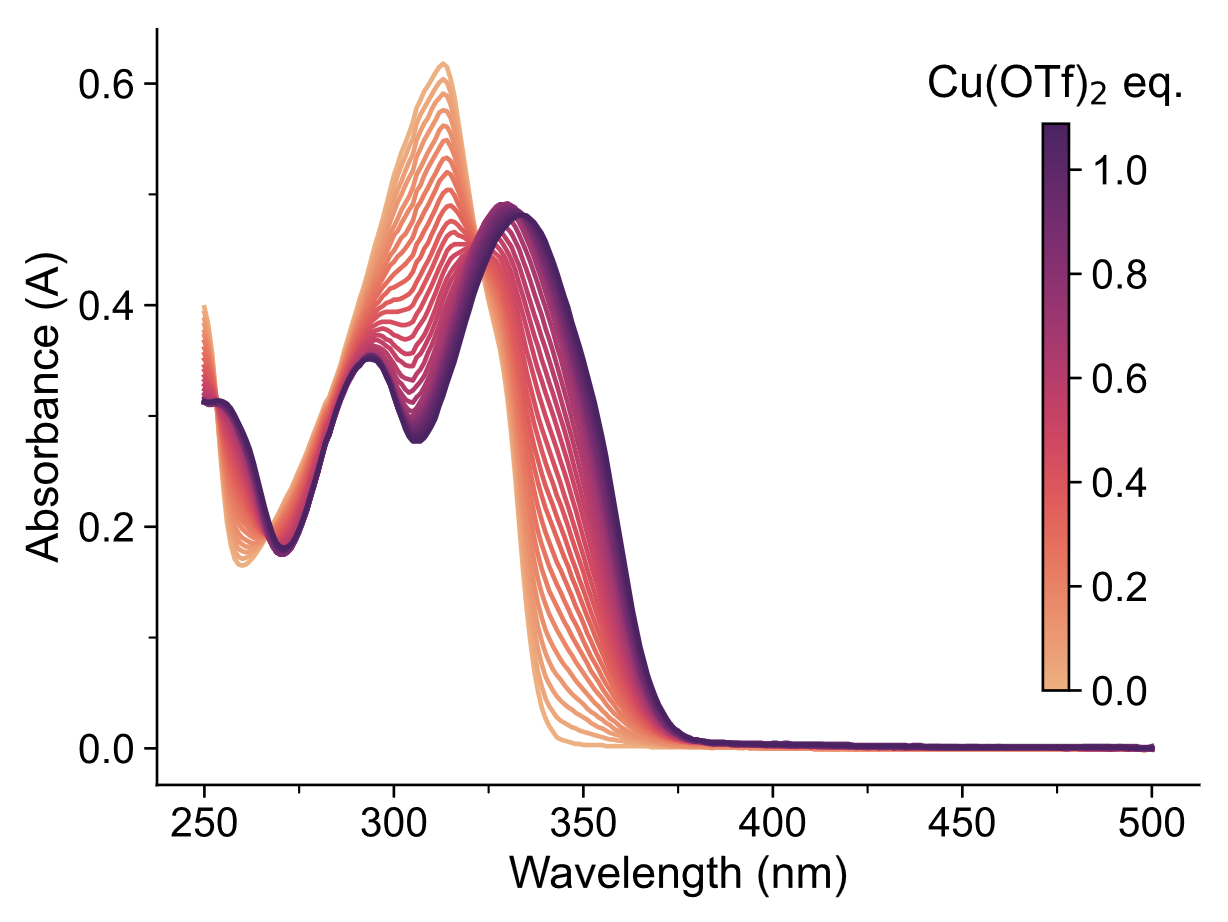


**A**

**B**

**C**

**Figure S2**. The absorption spectra attained following titrations of **EH-MBP** (c=24 µM) with aliquots of (A) Zn(OTf)_2_ (c=0.61 mM), (B) Cu(OTf)_2_ (c=0.57 mM), and (C) Fe(OTf)_3_ (c=0.68 mM) in MeCN at lower concentrations.


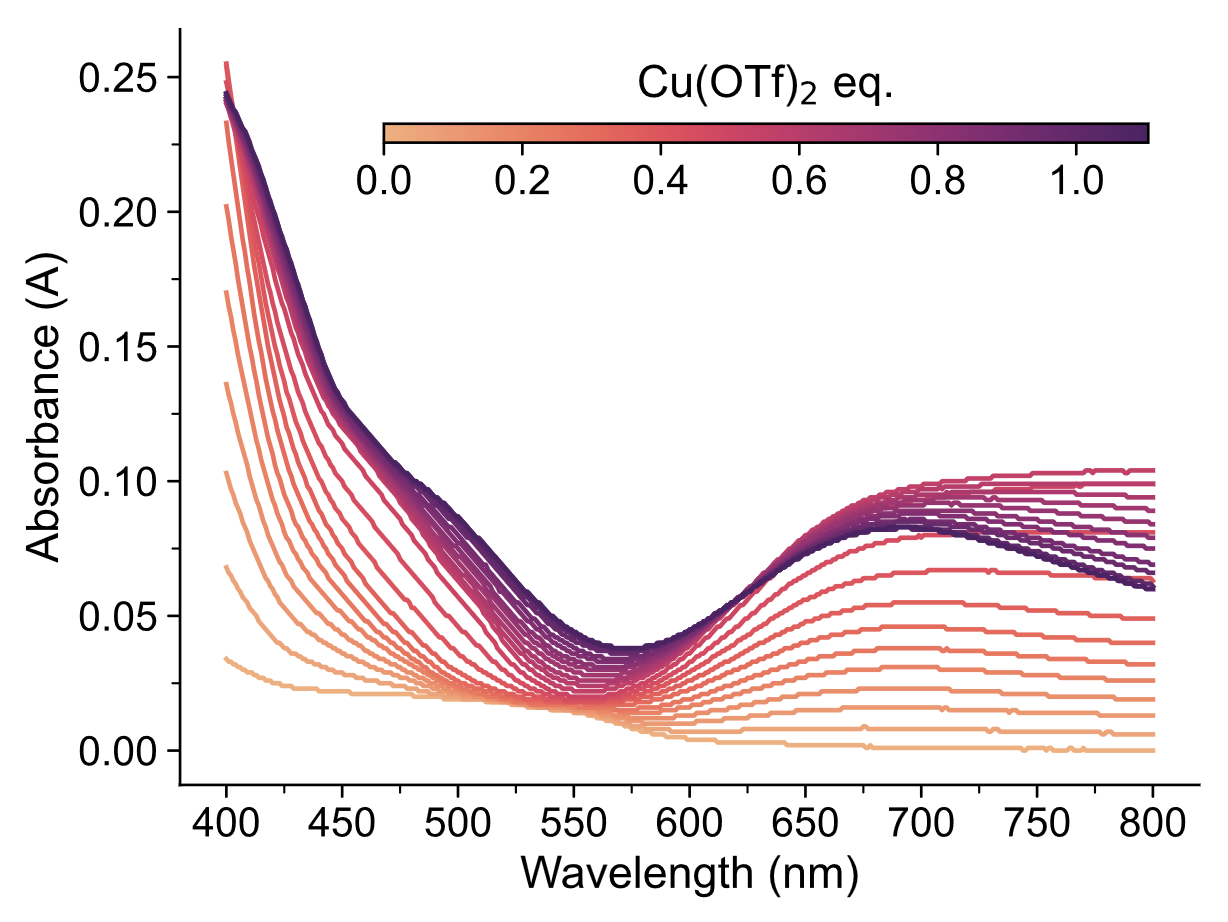

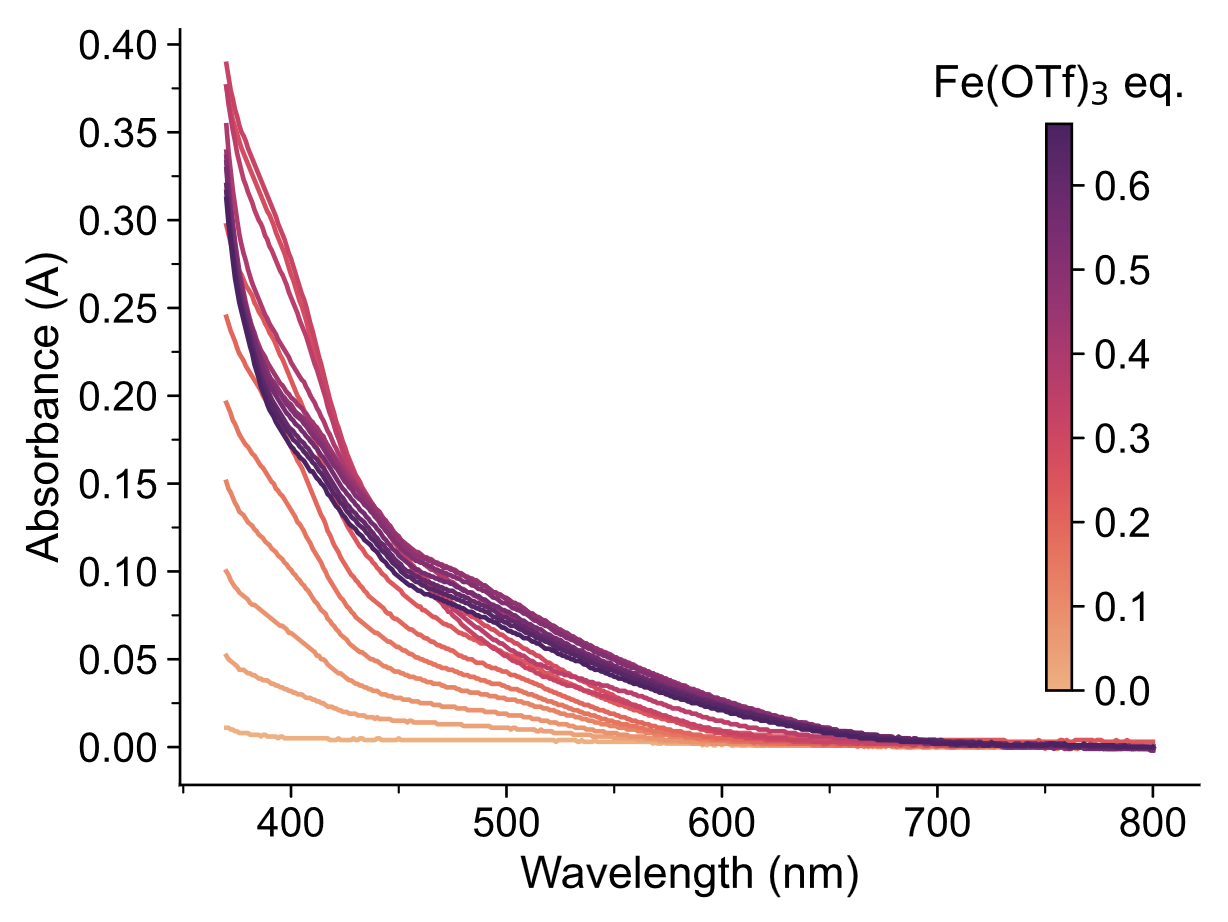


**A**

**B**

**Figure S3**. UV-Vis absorption spectra attained following titrations of (A) **EH-MBP** (c=1.5 mM) with aliquots of Cu(OTf)_2_ (c=23 mM), and (B) **EH-MBP** (*c*=0.24 mM) with aliquots of Fe(OTf)_3_ (c=6.3 mM) in MeCN at higher concentrations.


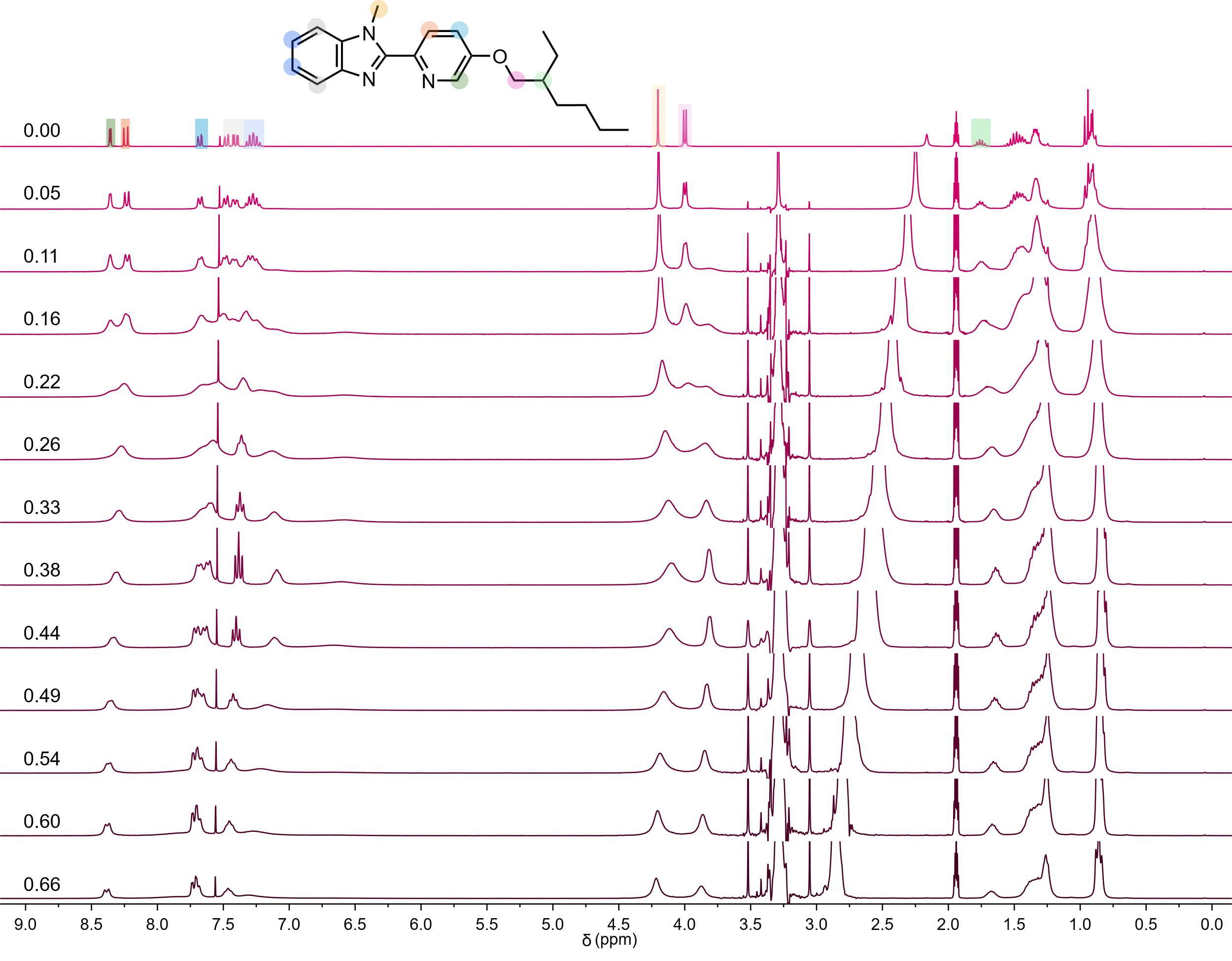


**Figure S4**. Titration of a solution of **EH-MBP** (c=44 mM) in CD_3_CN:CDCl_3_ (8:2) with Zn(OTf)_2_ (c=0.28 M) dissolved in MeOH monitored using ^1^H-NMR spectroscopy after 5 µL additions. A stack of the ^1^H-NMR spectra of the different ratios of [Zn(OTf)_2_]:[**EH-MBP**] is annotated on the left of each spectrum.


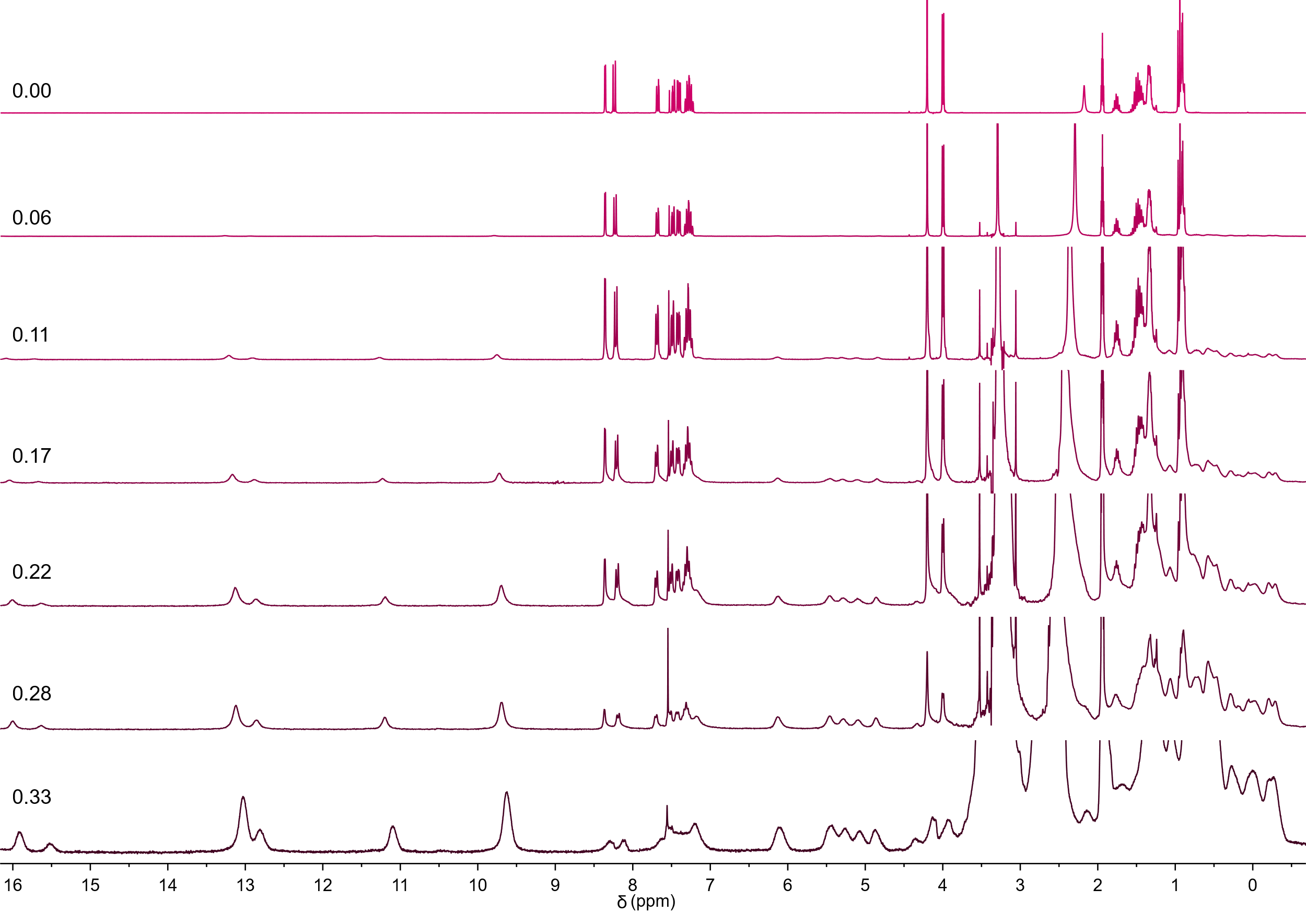


**Figure S5**. Titration of a solution of **EH-MBP** (*c*=45 mM) in CD_3_CN:CDCl_3_ (8:2) with Fe(OTf)_2_ (*c*=0.24 M) dissolved in MeOH monitored using ^1^H-NMR spectroscopy after 6.4 µL additions. A stack of the ^1^H-NMR spectra of the different ratios of [Fe(OTf)_2_]:[**EH-MBP**] is annotated on the left of each spectrum.


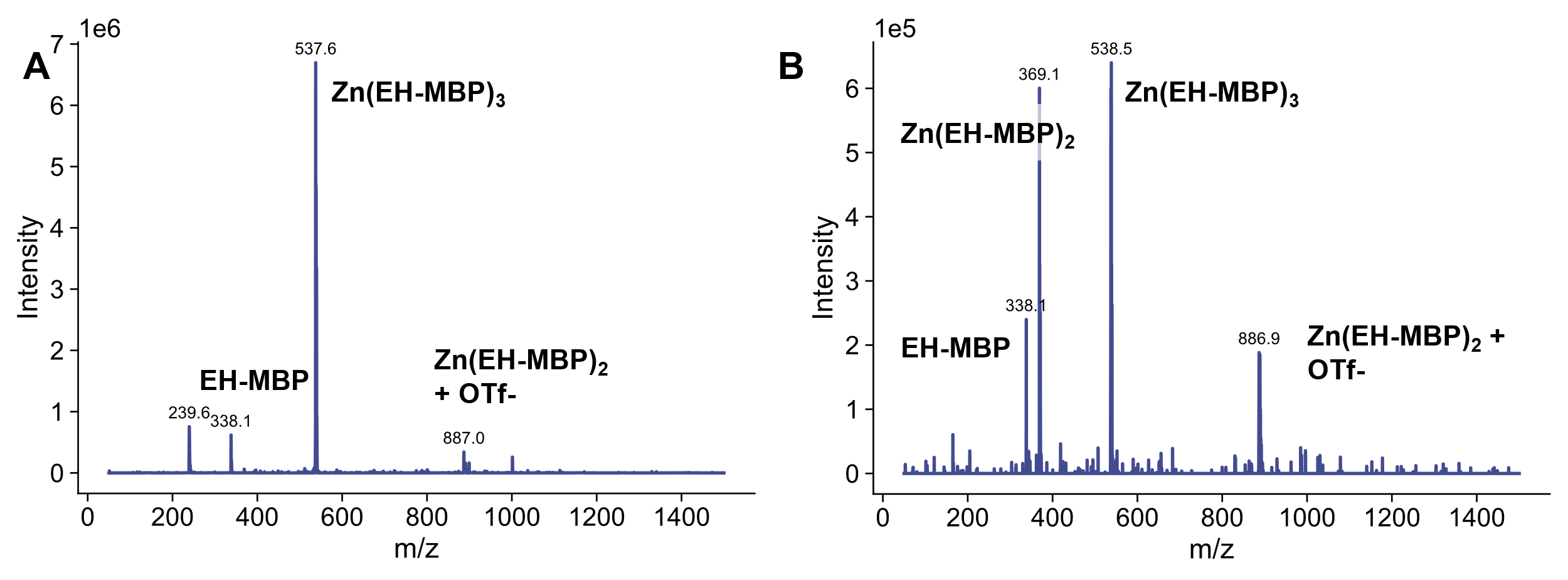


**Figure S6**. Mass spectra of solutions (*c*=±3.0 mM) containing Zn(OTf)_2_ and EH-MBP in a ratio of [Zn(OTf)_2_]:[EH-MBP] = 0.33 (A) and [Zn(OTf)_2_]:[EH-MBP] = 0.5 (B) in MeCN:CHCl_3_ (9:1). Peaks are annotated with the compounds they represent.


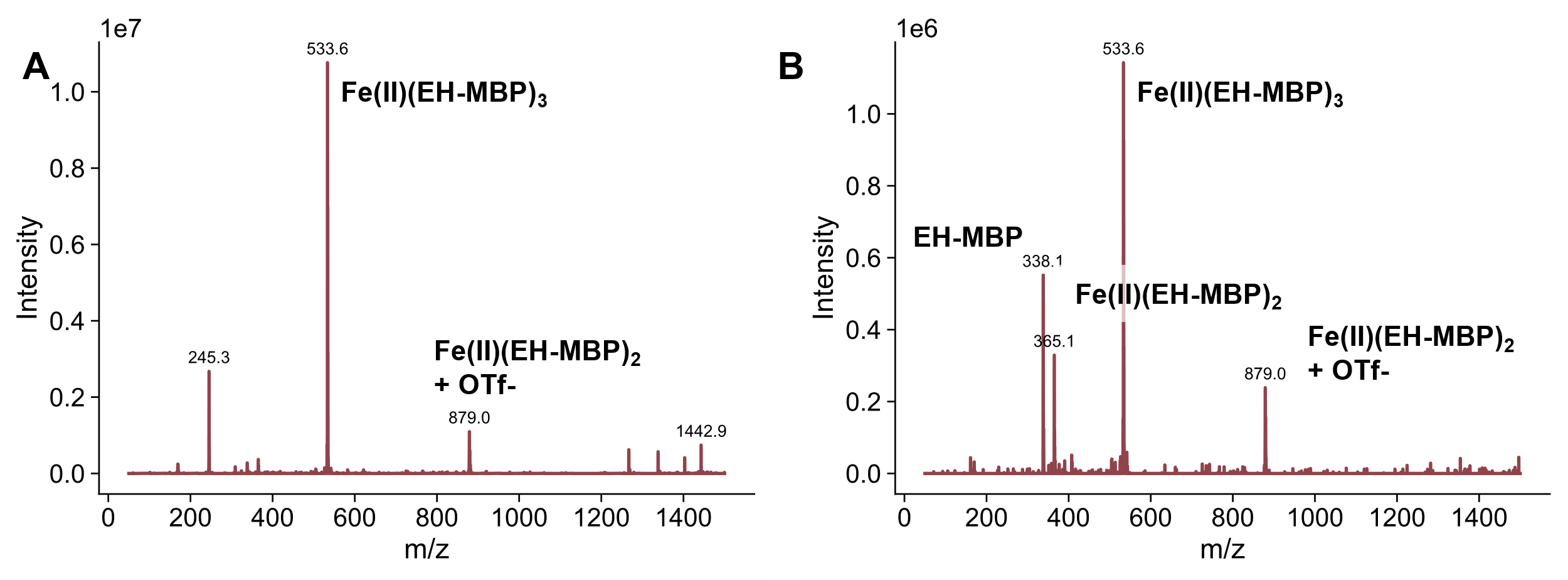


**Figure S7**. Mass spectra of solutions (*c*=±3.0 mM) containing Fe(OTf)_2_ and EH-MBP in a ratio of [Fe(OTf)_2_]:[EH-MBP] = 0.33 (A) and [Fe(OTf)_2_]:[EH-MBP] = 0.5 (B) in MeCN:CHCl_3_ (9:1). Peaks are annotated with the compounds they represent.


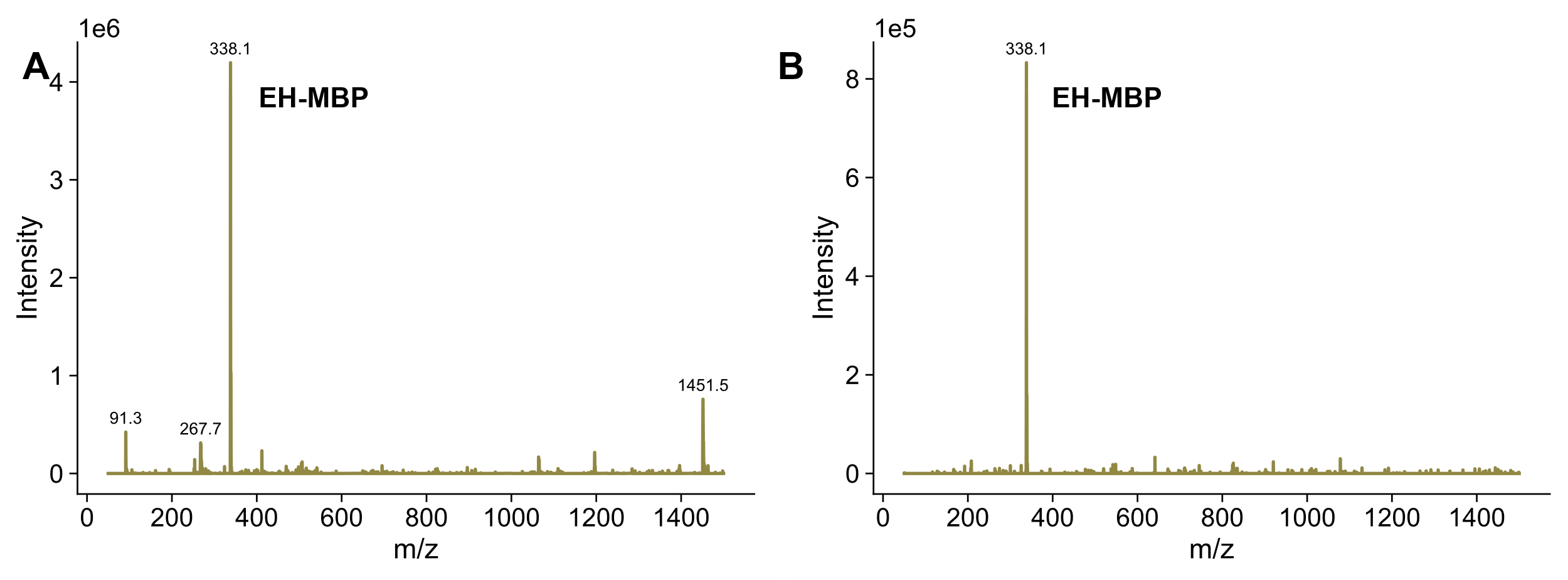


**Figure S8**. Mass spectra of solutions (*c*=±3.0 mM) containing Fe(OTf)_3_ and EH-MBP in a ratio of [Fe(OTf)_3_]:[EH-MBP] = 0.33 (A) and [Fe(OTf)_3_]:[EH-MBP] = 0.5 (B) in MeCN:CHCl_3_ (9:1). Peaks are annotated with the compounds they represent.


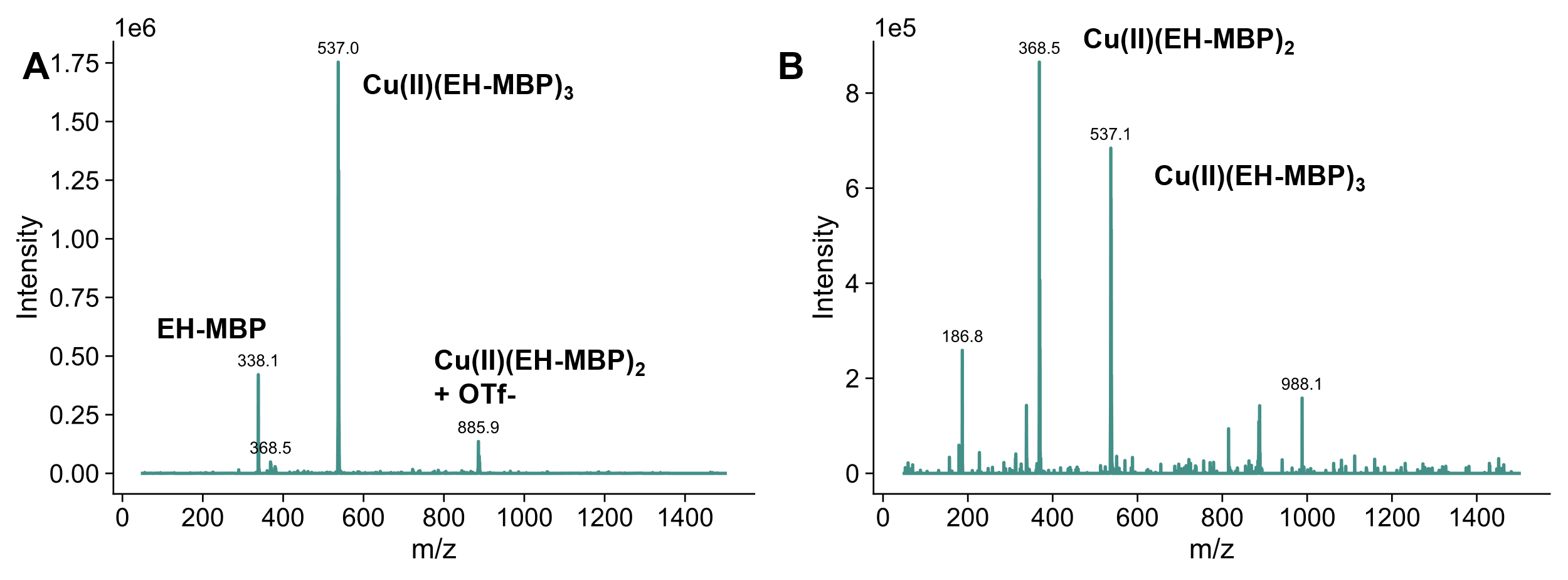


**Figure S9**. Mass spectra of solutions (*c*=±3.0 mM) containing Cu(OTf)_2_ and EH-MBP in a ratio of [Cu(OTf)_2_]:[EH-MBP] = 0.33 (A) and [Cu(OTf)_2_]:[EH-MBP] = 0.5 (B) in MeCN:CHCl_3_ (9:1). Peaks are annotated with the compounds they represent.


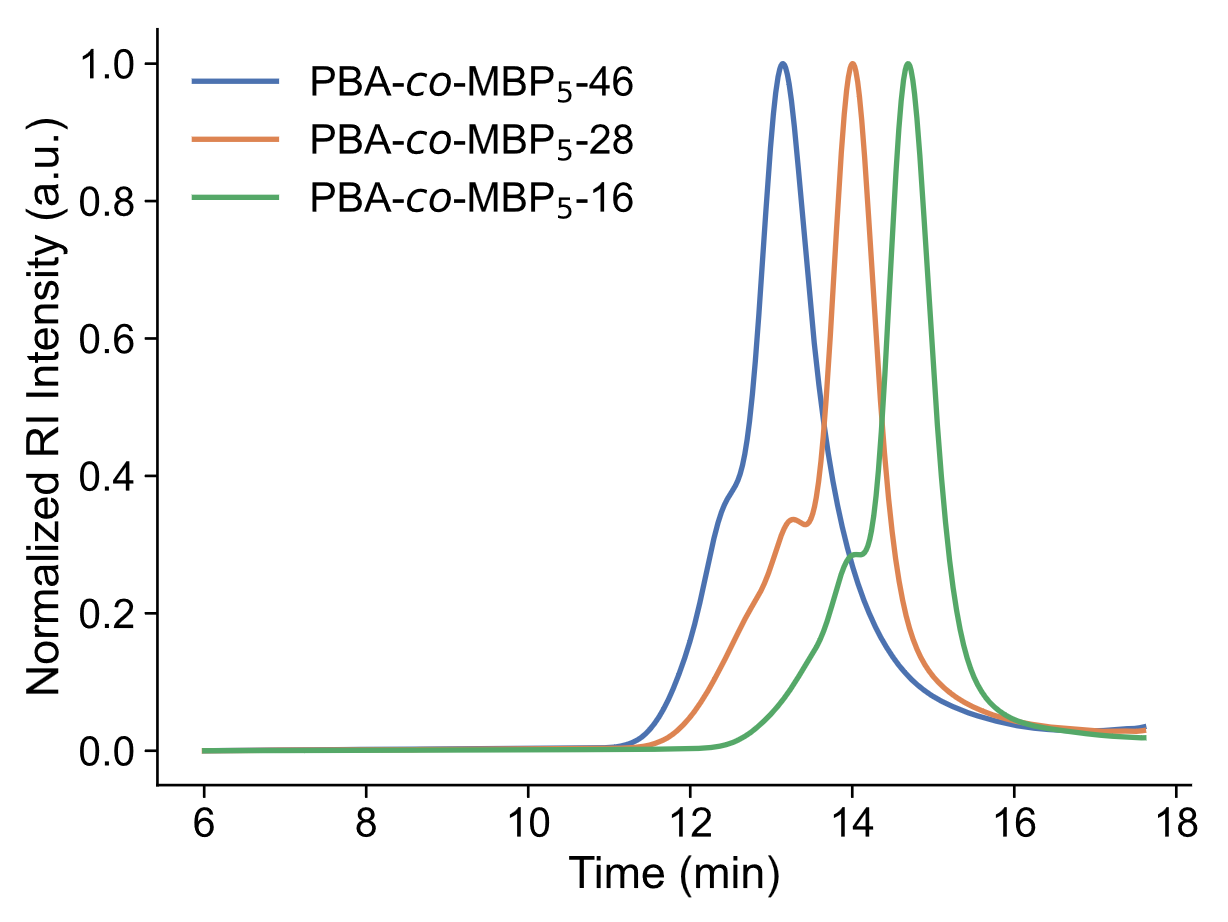

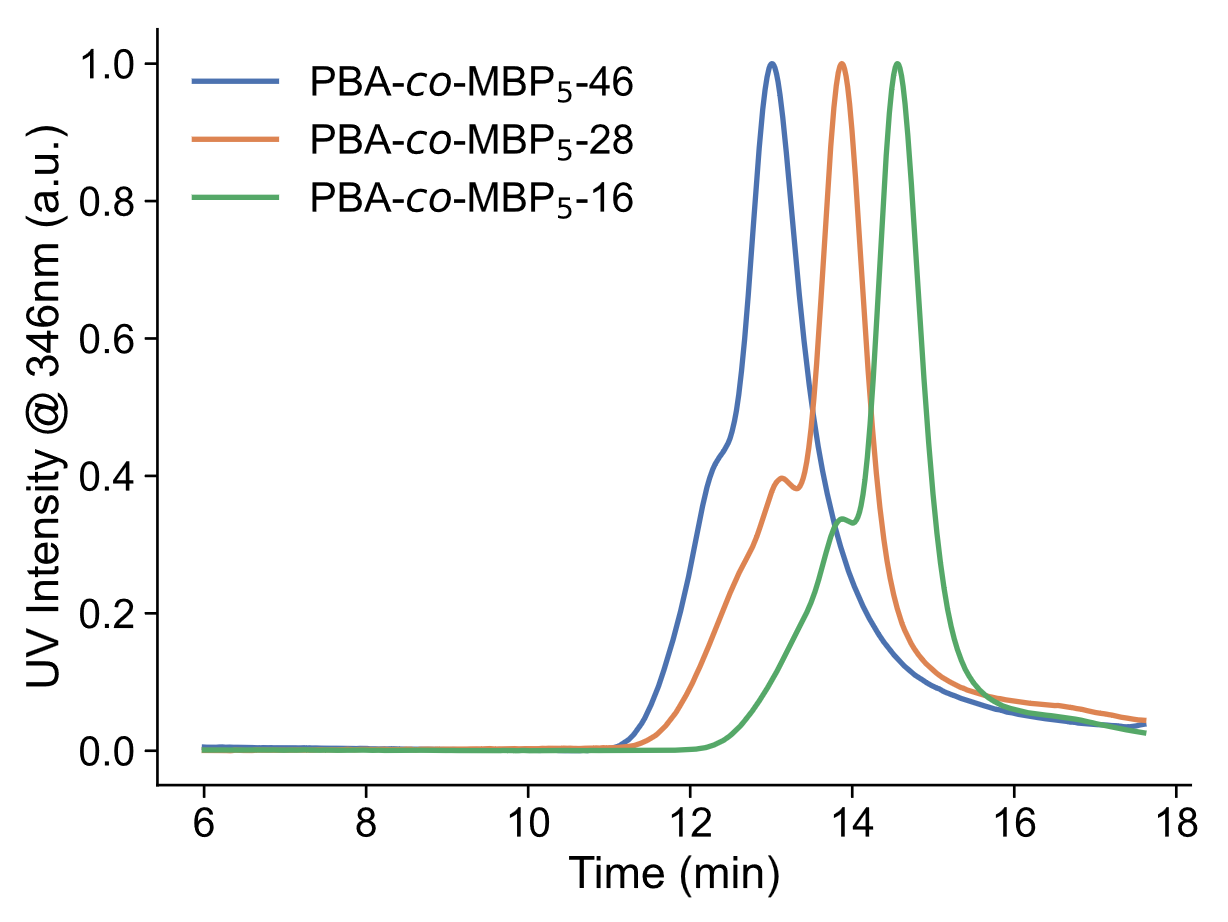


**A**

**B**

**Figure S10**. Normalized size-exclusion chromatography (SEC) elugrams of **PBA-*co*-MBP_5_-YY** copolymers with varying molecular weight and a fixed mol fraction of MBP-acrylate comonomer (5 mol%). (A) Elugrams measured using refractive index (RI) detection and (B) UV detection at λ=365 nm, with both traces scaled by min–max normalization.


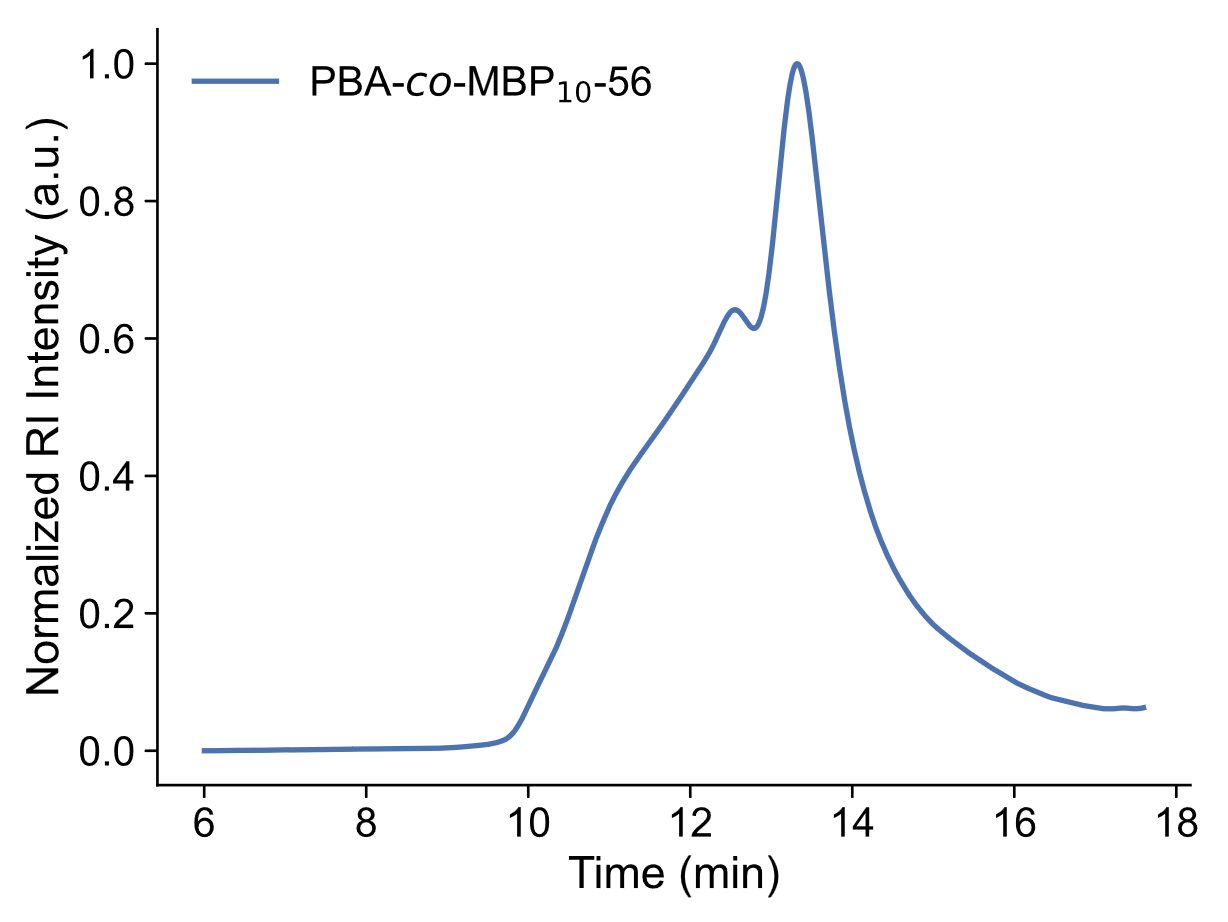

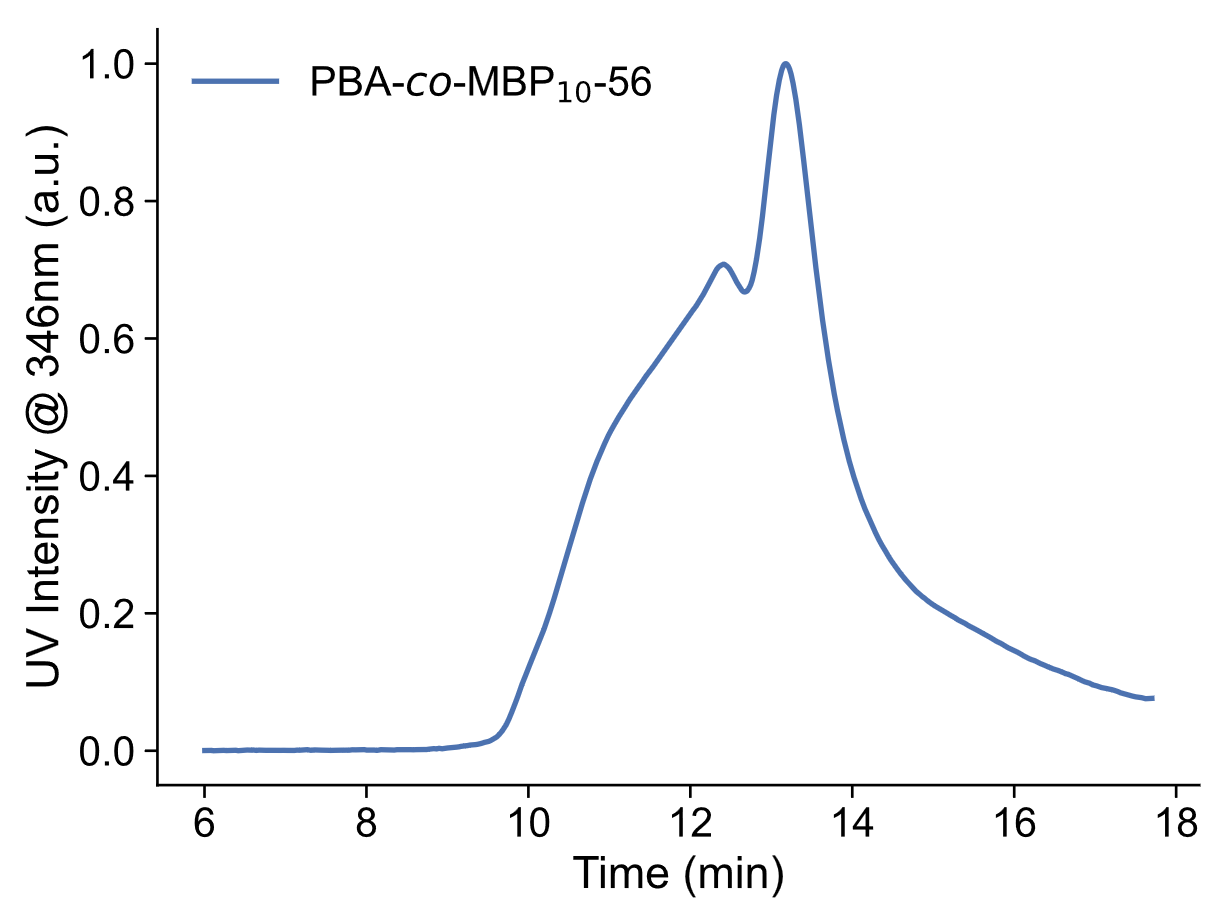


**A**

**B**

**Figure S11**. Normalized size-exclusion chromatography (SEC) elugrams of **PBA-*co*-MBP_10_-56** copolymer with 10 mol% fraction of MBP-acrylate comonomer. (A) Elugrams measured using refractive index (RI) detection and (B) UV detection at λ=365 nm, with both traces scaled by min–max normalization.


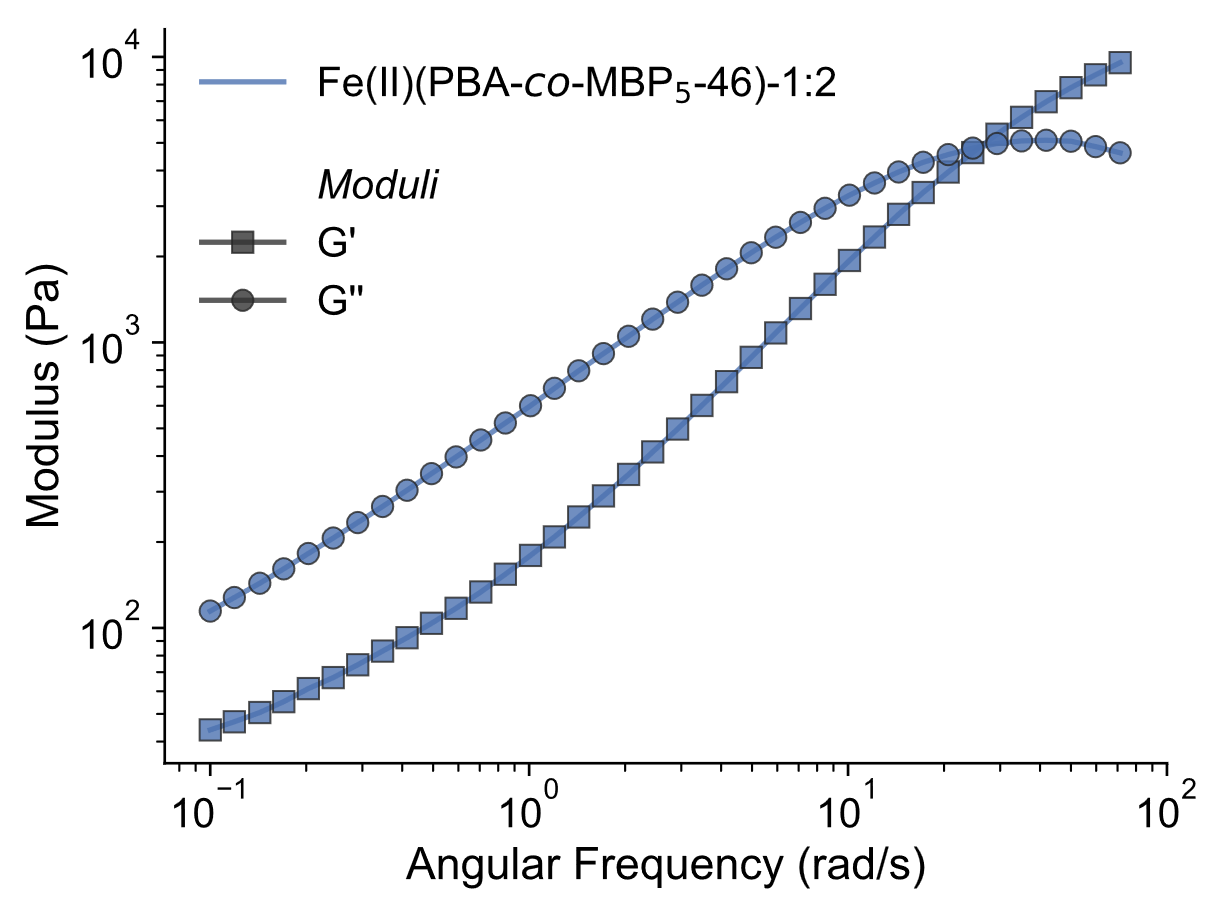


**Figure S12**. Frequency sweep of 25 wt% MSP gel **Fe(II)(PBA-*co*-MBP_5_-46)-1:2**. The sweep was conducted at a strain γ=1%.


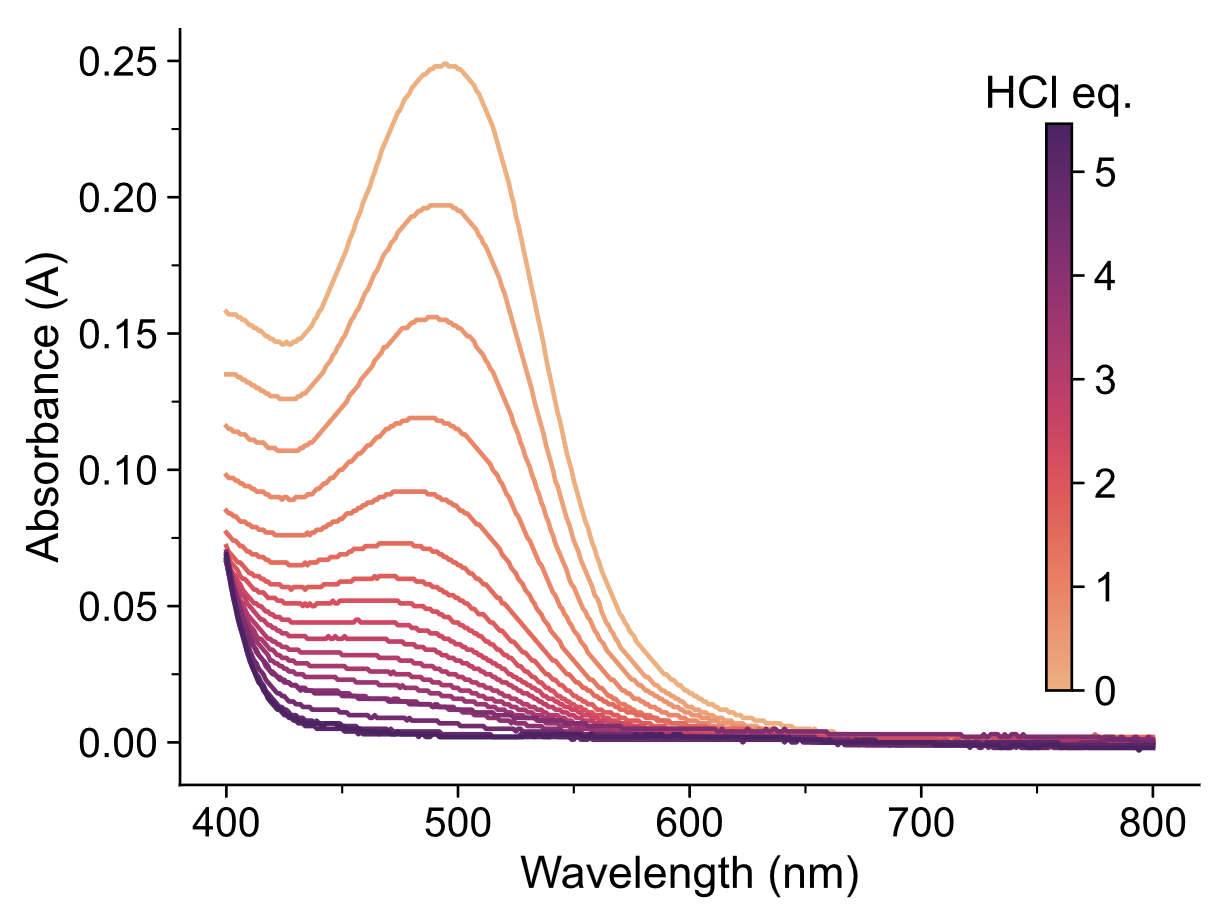

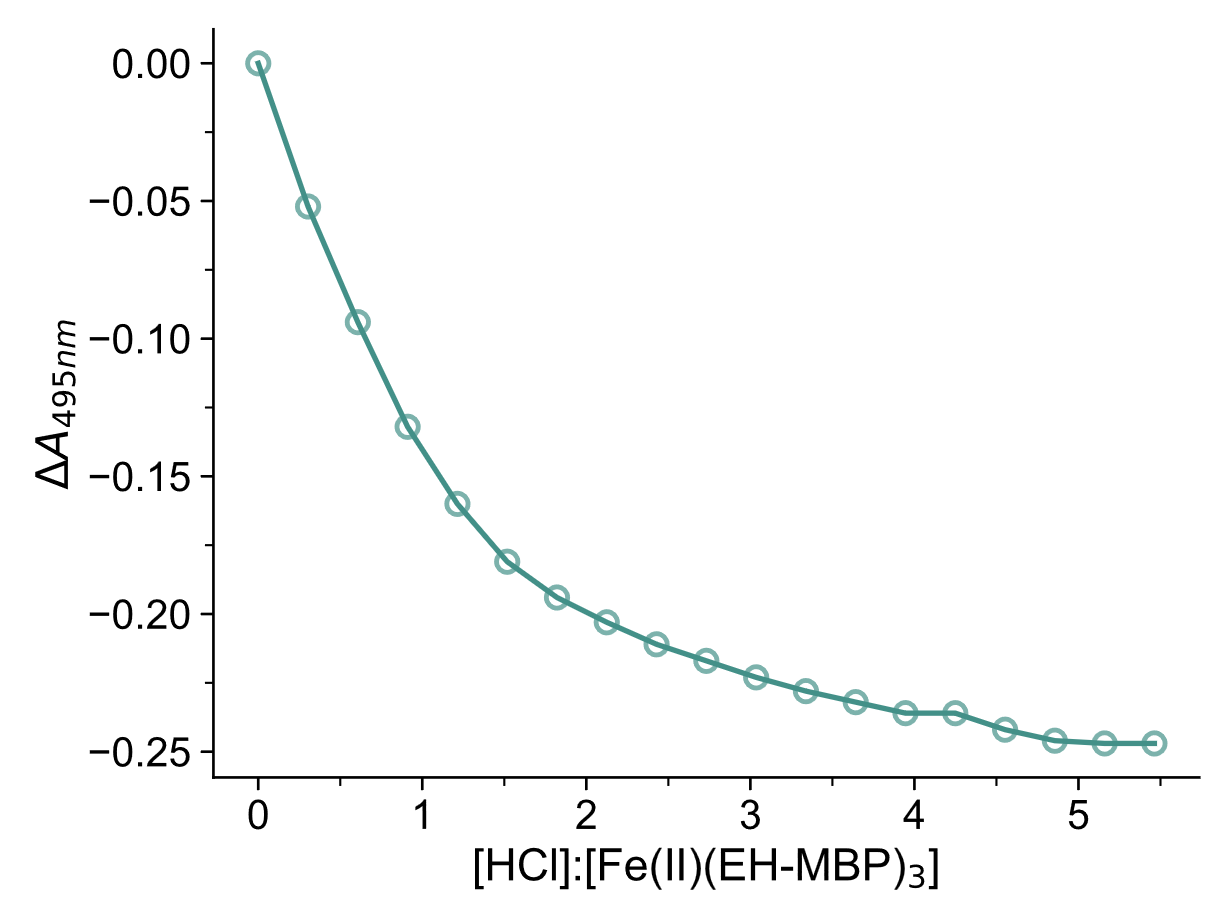


**A**

**B**

**Figure S13**. The UV-Vis spectroscopy monitored titration of **Fe(II)(EH-MBP)_3_** (c=78.1 µM) with aliquots of HCl (c=16 mM) in MeCN. The visible band associated with the MLCT band is monitored. (A) Absorption spectra recorded for the titration (B) line graph showing the change in the absorbance at 495 nm versus the [HCl]:[**Fe(II)(EH-MBP)_3_**] ratio after the addition of each aliquot of HCl.


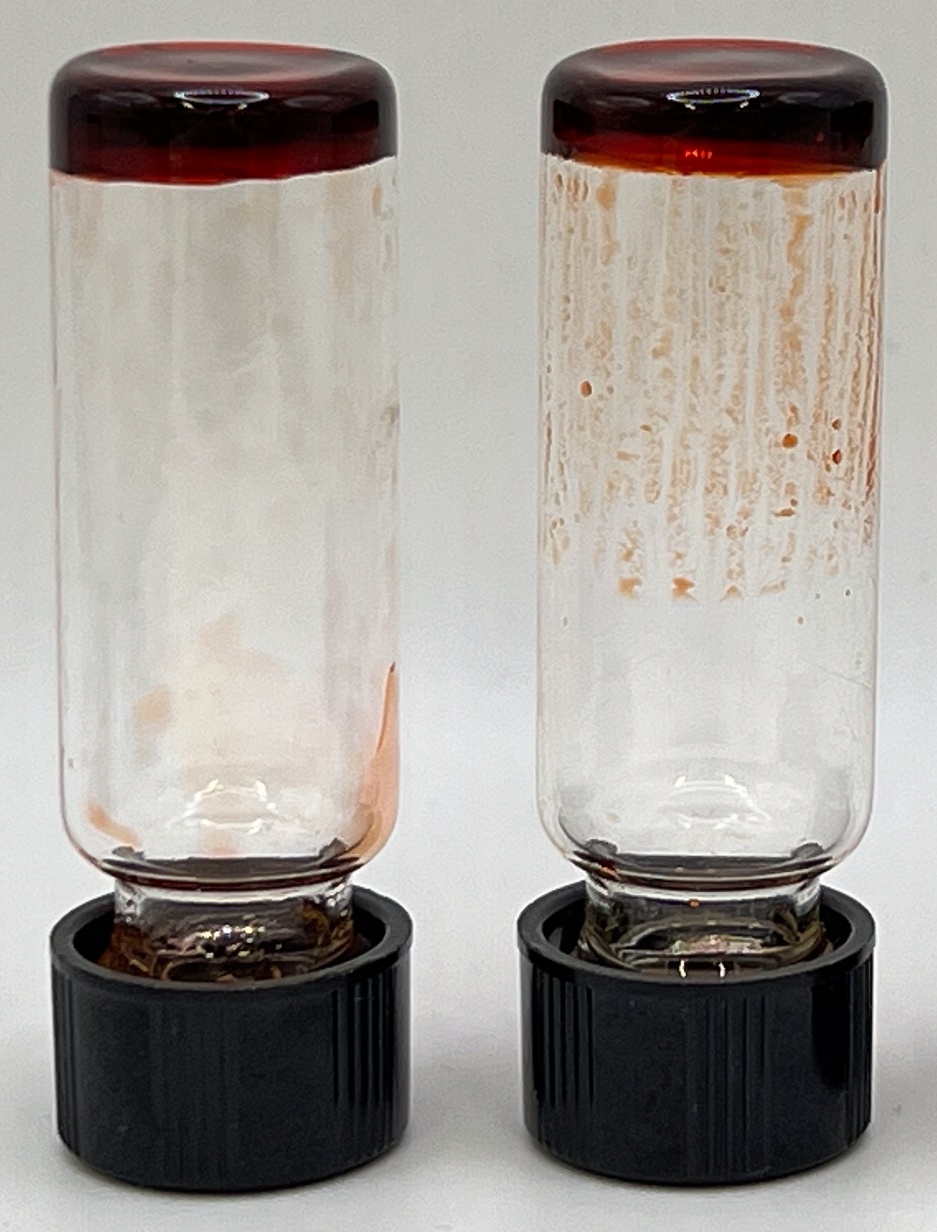


B

A

**Figure S14**. Pictures of **Fe(II)(PBA-*co*-MBP_5_-46)-1:3** MSP gels prepared at 25 wt% (m/m) relative to the mass of chlorobenzene with the addition of MBTT. (A) **Fe(II)(PBA-*co*-MBP_5_-46)-1:3/MBTT_0.25_**, (B) **Fe(II)(PBA-*co*-MBP_5_-46)-1:3/MBTT_1_** where the MBTT_xx_ subscript xx=[MBTT]:[ML], i.e., the equivalence of MBTT to the moles of ML crosslinks.


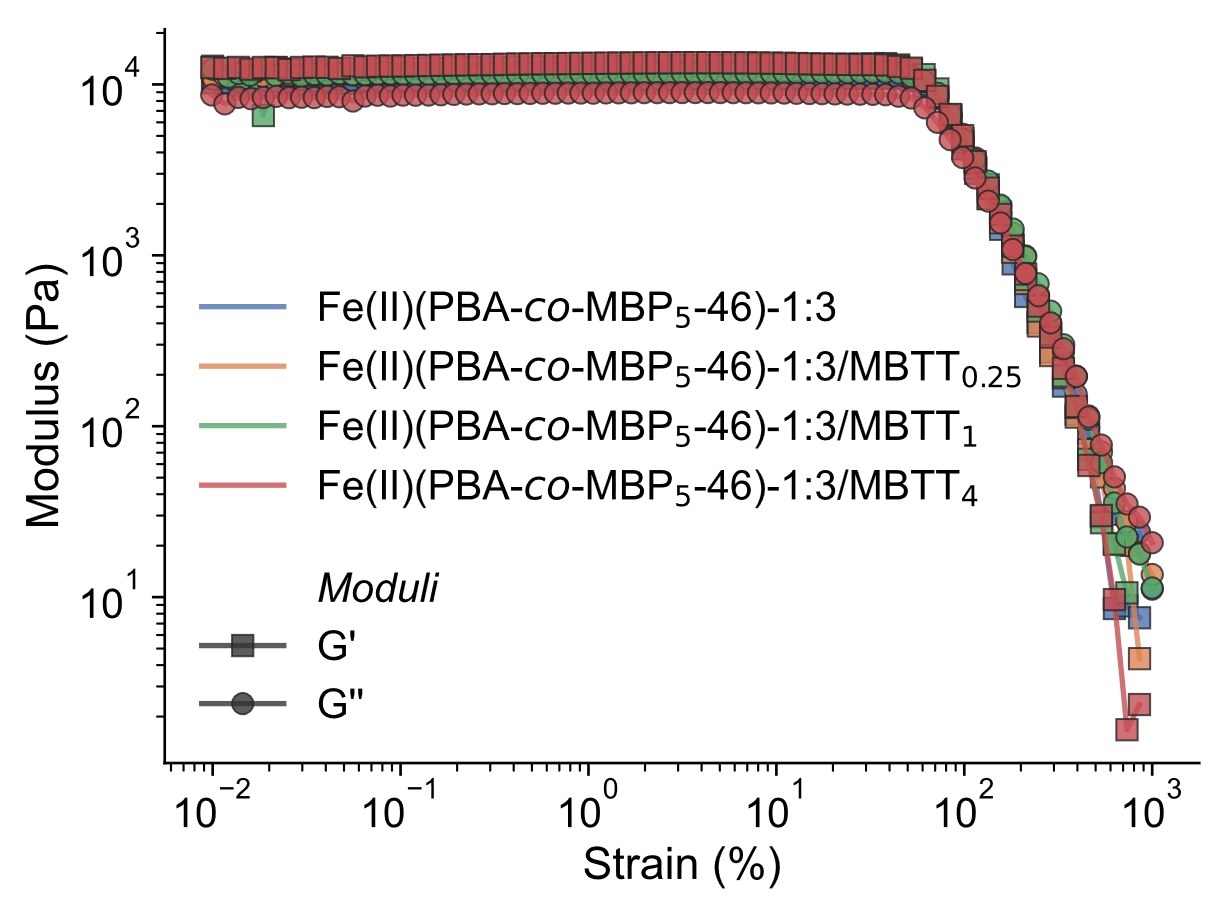

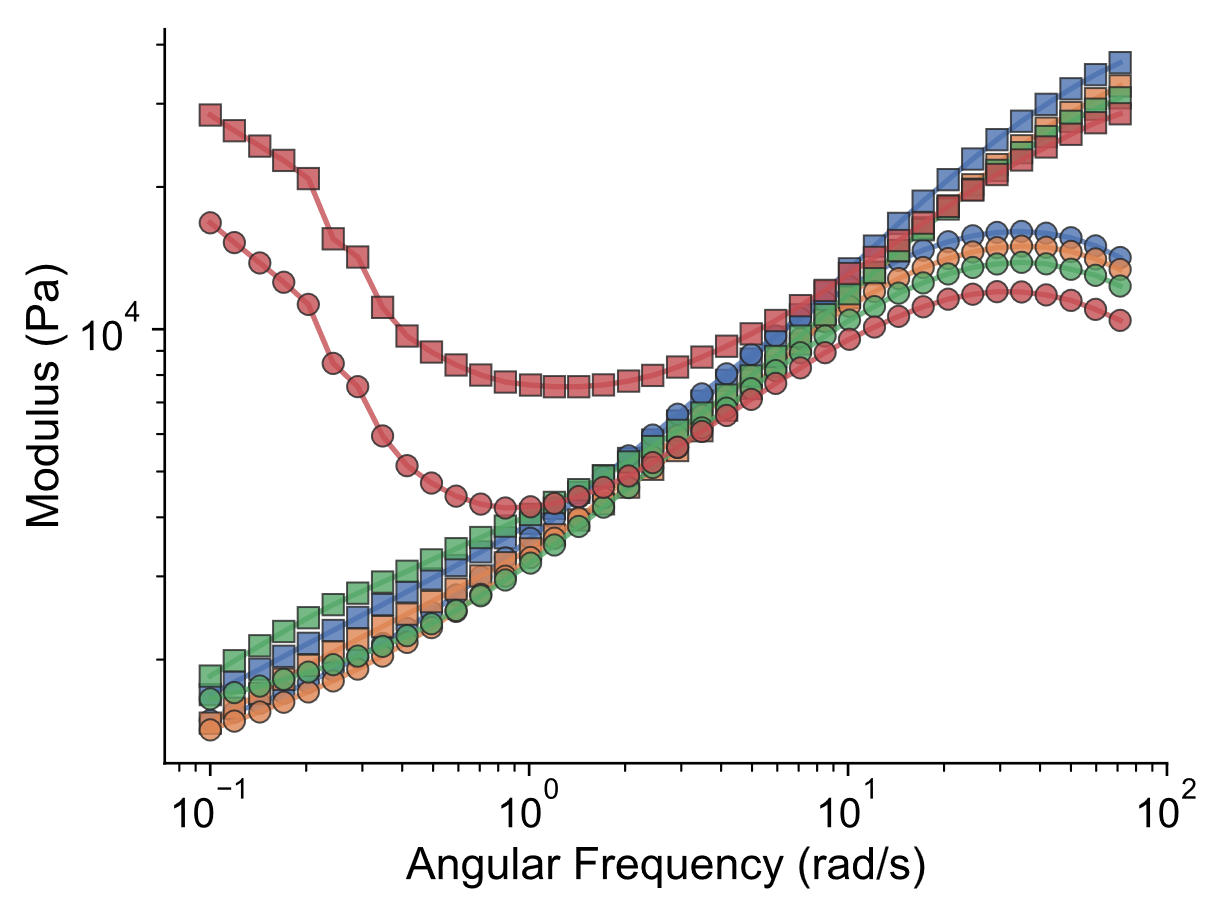


**A**

**B**

**Figure S15**. Small amplitude oscillatory shear (SAOS) rheology data comparing MBTT-loaded with MBTT-free MSP gels **Fe(II)(PBA-***co***-MBP_5_-46)-1:3**, **Fe(II)(PBA-*co*-MBP5-46)-1:3/MBTT_0.25_**, and **Fe(II)(PBA-*co*-MBP5-46)-1:3/MBTT_1_**, and **Fe(II)(PBA-*co*-MBP5-46)-1:3/MBTT_4_**. (A) Amplitude sweeps conducted at an angular frequency ω=10 rad/s. (B) Frequency sweeps conducted at a strain γ=1%. All samples contain 25 wt% of the respective MSP in chlorobenzene.


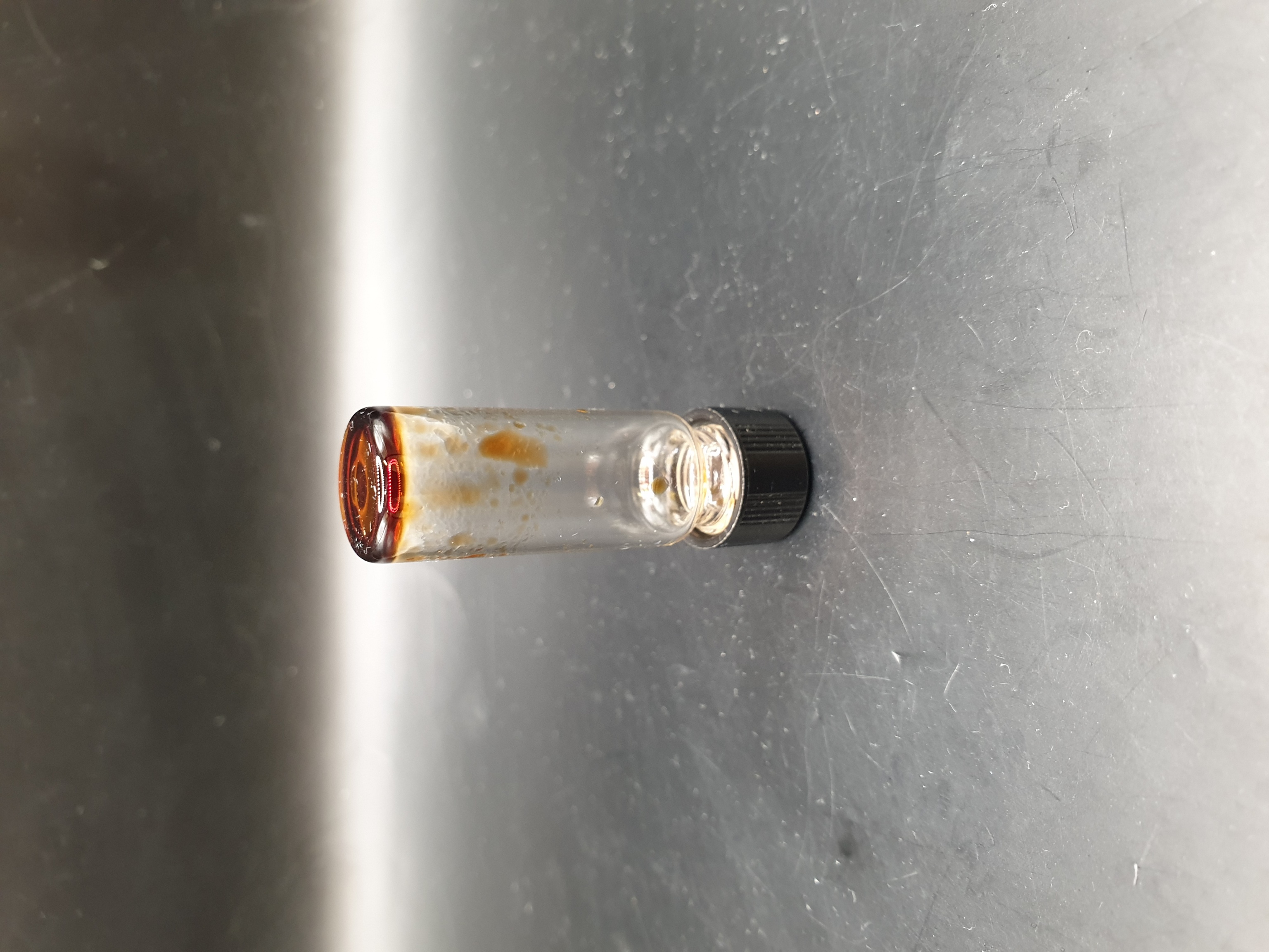

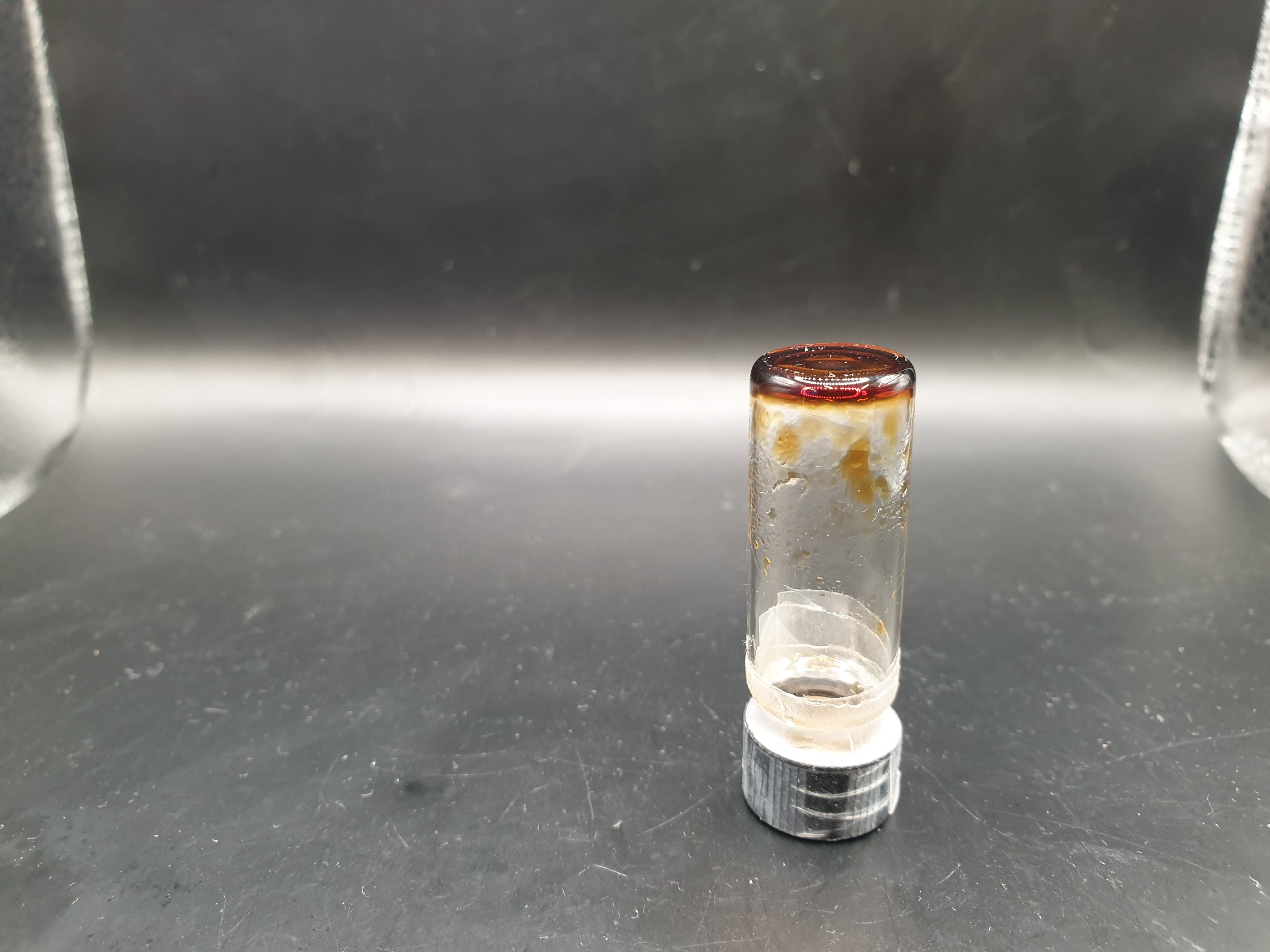

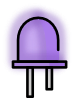


**365 nm**

**Figure S16**. Pictures of a **Fe(II)(PBA-***co***-MBP_5_-46)-1:3/MBTT_0.25_** gel in chlorobenzene before (left) and after (right) irradiation with UV light (365 nm, 90 mW/cm^2^) for 30 min. The MSP content is 25 wt% relative to the mass of chlorobenzene and **Fe(II)(PBA-***co***-MBP_5_-46)-1:3/MBTT_0.25_** contains 0.25 eq. of MBTT relative to the ML complexes.


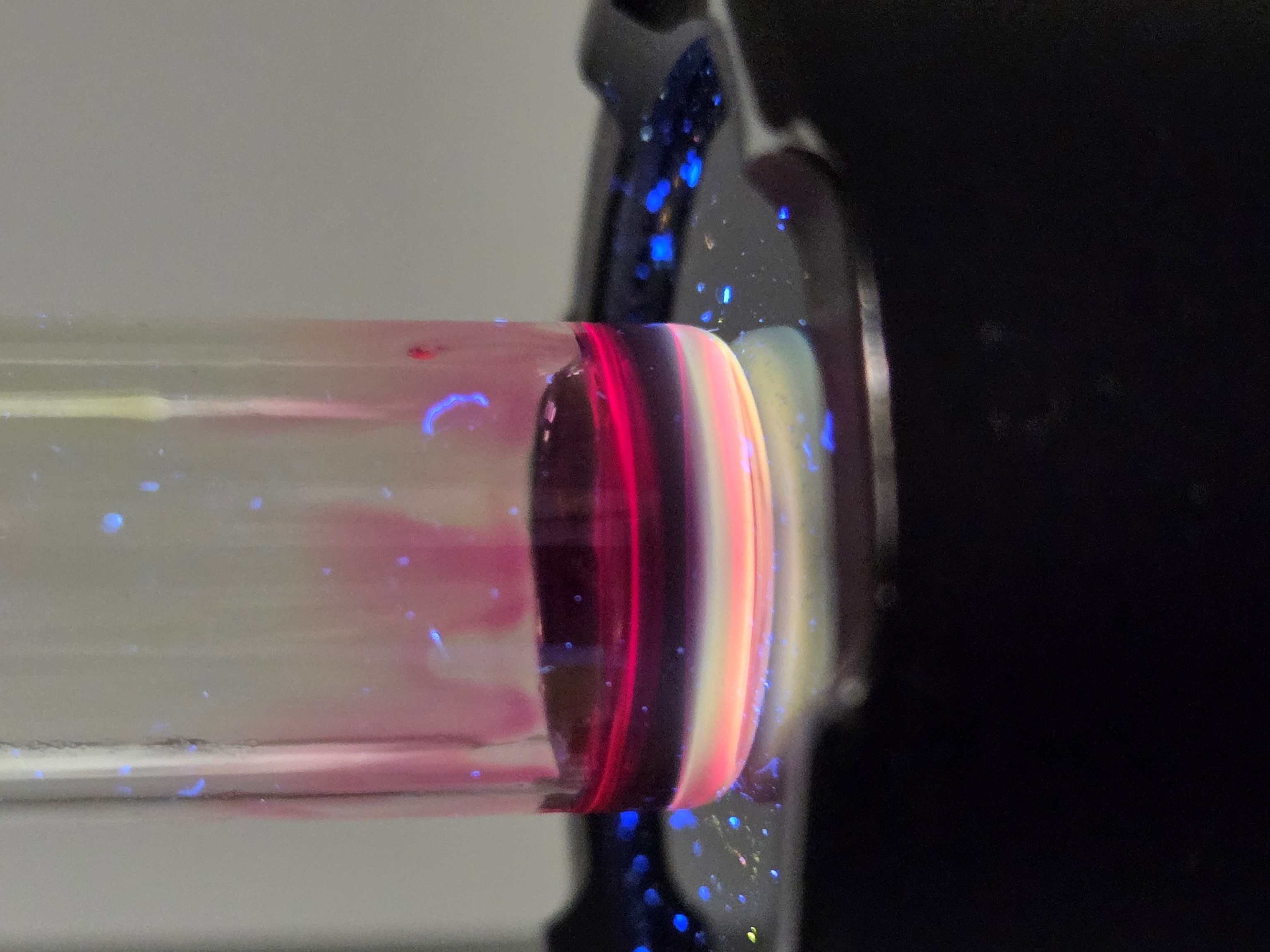


**Figure S17**. Picture of a **Fe(II)(PBA-***co***-MBP_5_-46)-1:3/MBTT_4_** gel in chlorobenzene, loaded with 4 eq. Rhodamine B (0.1 M) dye. The sample was irradiated from the bottom with a UV torch.

**Materials and Instrumentation**

**Materials.** Reagent-grade solvents were obtained from Sigma-Aldrich or Fisher Scientific and utilized without further purification. Metal precursors, including zinc(II) triflate (Zn(OTf)_2_), iron(II) triflate (Fe(OTf)_2_), and copper(II) triflate (Cu(OTf)_2_), were sourced from STREM Chemicals. Iron(III) triflate (Fe(OTf)_3_), cyanomethyl dodecyl trithiocarbonate, acryloyl chloride, and 5-hydroxypyridine-2-carboxylic acid were purchased from Sigma-Aldrich and Fluorochem, respectively. N-Methyl-1,2-phenylenediamine dihydrochloride was acquired from TCI. 2,2’-Azobis(2-methylpropionitrile) (AIBN) was purified via recrystallization from boiling ethanol. To remove acidic residues, 2-(4-methoxystyryl)-4,6-bis(trichloromethyl)-1,3,5-triazine (MBTT) was filtered through a basic alumina plug in acetone, followed by solvent removal *in vacuo*. Residual inhibitors in *n*-butyl acrylate were similarly removed by passage through basic alumina prior to polymerization. Flash column chromatography was performed on a Biotage Isolera One system using Biotage SNAP cartridges, with analytes monitored by UV detection at 254 nm. Two different UV light sources were used in experiments and indicated accordingly; A Thorlabs-purchased UV-mounted LED (M365L3, *λ*=365 nm, *P*=1.29 W, power density on the sample ca. 190 mW/cm^2^) with a collimated lens was controlled with an LEDD1B driver and used for samples held in cuvettes, Tattu UV Torches (*λ*=365 nm, *P*=5 W, power density on the sample ca. 90 mW/cm^2^) were used for the irradiation of vialed samples. A Hönle UV-meter with a flat UVA sensor was used to measure all power densities recorded.

**Ultraviolet Visible (UV-Vis) Absorption Spectroscopy**. Electronic absorption spectra were acquired at ambient temperature using a Shimadzu UV-2401 PC spectrophotometer. Samples were measured in quartz cuvettes with a 1 cm optical path length. Data processing, analysis, and visualization were performed using custom Python scripts utilizing open-source libraries.

**Nuclear magnetic resonance (NMR) spectroscopy**. ^1^H and ^13^C NMR spectra were recorded on a Bruker Avance DPX 400 spectrometer at 400 MHz and 100 MHz, respectively. Chemical shifts were referenced to the residual protic solvent resonance or internal tetramethylsilane (TMS). Data analysis was conducted using MestReNova (v12.0.2). Multiplicities are reported as follows: s (singlet), d (doublet), dd (doublet of doublets), t (triplet), q (quintet), m (multiplet), and br (broad).

**Size Exclusion Chromatography (SEC)**. Molecular weight distributions were determined using an Agilent Technologies 1200 HPLC system equipped with a THF mobile phase (1.0 mL/min, 40 °C), a PL 5 µm mixed-C guard column, and dual SEC columns. Detection was achieved via refractive index (Optilab REX) and laser light scattering (miniDawn TREOS) detectors. Systems were calibrated against poly(methyl methacrylate) (PMMA) standards. Data visualization were performed using custom Python scripts utilizing open-source libraries.

**Rheology**. Dynamic rheological measurements were performed on an Anton Paar MCR 702 MultiDrive rheometer at 25 °C. Samples were loaded onto a roughened 8 mm parallel-plate geometry within a sealed chamber and equilibrated for 1 minute before testing. Reproducibility was confirmed across multiple gel batches.

- **Strain sweeps:** Conducted from 0.1% to 1000% strain at a constant frequency of 10 rad/s (0.5 mm gap).
- **Frequency sweeps:** Performed from 600 to 0.1 rad/s at a fixed strain of 1% (0.5 mm gap).
- **Photo-rheology:** *In-situ* UV irradiation was facilitated by an Omnicure LX500 controller and a 385 nm LED head directed through a quartz bottom plate (0.5 mm gap).

Custom Python-based workflows were employed for all rheological data parsing and plotting.

**Synthetic Methods and Characterization**

**6-(1-Methyl-1H-benzo[d]imidazol-2-yl)pyridin-3-ol (MBP)**


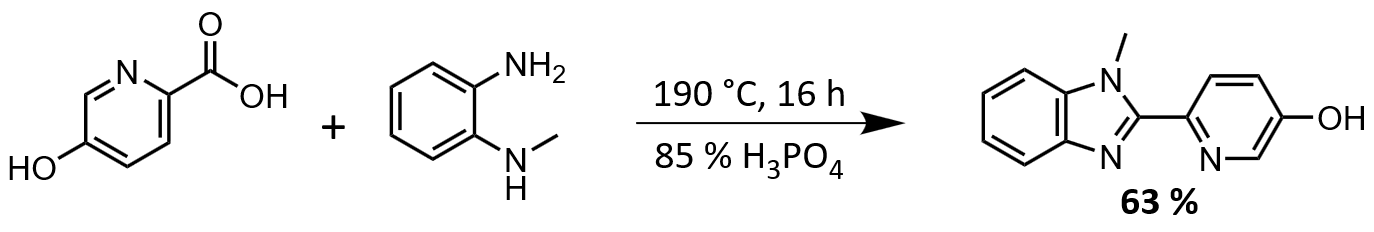


The synthesis of MBP was performed following a previously reported procedure.^1, 2^ In a 100 mL round-bottom flask, 5-hydroxypyridine-2-carboxylic acid (10.00 g, 71.89 mmol) was dissolved in 50 mL of 85% phosphoric acid (H₃PO₄). While stirring, *N*-Methyl-1,2-phenylenediamine dihydrochloride (17.53 g, 89.86 mmol, 1.25 equivalents) was added to the solution. The resulting mixture was degassed by sparging with nitrogen gas for 30 minutes. The reaction mixture was then heated to 190 °C and stirred under a nitrogen atmosphere under reflux conditions overnight. After cooling to room temperature, the mixture was diluted with 40 mL of water and buffered by the addition of 5g of sodium citrate. Concentrated ammonia (NH₃, as ammonium hydroxide) was then added dropwise until the pH reached 6. The mixture was stored in a refrigerator overnight, after which the resulting precipitate was collected by filtration, washed with water, and dried under vacuum at 60 °C to yield **MBP** as a gray solid (10.34 g, 63%).

^1^H NMR (400 MHz, DMSO-d_6_) δ 10.46 (s, 1H, -O*H*), 8.28 (d, J=2.8 Hz, 1H), 8.16 (d, J=8.7 Hz, 1H), 7.68 – 7.64 (m, 1H), 7.61 – 7.56 (m, 1H), 7.36 (dd, J=8.6, 2.9 Hz, 1H), 7.31 – 7.20 (m, 2H), 4.18 (s, 3H).HRMS (ESI): calcd. for [M]^+^ 225.09 and [M + Na]^+^ 248.08, found: 226 and 247.9.^13^C NMR (101 MHz, DMSO-d_6_): δ 154.4, 150.2, 142.1, 141.1, 137.0, 136.8, 125.6, 123.2, 122.6, 122.5, 122.0, 118.9, 110.4, 32.5. HRMS (ESI): calcd. for [M]^+^ 225.09 and [M + Na]^+^ 248.08, found: 226.0 and 247.9.

**2-(5-((2-Ethylhexyl)oxy)pyridin-2-yl)-1-methyl-1H-benzo[d]imidazole** **(EH-MBP)**


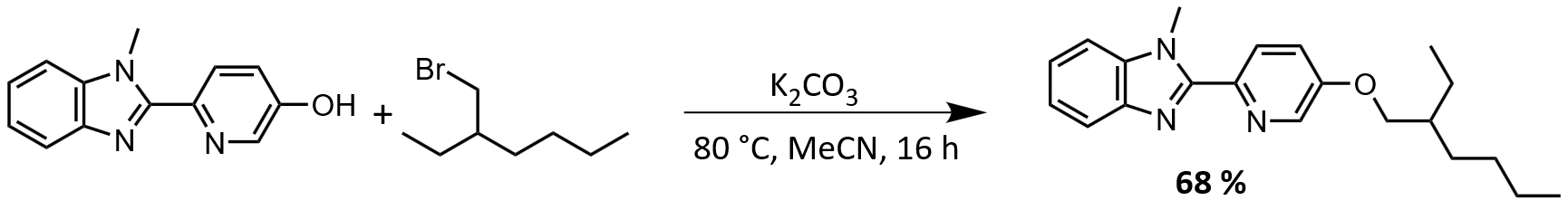


The synthesis of EH-MBP was performed following to a previously reported procedure.^1^ **MBP** (1.00 g, 4.44 mmol) was dissolved in acetonitrile (35 mL) in a round-bottom flask. While stirring, potassium carbonate (excess) was added in portions. Subsequently, 2-ethylhexyl bromide (1.18 mL, 6.66 mmol, 1.5 equiv.) was added dropwise. The reaction mixture was heated to reflux and stirred overnight. After cooling to room temperature, the mixture was filtered to remove inorganic salts, and the solvent was removed under reduced pressure. The crude product was purified by flash column chromatography (DCM/MeOH, gradient from 100:0 to 97:3) to yield **EH-MBP** as an off-white solid, which was dried under vacuum at 60 °C (1.03 g, 68%).

^1^H NMR (400 MHz, CDCl_3_) δ 8.30 (dd, *J*=2.9, 0.7 Hz, 1H), 8.25 (dd, *J*=8.8, 0.6 Hz, 1H), 7.77 – 7.69 (m, 1H), 7.38 – 7.30 (m, 1H), 7.31 – 7.19 (m, 3H), 4.17 (s, 3H), 3.90 (dd, *J*=5.7, 1.0 Hz, 2H), 1.71 (p, *J*=6.1 Hz, 2H), 1.55 – 1.32 (m, 4H), 1.33 – 1.16 (m, 5H), 0.94 – 0.79 (m, 6H).^13^C NMR (101 MHz, CDCl_3_): δ 155.9, 150.7, 143.0, 142.7, 137.4, 136.9, 125.7, 123.0, 122.5, 121.7, 119.8, 109.8, 71.2, 39.5, 32.8, 30.6, 29.2, 23.9, 23.2, 14.2, 11.3. HRMS (ESI): calcd. for [M]^+^ 337 and [M + Na]^+^ 359.9, found: 338 and 360.

**12-((6-(1-methyl-1H-benzo[d]imidazol-2-yl)pyridin-3-yl)oxy)dodecan-1-ol (2)**


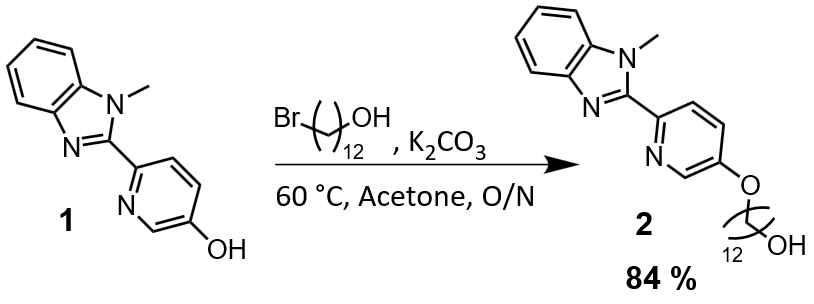


A round-bottom Schlenk flask was charged with compound **1** (5.00 g, 22.21 mmol) and anhydrous acetone (150 mL). To the solution, K_2_CO_3_ was added in excess and stirred at 60 °C. A solution of 12-bromo-1-dodecanol (7.37 g, 27.79 mmol) in a minimal volume of anhydrous acetone was then added dropwise to the stirred suspension. The reaction mixture was heated at reflux and stirred overnight at 60 °C. The solvent was removed under reduced pressure, and the residue was dissolved in dichloromethane (DCM) and filtered. The filtrate was concentrated under reduced pressure, and the product was crystallized from DCM/hexane (70:30 v/v) at −4 °C to yield off-white/pink crystals. The mother liquor was concentrated, and the resulting solid was recrystallized again from DCM/hexane. The combined crystal fractions were dried under vacuum at 60 °C to afford **2** as a pink/off-white solid (9.12 g, 84%).

^1^H NMR (400 MHz, CDCl_3_) δ 8.38 – 8.35 (m, 1H), 8.32 (d, *J*=8.8 Hz, 1H), 7.83 – 7.78 (m, 1H), 7.44 – 7.39 (m, 1H), 7.36 – 7.28 (m, 3H), 4.24 (s, 3H), 4.08 (t, *J*=6.5 Hz, 2H), 3.64 (td, *J*=6.6, 5.3 Hz, 2H), 1.84 (dt, *J*=13.9, 6.6 Hz, 2H), 1.57 – 1.43 (m, 5H), 1.42 – 1.21 (m, 13H). ^13^C NMR (101 MHz, CDCl_3_) δ 155.63, 150.67, 142.95, 142.67, 137.34, 136.86, 125.76, 123.00, 122.55, 121.67, 119.81, 109.84, 68.71, 63.14, 32.96, 32.73, 29.72, 29.66, 29.64, 29.62, 29.57, 29.40, 29.21, 26.03, 25.90. HRMS (ESI): calcd. for [M + Na]^+^ 432.56 and found 432.2.

**12-((6-(1-methyl-1H-benzo[d]imidazol-2-yl)pyridin-3-yl)oxy)dodecyl acrylate (3, MBP-acryalte)**


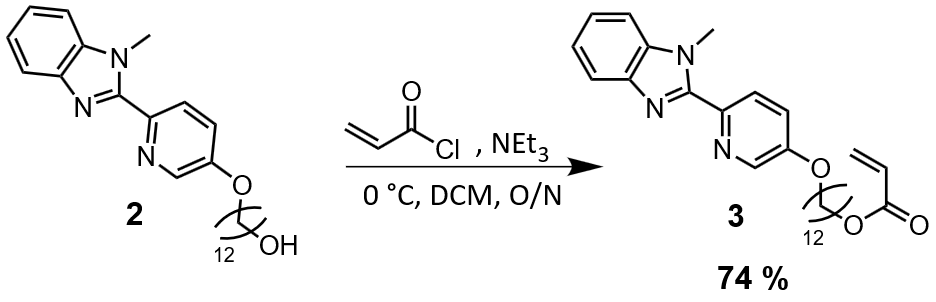


A round-bottom flask was charged with compound **2** (5.27 g, 12.90 mmol) and anhydrous DCM under a nitrogen atmosphere. Triethylamine (5.40 mL, 38.74 mmol) was added, and the mixture was stirred at room temperature for 15 min. The flask was cooled in an ice bath, and acryloyl chloride (1.90 mL, 23.39 mmol) was added dropwise. The reaction was stirred for 16 h, allowing the temperature to return to ambient under an argon atmosphere. The solvent was removed under reduced pressure, and the resulting solid was suspended in THF and filtered. The filtrate was dissolved in DCM, washed twice with aqueous NaHCO_3_ and once with brine, then dried over anhydrous Na_2_SO_4_. After solvent removal in vacuo, the crude product was purified by flash column chromatography (silica gel; DCM/MeOH 99:1 → 97:3 v/v) to yield **3** as an off-white solid (4.71 g, 80%).

^1^H NMR (400 MHz, CDCl_3_) δ 8.37 (dd, *J*=3.0, 0.6 Hz, 1H), 8.32 (dd, *J*=8.8, 0.6 Hz, 1H), 7.83 – 7.78 (m, 1H), 7.45 – 7.39 (m, 1H), 7.36 – 7.28 (m, 3H), 6.39 (dd, *J*=17.3, 1.5 Hz, 1H), 6.12 (dd, *J*=17.4, 10.4 Hz, 1H), 5.81 (dd, *J*=10.4, 1.5 Hz, 1H), 4.24 (s, 3H), 4.15 (t, *J*=6.7 Hz, 2H), 4.08 (t, *J*=6.5 Hz, 2H), 1.84 (p, *J*=6.7 Hz, 2H), 1.72 – 1.62 (m, 3H), 1.48 (q, *J*=7.3 Hz, 2H), 1.32 (d, *J*=14.5 Hz, 13H). ^13^C NMR (101 MHz, CDCl_3_) δ 166.47, 155.62, 150.67, 142.98, 142.69, 137.36, 136.84, 130.54, 128.79, 125.74, 122.98, 122.52, 121.64, 119.81, 109.83, 68.72, 64.84, 32.73, 29.67, 29.63, 29.48, 29.37, 29.25, 28.75, 26.08, 26.05. HRMS (ESI): calcd. for [M + Na]^+^ 486.27, found: 486.1.

**General procedure for the preparation of poly(*n*-butyl acrylate-*co*-MBP) PBA-*co*-MBP_xx_-YY.
The following is an example given for PBA-*co*-MBP_5_-46.**


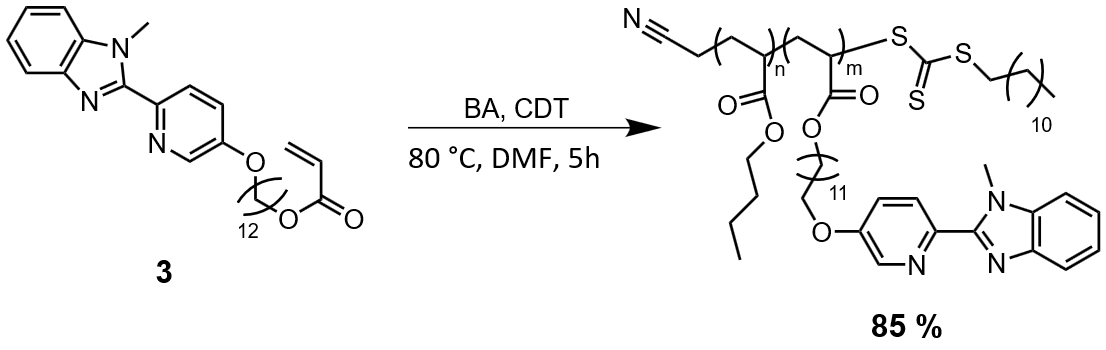


A 50 mL flask was charged with **MBP-acrylate** (0.99 g, 2.14 mmol), cyanomethyl dodecyl trithiocarbonate (CDT, 39.8 mg, 0.125 mmol), AIBN (10.80 mg, 0.031 mmol), *n*-butyl acrylate (5.24 g, 5.83 mL, 40.9 mmol), dioxane (500 μL, internal reference), and DMF (6 mL). The mixture was stirred at room temperature until complete dissolution of solids was achieved, then purged with nitrogen for 30 min under vigorous stirring. Polymerization was initiated by heating to 80 °C and allowed to proceed for 5 h until 80 – 90 % conversion was achieved. The reaction was quenched by exposure to air, cooled to room temperature, and precipitated into cold methanol with stirring. The resulting polymer was collected, dried under reduced pressure at 60 °C overnight, and obtained as a soft, light orange solid with an 85% yield. Polymers of varying molecular weight were synthesized by altering the monomer-to-CDT ratio while maintaining the CDT-to-AIBN ratio at 0.25.

^1^H NMR (400 MHz, CDCl_3_) δ 8.36 (s, 1H), 8.32 (d, *J*=8.8 Hz, 1H), 7.83 – 7.78 (m, 1H), 7.44 – 7.38 (m, 1H), 7.35 – 7.28 (m, 3H), 4.24 (s, 3H), 4.15 – 3.89 (m, 45H), 2.51 – 2.13 (m, 22H), 2.02 – 1.78 (m, 12H), 1.76 – 0.72 (m, 214H).

**PBA-*co*-MBP_5_-16: MBP-acrylate** (0.35 g, 0.76 mmol), cyanomethyl dodecyl trithiocarbonate (CDT, 55.7 mg, 0.176 mmol), AIBN (7.22 mg, 0.044 mmol), *n*-butyl acrylate (1.85 g, 2.07 mL, 14.44 mmol), dioxane (200 μL, internal reference), and DMF (1.8 mL).

^1^H NMR (400 MHz, CDCl_3_) δ 8.36 (s, 1H), 8.32 (d, *J*=8.8 Hz, 1H), 7.83 – 7.78 (m, 1H), 7.44 – 7.38 (m, 1H), 7.35 – 7.28 (m, 3H), 4.24 (s, 3H), 4.15 – 3.89 (m, 43H), 2.51 – 2.13 (m, 19H), 2.02 – 1.78 (m, 13H), 1.76 – 0.72 (m, 234H).

**PBA-*co*-MBP_5_-28: MBP-acrylate** (0.35 g, 0.76 mmol), cyanomethyl dodecyl trithiocarbonate (CDT, 27.9 mg, 0.088 mmol), AIBN (3.60 mg, 0.022 mmol), *n*-butyl acrylate (1.85 g, 2.07 mL, 14.44 mmol), dioxane (200 μL, internal reference), and DMF (1.8 mL).

^1^H NMR (400 MHz, CDCl_3_) δ 8.36 (s, 1H), 8.32 (d, *J*=8.8 Hz, 1H), 7.83 – 7.78 (m, 1H), 7.44 – 7.38 (m, 1H), 7.35 – 7.28 (m, 3H), 4.24 (s, 3H), 4.15 – 3.89 (m, 42H), 2.51 – 2.13 (m, 19H), 2.02 – 1.78 (m, 12H), 1.76 – 0.72 (m, 228H).

**PBA-*co*-MBP_10_-56: MBP-acrylate** (0.63 g, 1.36 mmol), cyanomethyl dodecyl trithiocarbonate (CDT, 14.0 mg, 0.044 mmol), AIBN (1.80 mg, 0.011 mmol), *n*-butyl acrylate (1.57 g, 1.76 mL, 12.3 mmol), dioxane (200 μL, internal reference), and DMF (2.3 mL).

^1^H NMR (400 MHz, CDCl_3_) δ 8.36 (s, 1H), 8.32 (d, *J*=8.8 Hz, 1H), 7.83 – 7.78 (m, 1H), 7.44 – 7.38 (m, 1H), 7.35 – 7.28 (m, 3H), 4.24 (s, 3H), 4.15 – 3.89 (m, 21H), 2.51 – 2.13 (m, 10H), 2.02 – 1.78 (m, 7H), 1.76 – 0.72 (m, 116H).

**MSP gel preparation**

**PBA-*co*-MBP_xx_-YY** (0.1 g) was dissolved in CHCl_3_ (3 mL, pre-purified by passage through basic Al₂O₃) in a glass vial under magnetic stirring or vortexing to ensure complete dissolution. A stoichiometric amount of Fe(OTf)₂ solution (0.057 M in MeCN; e.g., [Fe(OTf)₂]:[MBP]=0.33) was added while vortex mixing. The solvent was evaporated in a well-ventilated fume hood, and the resulting solids were re-swelled in chlorobenzene to the desired concentration (typically 25 wt% m/m).

**Preparation MSP gels containing MBTT**

The same general gel preparation procedure was followed, except that the desired amount of MBTT was incorporated into the **PBA-*co*-MBP_xx_-YY** /metal salt mixture prior to re-swelling in chlorobenzene.

**Photo-triggered MSP gel disassembly**

A vial containing the gel was placed on a black sheet of paper, to prevent reflections, and irradiated by three UVA (365 nm) torches from different angles to ensure complete irradiation.

**General procedure for titrations monitored by UV-Vis absorption**

A stock solution of **EH-MBP** (24 μM) in MeCN was prepared and used for all titrations unless otherwise noted. A stock solution of the metal salt in MeCN, typically between 35 – 70 mM was prepared, from which aliquots (typically 2–10 μL) were added to the ligand solution. After each addition, the mixture was continuously stirred and allowed to equilibrate for at least 1 min before the next addition; occasional longer intervals were used to confirm the absence of time-dependent effects. UV–Vis absorption spectra were recorded after each addition.

For acid decomplexation experiments, the same procedure was followed, except that *in situ*–formed complexes were titrated with an HCl solution in MeCN (2–10 μL).

**General procedure for titrations monitored by ^1^H-NMR spectroscopy**

A solution of **EH-MBP** (±44.0 mM) in CD_3_CN:CDCl_3_ (8:2) was prepared, of which 0.6 mL was placed in an NMR tube. A concentrated solution of the metal salt in MeOH, typically between 0.24 – 0.28 M was prepared and added in aliquots (typically 2–10 μL) to the NMR tube containing the **EH-MBP** solution. After each addition, the mixture was continuously stirred using a vortex mixer and allowed to equilibrate for at least 1 min before measuring the ^1^H-NMR spectrum.

**NMR Characterization**


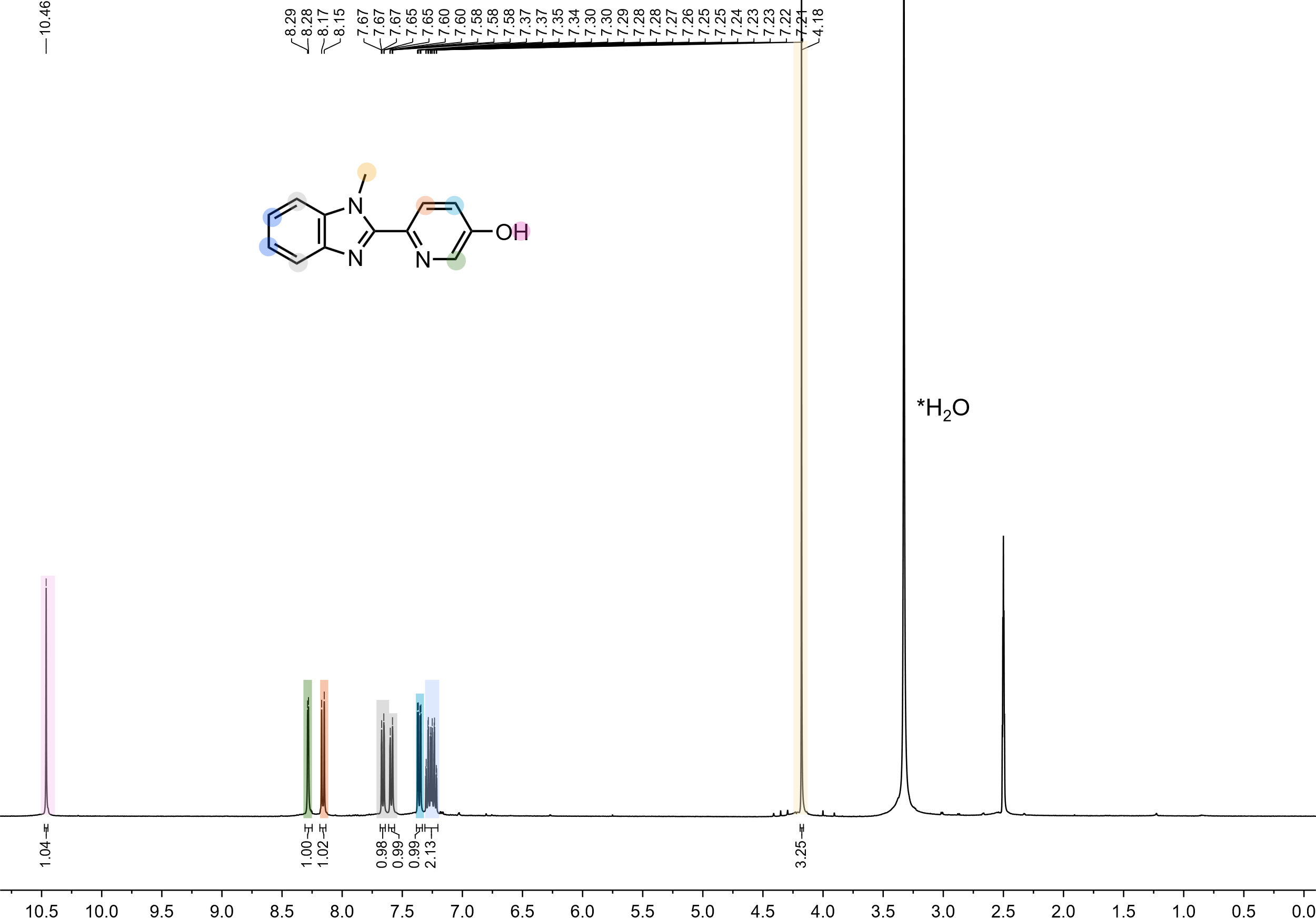


**
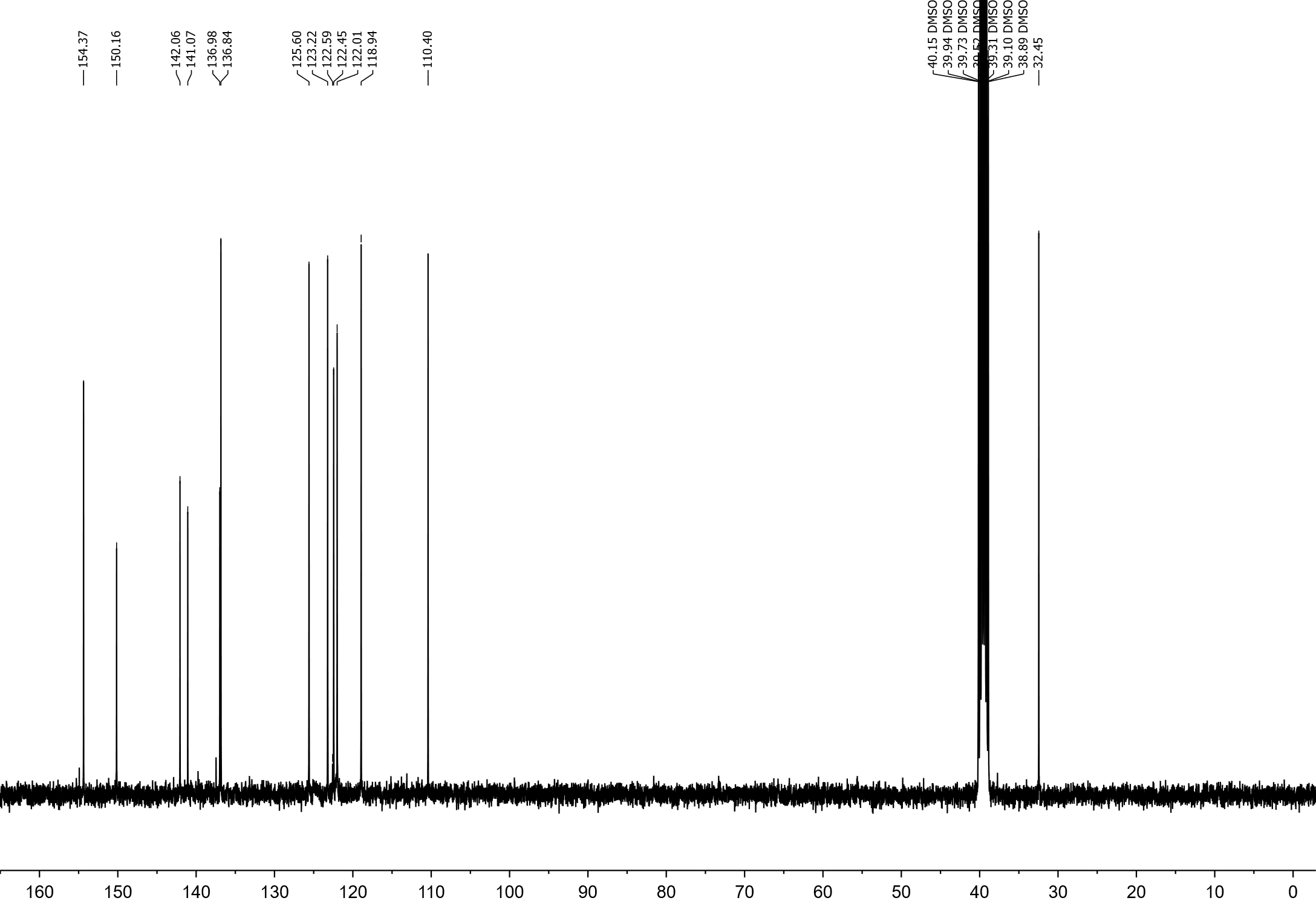
**

**Figure S18**. ^1^H-NMR (DMSO-d_6_, 400 MHz, top) and ^13^C-NMR (DMSO-d_6_, 100 MHz, bottom) spectra of **MBP**.

**
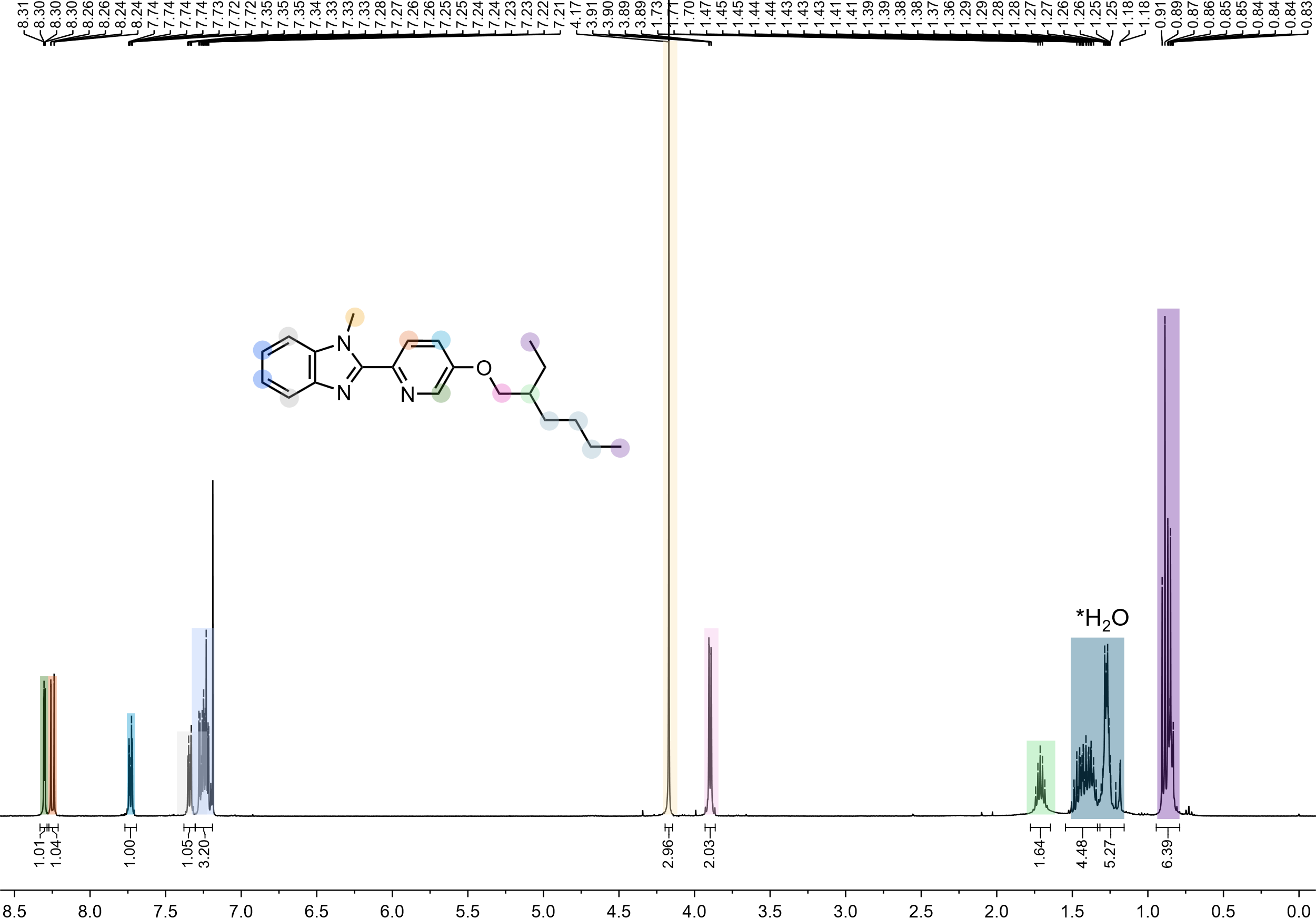
**


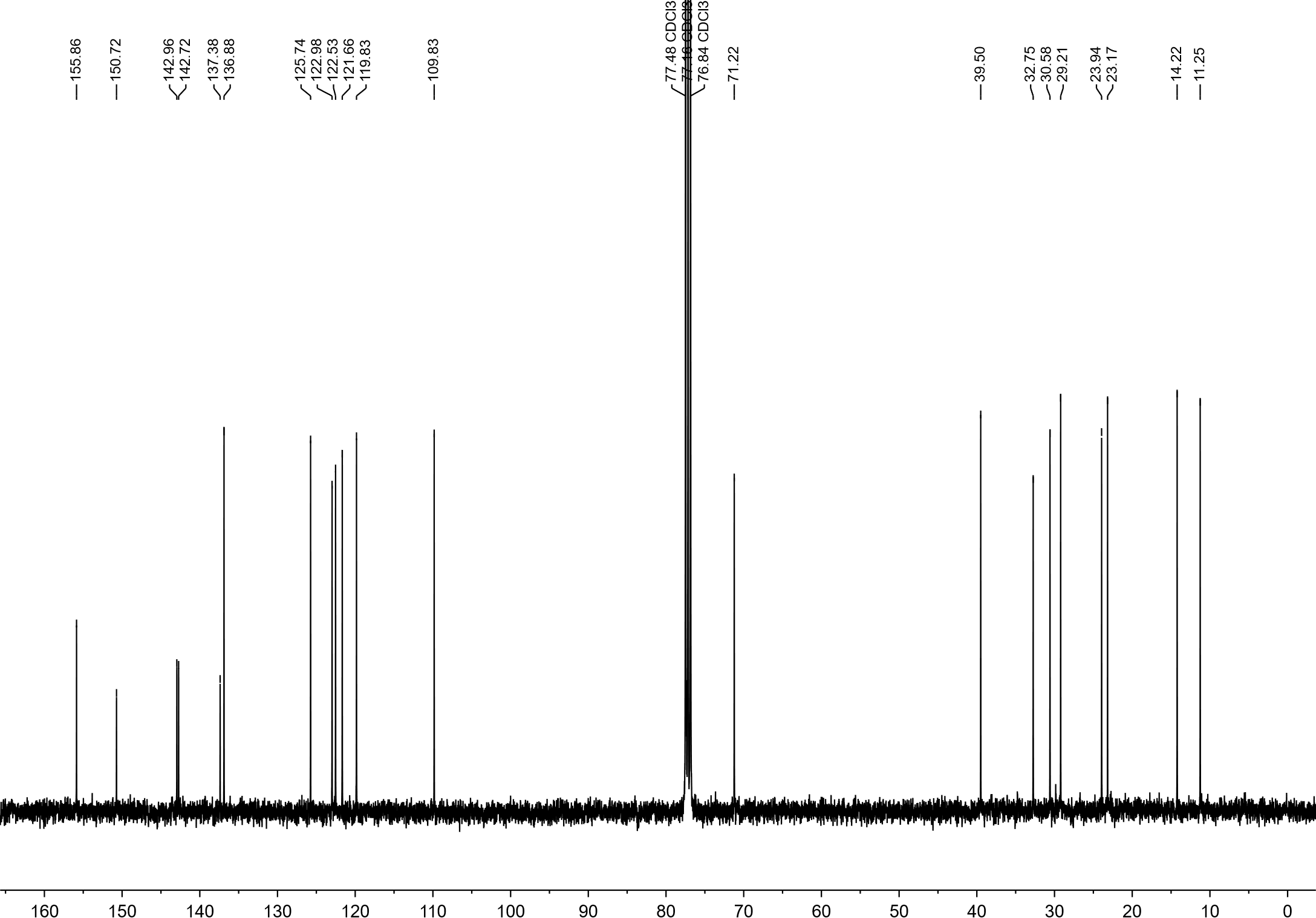


**Figure S19**. ^1^H-NMR (CDCl_3_, 400 MHz, top) and ^13^C-NMR (CDCl_3_, 100 MHz, bottom) spectra of **EH-MBP**.


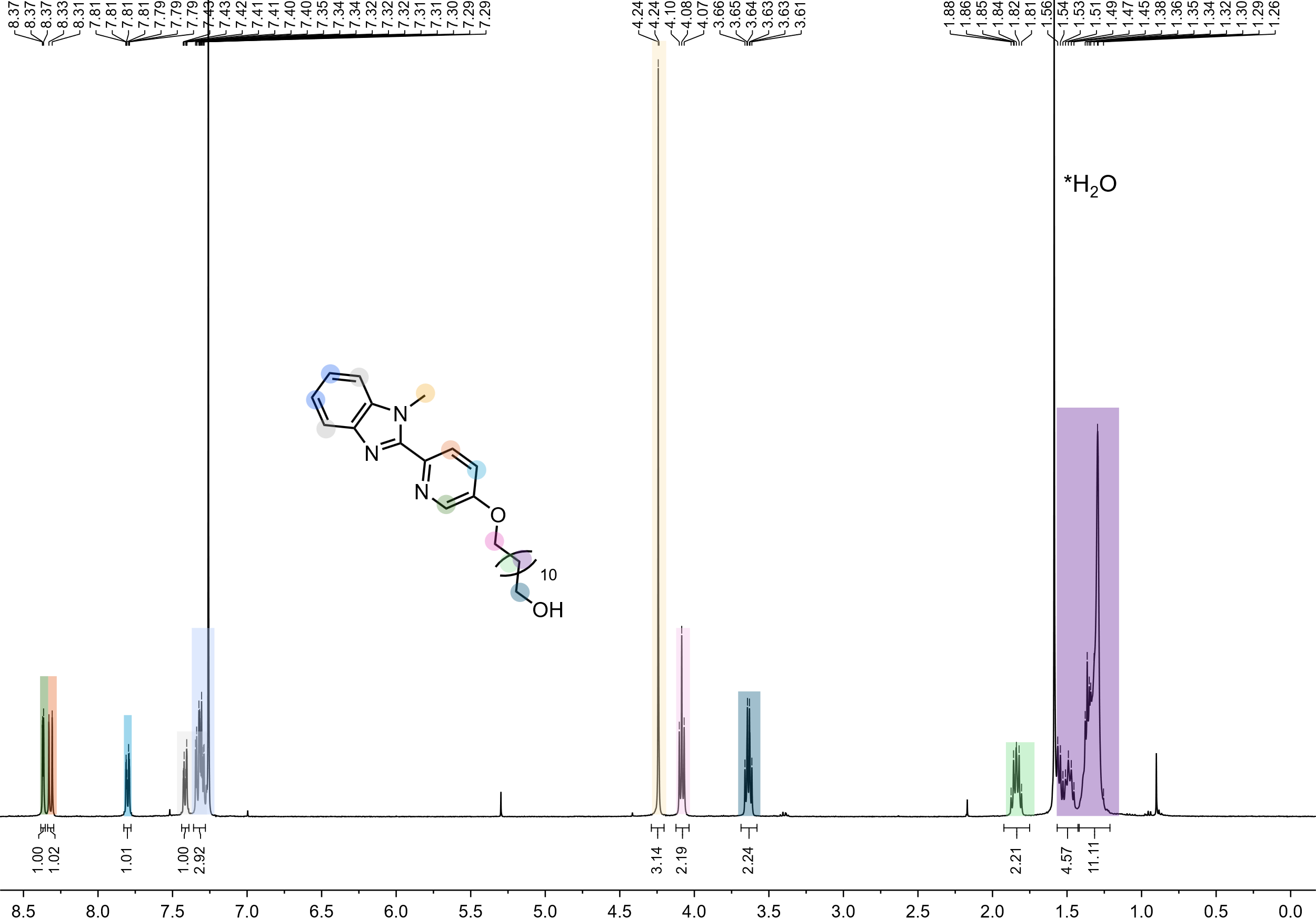


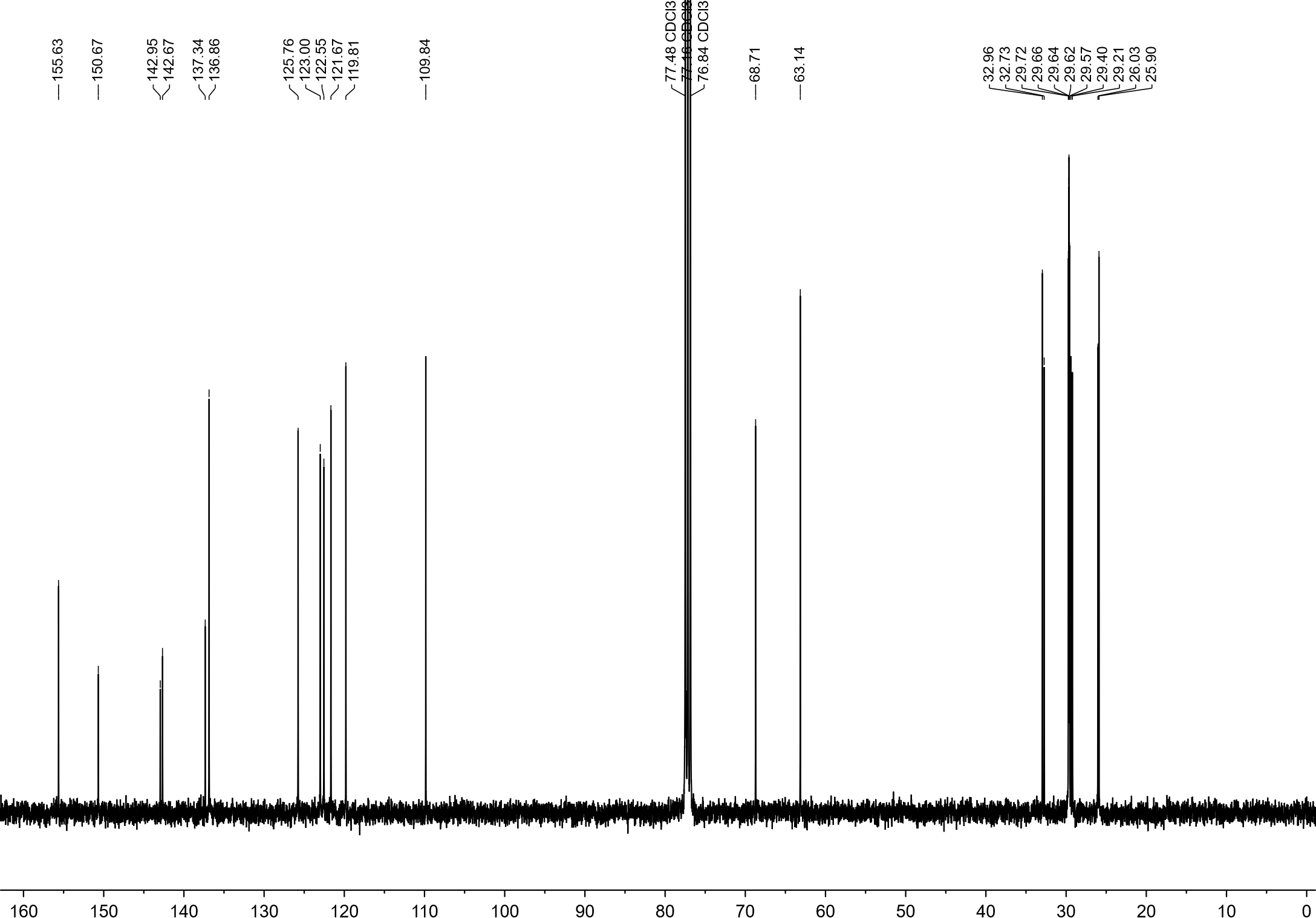


**Figure S20**. ^1^H-NMR (CDCl_3_, 400 MHz, top) and ^13^C-NMR (CDCl_3_, 100 MHz, bottom) spectra of **2**.


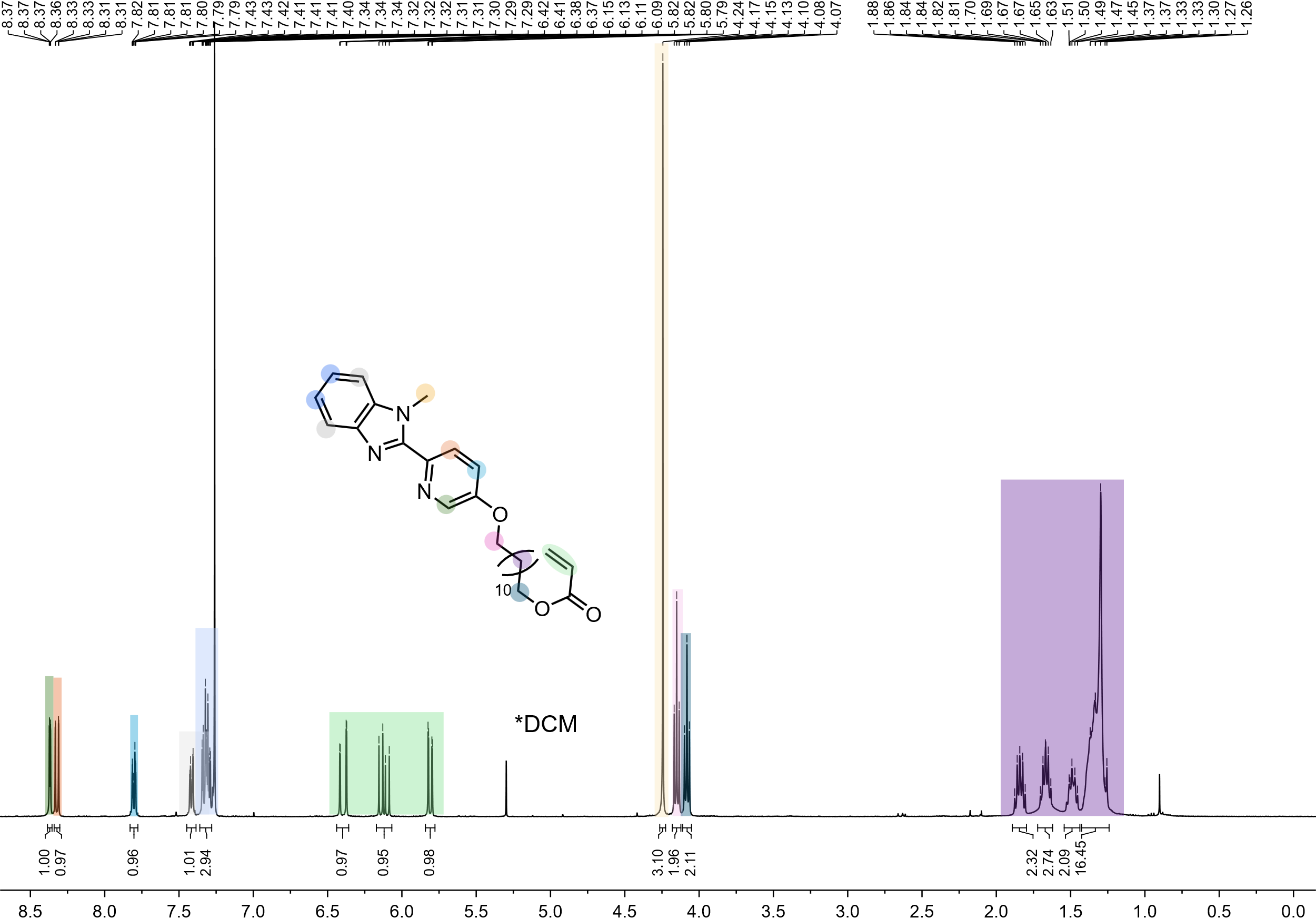


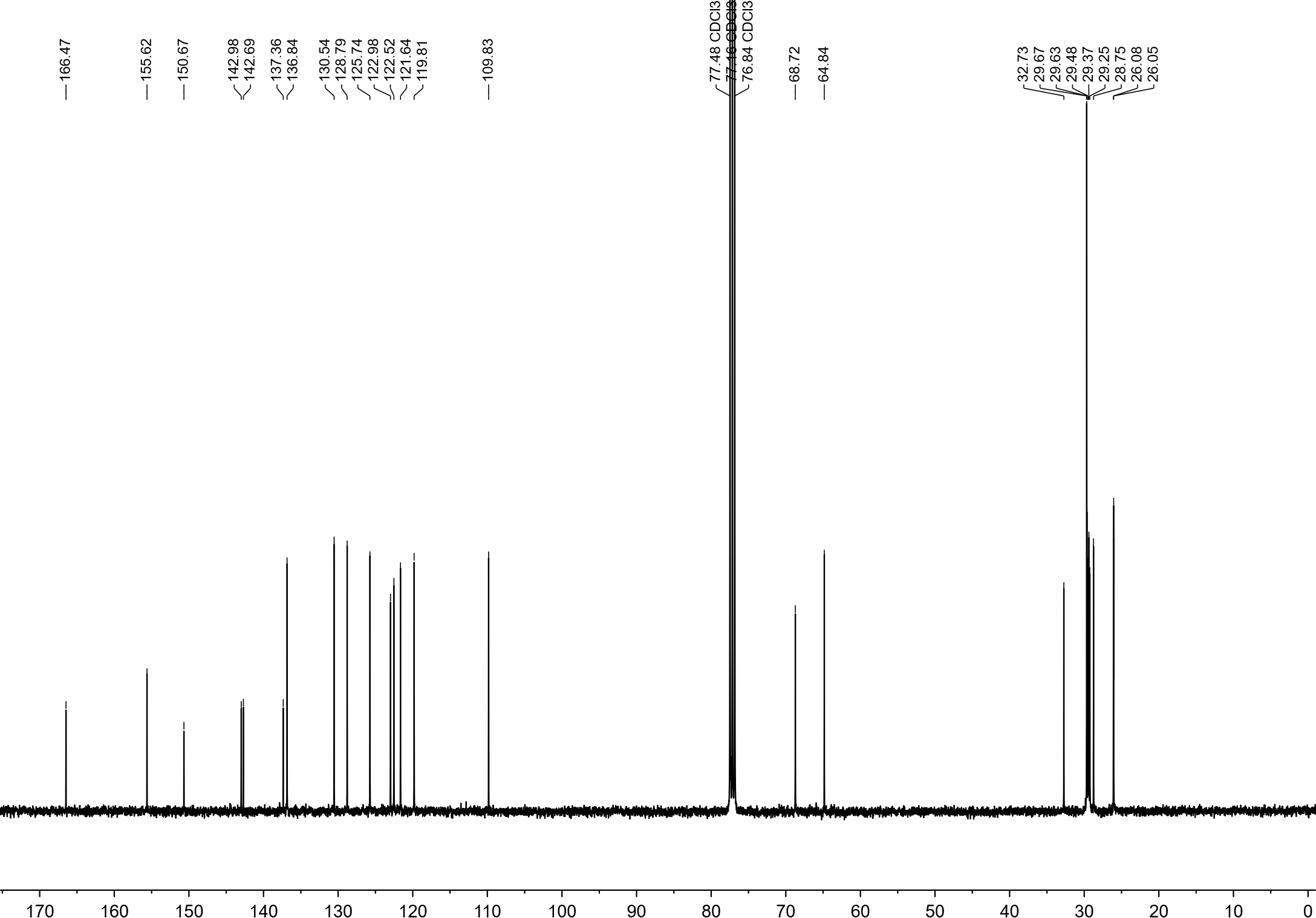


**Figure S21**. ^1^H-NMR (CDCl_3_, 400 MHz, top) and ^13^C-NMR (CDCl_3_, 100 MHz, bottom) spectra of **3, MBP-acrylate**.


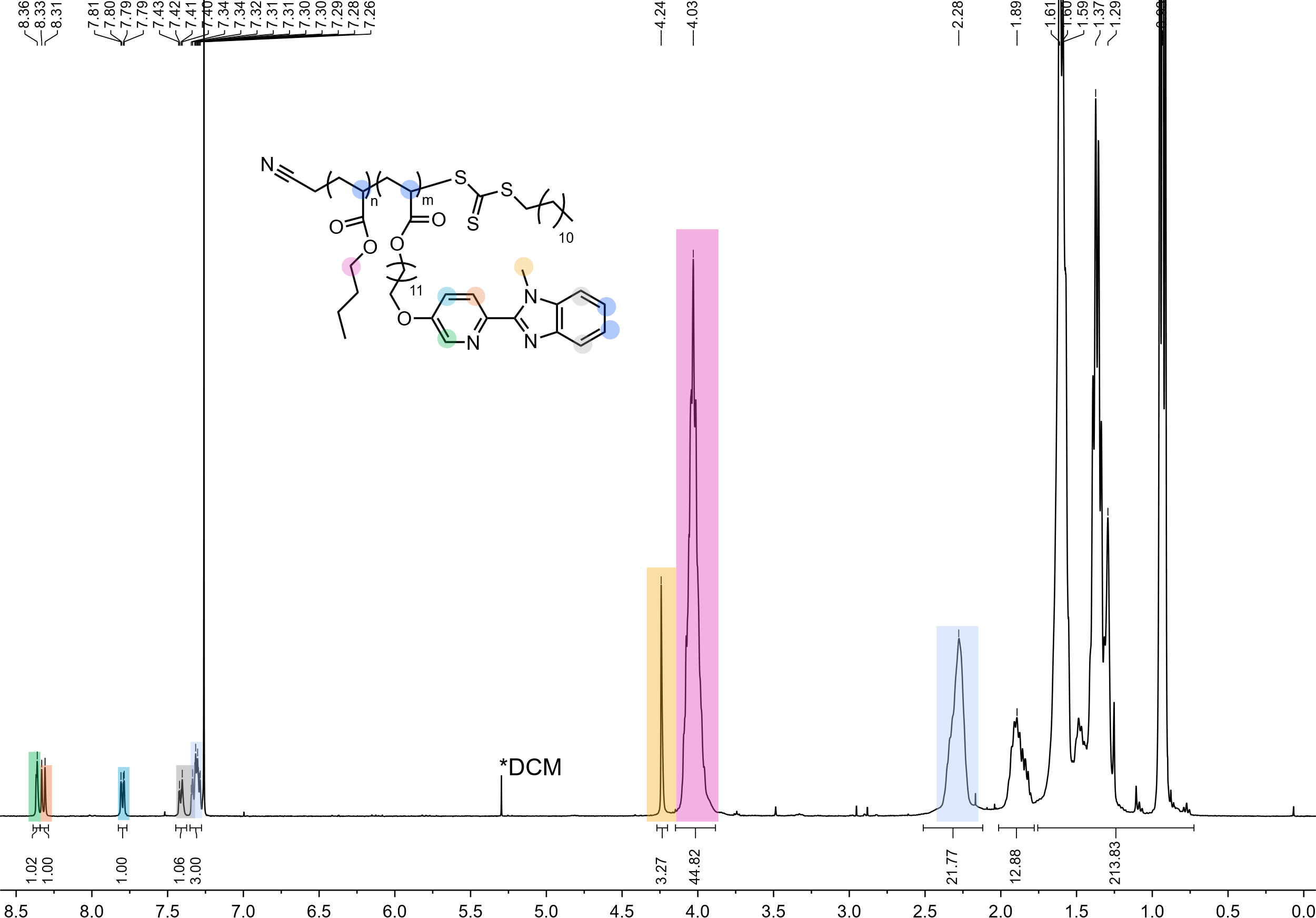


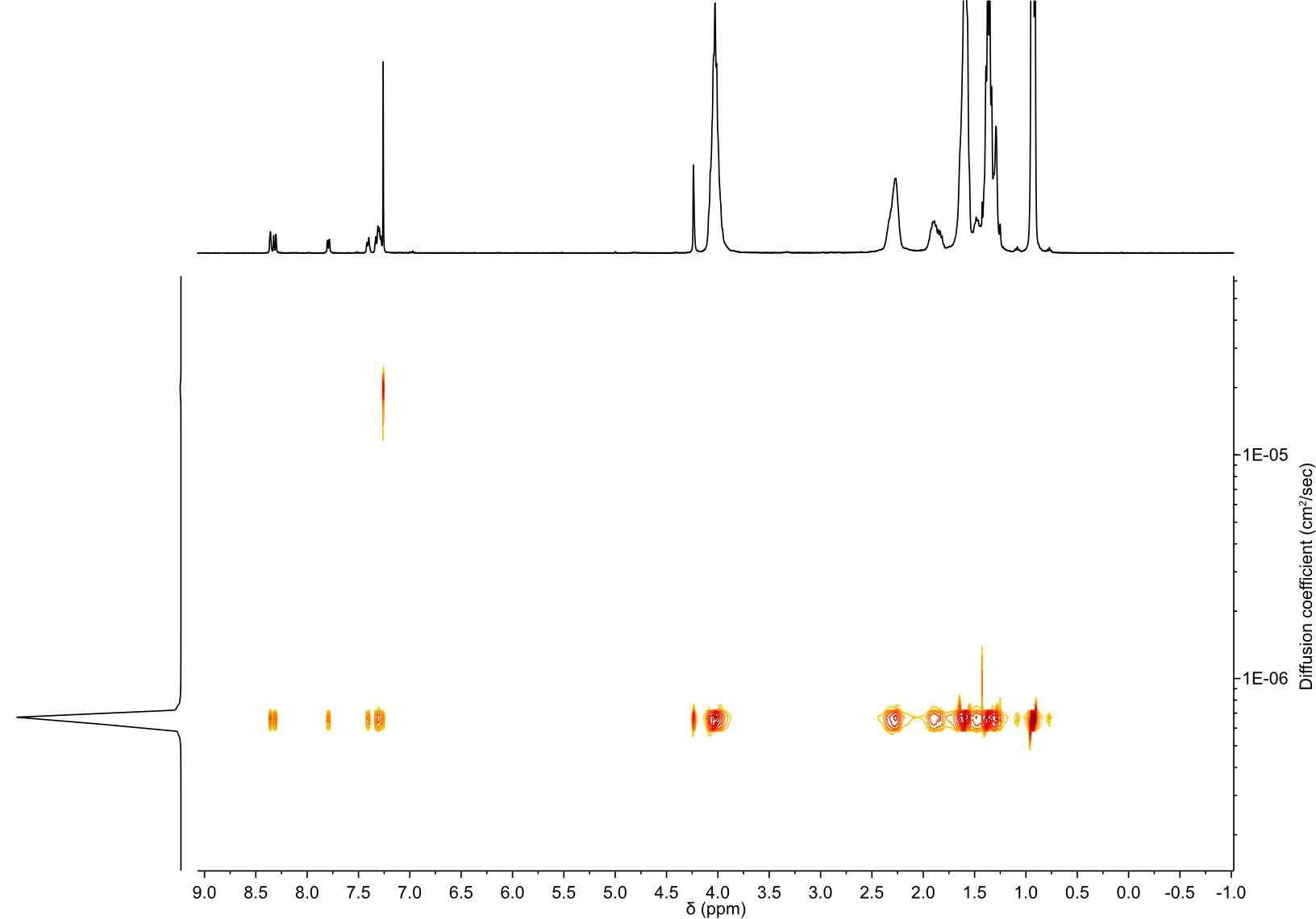


**Figure S22**. ^1^H-NMR (CDCl_3_, 400 MHz, top) and DOSY (CDCl_3_, 100 MHz, bottom) spectra of **PBA-***co***-MBP_5_-46**.


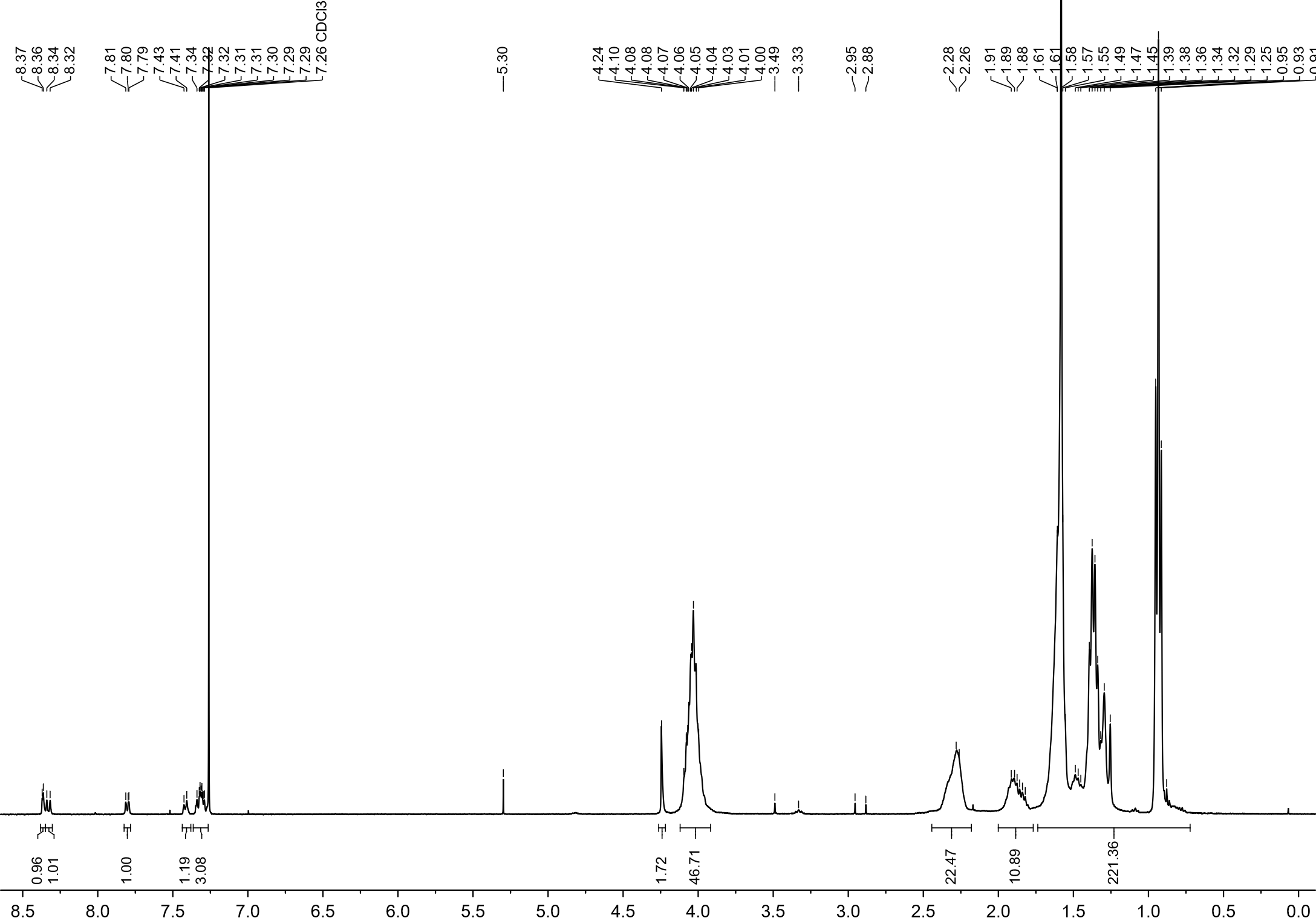


**Figure S23**. ^1^H-NMR (CDCl_3_, 400 MHz, top) spectra of **PBA-***co***-MBP_5_-16**.


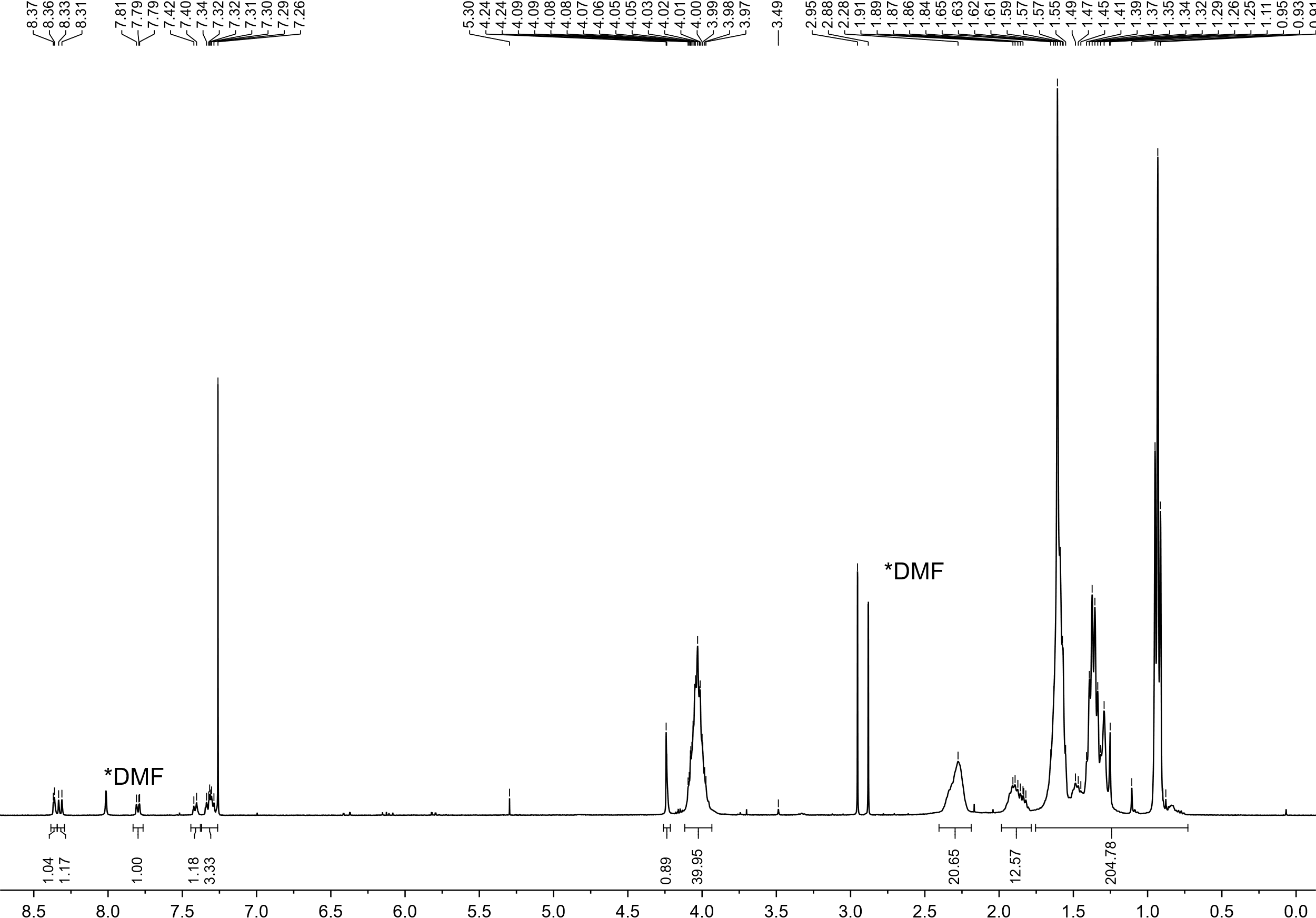


**Figure S24**. ^1^H-NMR (CDCl_3_, 400 MHz, top) spectra of **PBA-***co***-MBP_5_-28**.


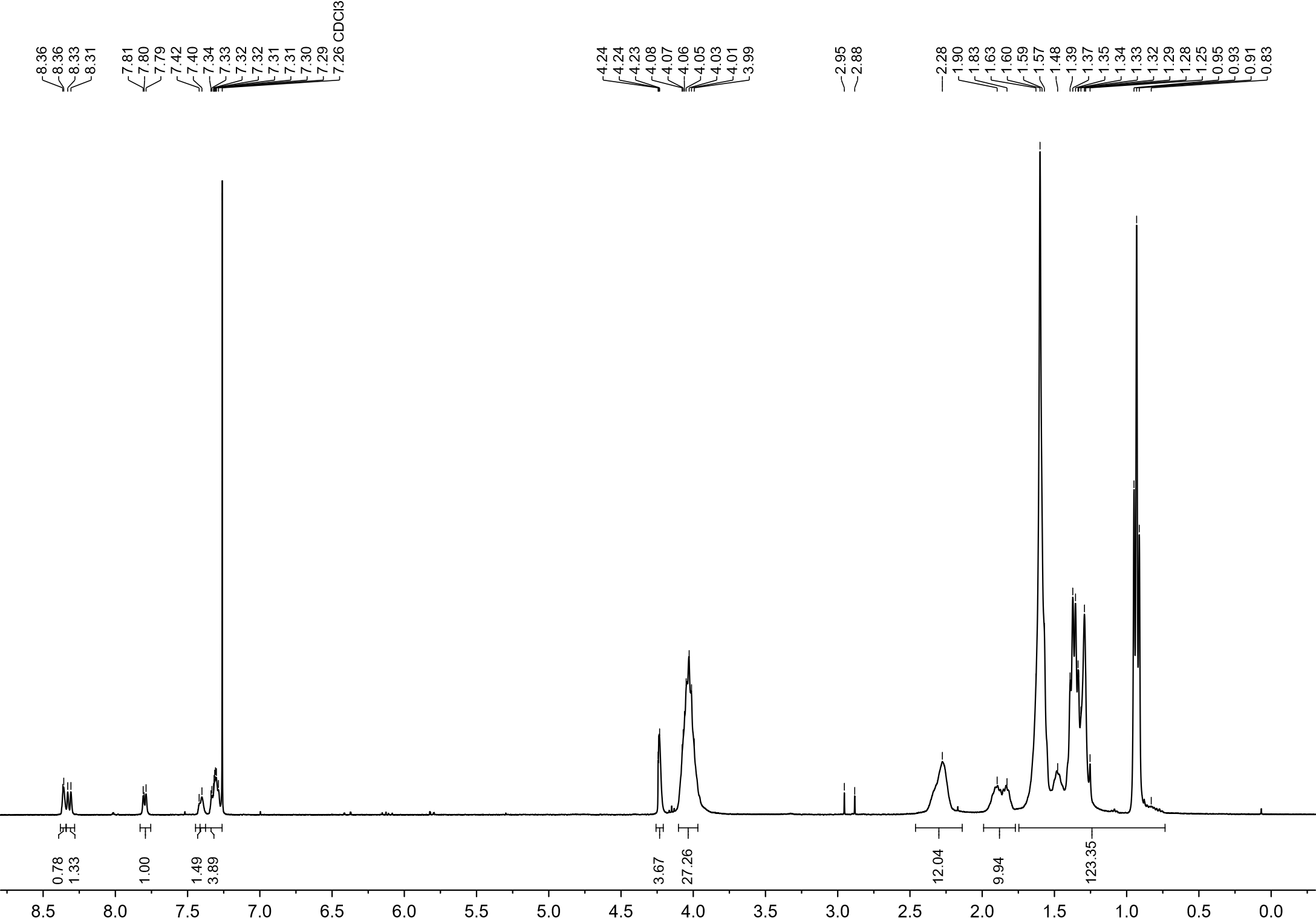


**Figure S25**. ^1^H-NMR (CDCl_3_, 400 MHz, top) spectra of **PBA-***co***-MBP_10_-56**.

**References**

1. Bertossi, L.; Oggioni, M.; Formon, G. J. M.; Weder, C., Light-Triggered Switching of Metallosupramolecular Polymer Systems. *ACS Macro Lett.* **2025,** *14* (6), 765-772.

2. Marx, F.; Beccard, M.; Ianiro, A.; Dodero, A.; Neumann, L. N.; Stoclet, G.; Weder, C.; Schrettl, S., Structure and Properties of Metallosupramolecular Polymers with a Nitrogen-Based Bidentate Ligand. *Macromolecules* **2023,** *56* (18), 7320-7331.
